# Statin Use and the Risk of Venous Thromboembolism in Women Taking Hormone Therapy

**DOI:** 10.1001/jamanetworkopen.2023.48213

**Published:** 2023-12-15

**Authors:** John W. Davis, Susan C. Weller, Laura Porterfield, Lu Chen, Gregg S. Wilkinson

**Affiliations:** 1 Department of Population Health Science, School of Public and Population Health, University of Texas Medical Branch, Galveston; 2 Sealy Institute for Vaccine Sciences, University of Texas Medical Branch, Galveston; 3 Department of Family Medicine, School of Medicine, University of Texas Medical Branch, Galveston

## Abstract

**Importance:**

Although hormone therapy (HT) in perimenopausal women is associated with increased risk for venous thromboembolism (VTE), it is unclear to what extent statins may mitigate this HT-associated risk.

**Objective:**

To estimate VTE risk in women aged 50 to 64 years taking HT with or without statins.

**Design, Setting, and Participants:**

This nested case-control study analyzed data from a commercially insured claims database in the US. Eligible participants included women aged 50 to 64 years with at least 1 year of continuous enrollment between 2008 and 2019. Data analysis occurred from January 2022 to August 2023.

**Exposure:**

Filled prescriptions for estrogens, progestogens, and statins were recorded in the 12 months prior to index. Recent HT was defined as any estrogen or progestogen exposure within 60 days before the index date. Current statin exposure was defined as 90 or more days of continuous exposure prior to and including the index date. Statin intensity was defined by the statin exposure 30 days prior to index.

**Main Outcomes and Measures:**

Cases were identified with VTE diagnoses (diagnostic codes) preceded by at least 12 months without VTE and followed within 30 days by anticoagulation, an inferior vena cava filter placement, or death. Controls were matched to cases (10:1) on date and age. Conditional logistic regression models estimated risk for HT and statin exposures with odds ratios (OR), adjusted for comorbidities. Conditional logistic regression models were used to estimate VTE risk for HT and statin exposures with odds ratios (ORs), adjusted for comorbidities. Intensity of statin therapy was measured as a subgroup analysis.

**Results:**

The total sample of 223 949 individuals (mean [SD] age, 57.5 [4.4] years) included 20 359 cases and 203 590 matched controls. Of the entire sample, 19 558 individuals (8.73%) had recent HT exposure and 36 238 individuals (16.18%) had current statin exposure. In adjusted models, individuals with any recent HT exposure had greater odds of VTE compared with those with no recent HT exposure (OR, 1.51; 95% CI, 1.43-1.60). Individuals receiving current statin therapy had lower odds of VTE compared with those with no current statin exposure (OR, 0.88; 95% CI, 0.84-0.93). When compared with those not recently taking HT or statins, the odds of VTE were greater for those taking HT without statins (OR, 1.53; 95% CI, 1.44-1.63) and for those taking HT with statins (OR, 1.25; 95% CI, 1.10-1.43), but were lower for those taking statins without HT (OR, 0.89; 95% CI, 0.85-0.94). Individuals taking HT with statin therapy had 18% lower odds of VTE than those taking HT without statins (OR, 0.82; 95% CI, 0.71-0.94) and there was greater risk reduction with higher intensity statins.

**Conclusions and Relevance:**

In this case-control study, statin therapy was associated with reduced risk of VTE in women taking HT, with greater risk reduction with high-intensity statins. These findings suggest that statins may reduce risk of VTE in women exposed to HT and that HT may not be contraindicated in women taking statins.

## Key Points

**Question:**

Is statin therapy associated with decreased risk of venous thromboembolism (VTE) in perimenopausal women exposed to hormone therapy (HT)?

**Findings:**

In this case-control study of 223 949 women aged 50 to 64 years, the risk of VTE was 53% higher in women recently exposed to HT without current statin therapy and 25% higher in women with recent HT exposure and current statin therapy compared with women who were not exposed to recent HT or statins.

**Meaning:**

These findings suggest statins are associated with reduced risk of VTE in women exposed to HT and that HT may not be contraindicated in women taking statins.

## Teaser

This case control study analyzes whether statins are associated with decreased risk of venous thromboembolism among perimenopausal women receiving hormone therapy.

## Introduction

Menopause-associated symptoms (eg, hot flashes, vaginal dryness, disruptions in sleep patterns, and cognitive changes) are common and can affect quality of life.^1,2,3^ Hormone therapy (HT) is an effective treatment for many of these symptoms.^4,5^ However, concerns regarding increased risk of venous thromboembolism (VTE), stroke, or myocardial infarction can prevent many symptomatic women from receiving HT.^6,7,8^ HT may double the risk of VTE,^9,10,11^ although the clinical trials were conducted with oral, conjugated equine estrogen (CEE),^9,12^ and newer studies suggest risk may be lower with other types of estrogen,^13,14,15,16,17^ routes of administration,^15,17,18,19^ and earlier initiation of therapy.^20,21,22^

In the decades since initial HT trials were conducted, statin therapy has been associated with reduced risk for major cardiovascular events^23,24^ and VTE,^25,26^ but few studies have estimated statin’s ability to mediate HT-associated VTE risk. Studies of concomitant HT and statin therapy suggest that the antiinflammatory and antithrombotic effects of statin therapy may mediate the risk of adverse cardiovascular events associated with HT. A UK study^27^ (1987-2008) in postmenopausal women found VTE risk from oral HT exposure was 51% higher for those not taking statin therapy and 21% higher for those taking statin therapy compared with those not exposed to oral HT. Similar reductions have been found for other adverse HT outcomes.^28,29,30^

Studies suggesting statins may mediate the risk of VTE associated with HT need validation in the US population because of different formulary, prescribing patterns and higher VTE incidence. VTE incidence in the UK ranged from 150 to 175 cases per 100 000 person-years between 2017 and 2019, whereas the US rate was 214 cases per 100 000 person-years between 2015 and 2019.^31^ Also, statin use among US women older than 40 years increased from 16% in 2002 to 2003 to 26% in 2012 to 2013,^32^ whereas the use of estrogen products (oral, vaginal, and transdermal) in women older than 50 years decreased from approximately 12.5% in 2006 to 9.2% in 2015, with larger reductions in oral estrogen products.^33^ Thus, this study explored whether statin therapy mediates VTE risk in US women aged 50 to 64 years exposed to exogenous hormones.

## Methods

### Data Set and Cohort Selection

This nested case-control study was approved by The University of Texas Medical Branch institutional review board with a waiver of informed consent because the data were deidentified, in accordance with 45 CFR §46. The study followed the Strengthening the Reporting of Observational Studies in Epidemiology (STROBE) reporting guideline^34^ and was designed within the Optum deidentified Clinformatics Data Mart Database containing claims for approximately 62 million unique enrollees. Excluding Medicare Advantage subscribers, there are approximately 15 million annual members, one-third of whom have had continuous enrollment for 3 or more years. Because VTE incidence is low, Clinformatics is 1 of the few US databases sufficiently large enough to study VTE risk factors. The database contains information on outpatient and inpatient diagnoses and filled prescriptions. The age and sex distribution in the database is similar to that of the US population, but historically minoritized racial and ethnic groups are slightly underrepresented (13% vs 19% Hispanic and 10% vs 14% Black).^35^

The study cohort was limited to women aged 50 to 64 years with more than 1 year of continuous enrollment between January 1, 2007, and December 31, 2019. Observations without sex (0.01%) or age (0.00007%) were excluded. Cases and matched controls were selected between January 1, 2008, and December 1, 2019, to allow a 12-month look-back period and 30-day follow-up period. The age range was selected because of the average age at menopause,^36^ higher rates of menopausal HT use in this age range,^33^ and the tendency to transition from commercial insurance to Medicare at age 65 years. The study focused on statin therapy and HT as part of a larger project on VTE; design details, including *International Classification of Diseases, Ninth Revision [ICD-9]*, *International Statistical Classification of Diseases and Related Health Problems, Tenth Revision (ICD-10)*, and *Current Procedural Terminology* codes were reported previously.^15^

### Cases and Controls

Cases were defined as women with a VTE diagnostic code during the observation period followed by a prescription for an anticoagulant (excluding heparin flushes), intravascular vena cava filter, or death within 30 days of the VTE diagnosis (index date).^31^ Controls were randomly sampled (without replacement) and matched to cases by index date (month) and age (±2 years) at a 10:1 ratio. A large ratio was used to estimate low-frequency categories. Women were excluded if they had less than 12 months of enrollment data, an acute or chronic VTE diagnosis, an intravascular vena cava filter placement in the year prior to the index date, or exposure to anticoagulants within 14 days prior to the index date.

### HT Exposure

HT exposure was defined as a filled prescription for any estrogen and/or progestogen, and the duration of the prescription (30 or 90 days) was used to determine timing of exposure. Exposure was evaluated regardless of route (oral or nonoral) or indication (eg, menopausal HT or contraception). Timing of HT exposure was defined as recent (≤60 days before index) vs past and none, because previous research indicated VTE risk diminishes after 60 days.^15^ National drug codes for hormones were identified using Red Book^37^ and/or from a list supplied by the US Food and Drug Administration^33^ (list published previously^15^).

### Statin Exposure

Statin exposure was defined by duration and continuity of filled prescriptions. Current statin exposure was defined as continuous filled prescriptions for 90 days or more prior to and including the index date. The comparison category was shorter, discontinuous, or no exposure in the past year. Intensity of therapy 30 days prior to the index date was defined as high (atorvastatin ≥40 mg or rosuvastatin ≥20 mg) or low to moderate (all other doses) according to American Heart Association guidelines.^38^ Red Book^37^ national drug codes were used to identify statin prescriptions, formulations, and doses (eTable 1 in Supplement 1).

### Statistical Analysis

Risk for VTE with statin and/or HT exposures was estimated with adjusted odds ratios (ORs) and 95% CIs.^39^ Because of the matched design, conditional logistic regression models were used.^17,27,30^ Covariates included region of residence, age at index date, history of cancer except for nonmelanoma skin cancer (categorized as no cancer, nonmetastatic cancer, and metastatic cancer), history of prothrombotic conditions or other types of thrombosis (ie, superficial thrombophlebitis), varicose veins within the previous year, and hospitalization (with or without surgery) or trauma within 30 days of the index date. In addition, coronary artery disease, stroke, lipid disorders, and smoking within the past year were included because they may affect the likelihood of prescribing HT or statin therapy. Finally, the Elixhauser set of comorbidities was included to control for general health status.^40^ The Elixhauser Comorbidity Index^41^ has been validated for estimating mortality and includes chronic conditions such as obesity, liver disease, and diabetes. Known factors associated with increased risk of VTE (eg, cancers and hypercoagulable conditions) were removed from the index and used as separate covariates. The final Elixhauser Comorbidity Index was summed and coded into terciles (0 comorbidities, 1-2 comorbidities, and ≥3 comorbidities).

Models estimated the association of HT and statin exposures with VTE risk, controlling for covariates. Sensitivity analyses examined the stability of main associations across stratified subsamples with different comorbidity burdens. Because conditional and unconditional analyses can yield similar results when few matching criteria are used,^42^ unconditional analyses were used on stratified subsamples to avoid loss of observations. An interaction term in the full sample with a conditional analysis assessed whether association modification occurred between statin and HT exposures.

To estimate the combined association of current statin and recent HT exposures across the distribution of risk factors, women were coded into 4 groups by their HT and statin exposure status. ORs were estimated for HT exposure without statin therapy, HT exposure with statin therapy, and statin therapy without HT exposure with reference to women with neither recent HT nor current statin therapy. Another model estimated the association of statin intensity with additional subgroups for lower and higher intensity therapy. A 2-sided *P* < .05 was considered statistically significant. All statistical analyses were conducted using SAS statistical software version 9.22 (SAS Institute). Data analysis occurred from January 2022 to August 2023.

## Results

### Sample Characteristics

Of the 74 600 patients identified with a first, acute VTE and 22 380 610 eligible controls in the Clinformatics database, a total of 223 949 women (mean [SD] age, 57.5 [4.4] years) were included; we identified 20 359 cases that met inclusion criteria, and all were successfully matched to 203 590 controls. The flowchart for sample selection was published previously.^15^ Women in the study sample were approximately equally distributed in 5-year or 6-year age intervals (50-54 years, 55-60 years, and 61-65 years). Cases had a higher proportion of comorbidities and factors associated with increased risk of VTE than controls (Table 1). A total of 11 389 cases (55.94%) and 45 700 controls (22.45%) had 3 or more comorbidities; 5294 cases (26.00%) and 11 370 controls (5.58%) had cancer; 2743 cases (13.47%) and 9775 controls (4.80%) had coronary artery disease; and 4710 cases (23.13%) and 19 263 controls (9.46%) smoked. Among the VTE cases, 10 995 (54.01%) had pulmonary emboli (with or without deep vein thrombosis), 9364 (45.99%) had deep vein thrombosis without pulmonary emboli, and 1197 (5.88%) died within 30 days following diagnosis.

**Table 1.** Sample Description

Variable	Participants, No. (%) (N = 223 949)	
	Venous thromboembolism cases (n = 20 359)	Controls (n = 203 590)
Venous thromboembolism index date		
2007-2010	4951 (24.32)	49 510 (24.32)
2011-2014	6747 (33.14)	67 470 (33.14)
2015-2019	8661 (42.54)	86 610 (42.54)
Age, y		
50-54	6789 (33.35)	70 927 (34.84)
55-60	7084 (34.80)	68 237 (33.52)
61-65	6486 (31.86)	64 426 (31.64)
Region		
Northeast	1686 (8.28)	18 010 (8.85)
Midwest	5661 (27.81)	52 021 (25.55)
South	8912 (43.77)	91 138 (44.77)
West	4067 (19.98)	40 947 (20.11)
Unknown	33 (0.16)	1474 (0.72)
Comorbidities, No.		
0	2812 (13.81)	75 981 (37.32)
1-2	6158 (30.25)	81 909 (40.23)
≥3	11 389 (55.94)	45 700 (22.45)
Cancer		
None	15 065 (74.00)	192 220 (94.42)
Solid tumor	2262 (11.11)	9764 (4.80)
Metastatic	3032 (14.89)	1606 (0.79)
Trauma	3060 (15.03)	6030 (2.96)
Hospitalization or surgery	6152 (30.22)	4672 (2.29)
Hypercoagulable condition	2237 (10.99)	2551 (1.25)
Varicose veins	556 (2.73)	1790 (0.88)
Coronary artery disease	2743 (13.47)	9775 (4.80)
Stroke	1495 (7.34)	4886 (2.40)
Hyperlipidemia	9402 (46.18)	76 043 (37.35)
Smoking	4710 (23.13)	19 263 (9.46)

### Statin and Hormone Exposures

Recent exposures to any HT occurred in 19 558 individuals (8.73%) including 2130 cases (10.46%) and 17 428 controls (8.56%). Of those exposures, 14 810 (75.72%) were menopausal estrogen or estrogen-progestogen combinations (10 585 [71.47%] oral and 4223 [28.51%] nonoral), 2476 (12.66%) were estrogen-progestin contraceptives, 1486 (7.60)% were progesterone only, and 786 (4.02%) were estrogen-testosterone combinations. Those with recent HT exposure tended to be younger (69 272 [33.89%] unexposed and 8444 [43.17%] exposed at <55 years of age).

For statin therapy, 36 238 individuals in the entire sample (16.18%), including 3717 cases (18.26%) and 32 521 controls (15.97%), had current or continuous exposure for at least 3 months before and including the index date; 17 569 (7.85%) had shorter or discontinuous exposures; and 170 142 (75.97%) had no exposure in the past year. Shorter or intermittent exposure was not significantly different from no exposure (OR, 1.05; 95% CI, 0.99-1.12) but was significantly different from 3 or more months of current or continuous exposure (OR, 1.18; 95% CI, 1.10-1.25), indicating that the intermittent and no exposure groups could be combined. Those with current or continuous statin exposure tended to be older (54 508 [29.04%] unexposed and 16 404 [45.27%] exposed at >60 years of age), have hyperlipidemia diagnoses (54210 [28.88%] unexposed and 31 235 [86.19%] exposed), or have coronary artery disease (7501 [4.00%] unexposed and 5017 [13.84%] exposed) than those without current or continuous exposure. Among those currently exposed to statins, 29 011 (80.06%) were exposed to low or moderate intensity statins and 7227 (19.94%) were exposed to high intensity statins in the 30 days prior to the index date.

After adjustment for all covariates in a conditional model, the OR for any recent HT exposure (regardless of statin exposure) was elevated 51% compared with those without recent HT exposure (model 1 OR, 1.51; 95% CI, 1.43-1.60) (Table 2). The OR for current or continuous statin therapy (regardless of HT exposure) was 12% lower than less or no statin therapy (OR, 0.88; 95% CI, 0.84-0.93). There was slightly less risk reduction with low or moderate intensity statin therapy (OR, 0.90; 95% CI, 0.85-0.95) and slightly more risk reduction with high-intensity statin therapy (OR, 0.82; 95% CI, 0.75-0.90) compared with those without current statin exposure (not shown). Results for HT and statin therapy were consistent across the total sample with and without a conditional analysis, between the total sample and subsamples without cancer or hypercoagulability conditions, and subsamples with above and below median comorbidity burdens (Table 2). The singular exception was the higher association of HT exposure in subsamples with fewer comorbidities. There was no significant interaction of HT and statin therapy with VTE risk.

**Table 2.** Subanalyses Stratified by Comorbidities

Variable	Model, OR (95% CI)				
	1: Full sample (conditional)^a^	2: Full sample (unconditional)^b^	3: Cancer and hypercoagulable condition exclusion (unconditional)^c^	4: Stratified ECI score <2 (unconditional)^d^	5: Stratified ECI score >2 (unconditional)^d^
Case count, No.	20 359	20 359	13 835	5850	14 509
Control count, No.	203 590	203 590	190 170	124 217	79 373
HT exposure					
None or past (61-365 d) (n = 204 391)	1 [Reference]	1 [Reference]	1 [Reference]	1 [Reference]	1 [Reference]
Any recent (0-60 d) (n =19 558)	1.51 (1.43-1.60)	1.51 (1.43-1.59)	1.56 (1.48-1.65)	1.90 (1.76-2.07)	1.26 (1.17-1.35)
Statin exposure					
<3 mo Or none (n =187 111)	1 [Reference]	1 [Reference]	1 [Reference]	1 [Reference]	1 [Reference]
≥3 mo (n =36 238)	0.88 (0.84-0.93)	0.88 (0.84-0.92)	0.91 (0.86-0.96)	0.90 (0.81-1.01)	0.89 (0.85-0.94)

Abbreviations: ECI, Elixhauser Comorbidity Index; HT, hormone therapy; OR, odds ratio.

^a^ Conditional model was adjusted for age, ECI score, region, cancer, hospitalization or surgery, trauma, hypercoagulability, varicose veins, coronary artery disease, stroke, lipid disorders, and smoking history.

^b^ Unconditional model was adjusted for age, ECI score, region, cancer, hospitalization or surgery, trauma, hypercoagulability, varicose veins, coronary artery disease, stroke, lipid disorders, smoking history, and index date.

^c^ Unconditional model was adjusted for age, comorbidity score, region, hospitalization or surgery, trauma, varicose veins, coronary artery disease, stroke, lipid disorders, smoking history, and index date.

^d^ Unconditional model was adjusted for age, region, cancer, hospitalization or surgery, trauma, hypercoagulability, varicose veins, coronary artery disease, stroke, lipid disorders, smoking history, and index date.

### Patient Groups With HT and/or Statin Exposures

The combined main effects of statin and HT were estimated for the 4 groups created by HT and statin exposures. As hypothesized, the OR was highest for women exposed to HT without statin therapy compared with the reference group with neither recent HT nor statin exposure (model 1 in Table 3). The OR for the 16 350 individuals with HT exposure but without statin therapy was elevated by 53% over the 171 361 individuals in the reference group (OR, 1.53; 95% CI, 1.44-1.63). The OR for the 3208 individuals with HT exposure combined with statin therapy was 25% higher than that for the reference group (OR, 1.25; 95% CI, 1.10-1.43). A direct comparison between those receiving HT with statin therapy and those receiving HT without statin therapy showed a significantly lower (18%) OR for those with statin therapy (OR, 0.82; 95% CI, 0.71-0.94). Finally, the lowest risk was for those exposed to statin therapy without HT exposure; the OR was 11% lower than the reference group (OR, 0.89; 95% CI, 0.85-0.94). See eTable 2 in Supplement 1 for risk factor distributions for the 4 exposure groups.

**Table 3.** Odds of Venous Thromboembolism for Patient Subgroups by Recent HT and Statin Exposures

Exposure	Total participants, No.	Cases, No.	Controls, No.	Adjusted model 1, OR (95% CI)^a^	Adjusted model 2 with statin intensity, OR (95% CI)^a^
No recent HT and no current statin exposure	171 361	14 865	156 496	1 [Reference]	1 [Reference]
Recent HT (without current statin exposure)	16 350	1777	14 573	1.53 (1.44-1.63)^b^	1.53 (1.44-1.63)^c^^,^^d^
Recent HT with current statin exposure	3208	353	2855	1.25 (1.10-1.43)^b^	NA
Low or moderate statin exposure	2668	295	2373	NA	1.29 (1.12-1.49)^c^
High statin exposure	540	58	482	NA	1.06 (0.77-1.45)^d^
Current statin (without recent HT)	33 030	3364	29 666	0.89 (0.85-0.94)	NA
Low or moderate statin exposure	26 343	2599	23 744	NA	0.91 (0.86-0.96)
High statin exposure	6687	765	5922	NA	0.84 (0.76-0.92)

Abbreviations: HT, hormone therapy; NA, not applicable; OR, odds ratio.

^a^ ORs have been adjusted for age, Elixhauser Comorbidity Index score, region, cancer, hospitalization or surgery, trauma, hypercoagulability, varicose veins, coronary artery disease, stroke, lipid disorders, and smoking history.

^b^ A direct comparison between recent HT with and without current statin exposure showed a 18% significantly lower OR with statin therapy (OR, 0.82; 95% CI, 0.71-0.94).

^c^ A direct comparison between HT without statin therapy and HT with low or moderate statin intensity showed a 16% reduction (OR, 0.84; 95% CI, 0.73-0.98).

^d^ A direct comparison between HT without statin therapy and high statin intensity showed a 31% reduction (OR, 0.69; 95% CI, 0.50-0.95).

When subgroups exposed to statins were subdivided into low and moderate vs high-intensity therapy, a larger protective association was observed with high-intensity therapy compared with the reference group without recent HT or statin exposure (model 2 in Table 3). ORs were elevated 53% with recent HT exposure without statins (OR, 1.53; 95% CI, 1.44-1.63) and were elevated 29% with low and moderate statin intensity (OR, 1.29; 95% CI, 1.12-1.49), but were not elevated with high statin intensity (OR, 1.06; 95% CI, 0.77-1.45) compared with those without recent HT and without current statin therapy. A direct comparison between HT without statin therapy and HT with low and moderate statin intensity showed a 16% reduction (OR, 0.84; 95% CI, 0.73-0.98), and a comparison with HT with high statin intensity showed a 31% reduction (OR, 0.69; 95% CI, 0.50-0.95). The OR for those without recent HT exposure showed a 9% reduction with low and moderate intensity but a 16% reduction with high-intensity statin therapy (OR, 0.84; 95% CI, 0.76-0.92) compared with the reference group without HT and without statin therapy.

## Discussion

To our knowledge, this is the first large case-control study in a US claims database to assess the association of risk of VTE with HT and statin exposures in women of postmenopausal age. In weighing the benefits of menopausal symptom relief against reported risks of VTE from clinical trials^6,10^ and cohort studies,^11,17^ this study provides insight into additional factors associated with the risk profile of HT users. Specifically, we found that the risk-benefit profile for HT may be more favorable when taking concomitant statin therapy. In this study, results indicated HT-associated VTEs may be reduced with statin therapy. Recent HT exposure elevated risk 53% without statin exposure, but elevated risk 25% when combined with statin therapy compared with the reference group without HT and without statin exposure—a significant 18% reduction. Higher intensity statin therapy reduced risk 31% compared with the reference group and mitigated HT-associated risk, providing evidence of a dose-response effect in the association.

The overall risk reduction for those with recent HT and concomitant statin therapy was similar to previous studies,^27,28^ although observational studies conducted 2 decades ago were limited by the low prevalence of statin usage. This study and a UK study^27^ each showed an almost 20% reduction in HT risk with statin therapy, although the UK study used an earlier time period (1987-2008), broader age range (50-79 years), and focused on oral menopausal HT exposures. In contrast with this study, the UK study^27^ did not detect significant reductions with higher intensity statins. The difference may be due to different definitions of statin intensity and/or the proportion of women exposed to statins. Similarly, the reanalysis of the Heart and Estrogen/Progestin Replacement study cohort^28^ showed VTE risk from HT was 75% greater than placebo (hazard ratio, 1.75; *P* = .04) for those unexposed to statins, but women exposed to both statins and HT had a lower, nonsignificant 34% elevation in risk compared with placebo (hazard ratio, 1.34; *P* = .45), suggesting insufficient statistical power.

The overall 51% increase in risk for VTE with HT exposure found in this study is consistent with other studies. A large UK case-control study^17^ found any menopausal HT exposure in the past 90 days increased the OR by 43%, oral exposures increased the OR by 58%, and CEE exposures almost doubled the OR compared with no HT exposure. Clinical trial estimates indicating HT may double the risk for VTE^9,10^ may be higher because of older age of HT initiation and use of oral CEE.^15,17^ This study therefore affirms and expands previous findings.

In this study, the HT-associated risk of VTE was slightly higher for those with fewer comorbidities and slightly lower for those with more comorbidities compared with no HT exposure. This difference was likely due to greater exposure to contraceptives (and higher risk estrogen and progestin formulations) in the younger US women with fewer comorbidities.^15^ A notable difference between this sample and the UK samples^17,27^ was the number of women in the US sample on higher-risk combined hormone contraceptives.^15^ Women older than 50 years exposed to combined hormonal contraceptives had 5 to 9 times higher risk of VTE compared with those with no hormone exposure.^15^

The overall reduction in VTE risk with statin therapy observed in this study (12%) was smaller than estimated in previous observational studies but similar to the reduction found in randomized clinical trials (RCTs). Cohort studies^25^ suggest statin exposure may reduce VTE risk by 25%, whereas RCTs indicate a 15% reduction in risk^25^ and accentuation by intensity of therapy.^25,26,43,44^ Differences between cohort and RCT findings may be attributable to differences in patient populations,^45^ as well as duration of statin exposure. Statin effectiveness studies typically have a longer median statin exposure time (approximately 4 years)^25^ than in this study, where VTE reduction was observed with 90 or more days. In this study, statin therapy did not interact with HT, but instead had independent associations with reduction in VTE.

### Limitations

There are several limitations to this study. Most importantly, this study is a nested case-control design in an administrative claims database and contains all inherent limitations of a secondary, observational data set. Although the study cannot directly prove causality, the time-restricted design offered an unbiased estimation of exposure risk for the population in the base cohort. Misclassification was minimized by using a strict case definition. Although other studies^17,27,30,46^ included probable cases in their analyses, we combined diagnostic claim codes with confirmatory events (ie, hospitalization, death, or anticoagulant prescription within 30 days of diagnosis) to increase diagnostic accuracy and detection of definite cases.^47^ We also adjusted for comorbidities that could potentially confound estimates and controlled for coronary artery disease, stroke, and lipid disorders to minimize indication bias. However, even with statistical controls to minimize bias, some residual confounding may remain when treatment groups are not randomly assigned.

Other limitations include a limited look-back period, rather than a lifetime medical history, and limited information on patient characteristics. The database contained data on patient age, sex, and region of residence, but lacked data on race and ethnicity, income, educational level, and indications for prescriptions. Race and ethnicity is associated with VTE,^48,49^ but may be mediated by comorbidities, region, and/or socioeconomic status.^49^ In this study, we controlled for comorbidities and region of residence. Socioeconomic status was indirectly controlled for because all patients had private health insurance. The data set also did not have information on over-the-counter medications (such as aspirin), which may^50^ or may not^51^ have an independent protective association with VTE in this population.

Despite these limitations, medication and diagnostic detail contained in the large administrative claims database allowed for the estimation of associations for low prevalence exposures and outcomes. This was especially true for estimating risk for those with both hormone and statin exposures. Furthermore, there were sufficient women to make inferences about intensity of statin therapy.

## Conclusions

In this case-control study of women with VTE and matched controls, we found statin therapy was associated with reduced, but not eliminated, risk of VTE associated with exogenous hormones. Although women who are at higher cardiovascular risk may take statins, and thus may seem unlikely candidates for HT, statin therapy appears to mitigate some of the risk from HT. HT may not be contraindicated in women who are candidates for statin therapy. These findings suggest that statins may improve the HT risk-benefit profile for women with perimenopausal symptoms. An RCT evaluating the absolute risk of thrombosis with concomitant statin therapy is needed to elucidate the full safety profile.

## Notes

Supplement 1. **eTable 1.** List of Statin Exposures With NDC and Dose **eTable 2.** Demographics and Comorbidities for the Four Exposure Groups

Supplement 2.
Data Sharing Statement
