# Error in Discussion

**DOI:** 10.1001/jamanetworkopen.2024.9013

**Published:** 2024-03-29

In the Original Investigation titled “Statin Use and the Risk of Venous Thromboembolism in Women Taking Hormone Therapy,”^1^ published December 15, 2023, the second paragraph of the Discussion reported results from the Heart and Estrogen/Progestin Replacement cohort re-analysis describing incidence of coronary death and nonfatal myocardial infarction rather than results describing the incidence of venous thromboembolism. This article has been corrected.^1^
