# Supplementary material for: Statin Use and the Risk of Venous Thromboembolism in Women Taking Hormone Therapy
Source: JAMA Netw Open. 2023 Dec 15;6(12):e2348213. doi: 10.1001/jamanetworkopen.2023.48213 (PMC10724767; doi:10.1001/jamanetworkopen.2023.48213)
Supplement: Supplement 1. — eTable 1. List of Statin Exposures With NDC and Dose eTable 2. Demographics and Comorbidities for the Four Exposure Groups [file jamanetwopen-e2348213-s001.pdf]

Supplemental Online Content

Davis JW, Weller SC, Porterfield L, Chen L, Wilkinson GS. Statin use and the risk of venous thromboembolism in women taking hormone therapy. *JAMA Netw Open*. 2023;6(12):e2348213. doi:10.1001/jamanetworkopen.2023.48213

eTable 1. List of Statin Exposures with NDC and Dose
eTable 2. Demographics and Comorbidities for the Four Exposure Groups

This supplemental material has been provided by the authors to give readers additional information about their work.

© 2023 Davis JW et al. *JAMA Network Open.*

eTable 1. List of Statin Exposures with NDC and Dose

List of Statin Exposures

NDC	Name	Dose
**00026288351**	BAYCOL	0.2 MG
**00026288386**	BAYCOL	0.2 MG
**00026288451**	BAYCOL	0.3 MG
**00026288486**	BAYCOL	0.3 MG
**54569458900**	BAYCOL	0.3 MG
**00026288551**	BAYCOL	0.4 MG
**00026288569**	BAYCOL	0.4 MG
**00026288586**	BAYCOL	0.4 MG
**54569486100**	BAYCOL	0.4 MG
**54868443600**	BAYCOL	0.4 MG
**00026288669**	BAYCOL	0.8 MG
**00026288686**	BAYCOL	0.8 MG
**54569518000**	BAYCOL	0.8 MG
**54868440100**	BAYCOL	0.8 MG
**00002477090**	LIVALO	1 MG
**25208020009**	ZYPITAMAG	1 MG
**66869010490**	LIVALO	1 MG
**00071015523**	LIPITOR	10 MG
**00071015534**	LIPITOR	10 MG
**00071015540**	LIPITOR	10 MG
**00093505698**	ATORVASTATIN CALCIUM	10 MG
**00378201505**	ATORVASTATIN CALCIUM	10 MG
**00378201577**	ATORVASTATIN CALCIUM	10 MG
**00378395005**	ATORVASTATIN CALCIUM	10 MG
**00378395007**	ATORVASTATIN CALCIUM	10 MG
**00378395009**	ATORVASTATIN CALCIUM	10 MG
**00378395077**	ATORVASTATIN CALCIUM	10 MG
**00591377410**	ATORVASTATIN CALCIUM	10 MG
**00591377419**	ATORVASTATIN CALCIUM	10 MG
**00781538192**	ATORVASTATIN CALCIUM	10 MG
**00904629061**	ATORVASTATIN CALCIUM	10 MG
**10135064910**	ATORVASTATIN CALCIUM	10 MG
**13411011301**	LIPITOR	10 MG
**13411011303**	LIPITOR	10 MG
**13411011306**	LIPITOR	10 MG
**13411011309**	LIPITOR	10 MG
**13411011315**	LIPITOR	10 MG

© 2023 Davis JW et al. *JAMA Network Open.*

eTable 1. List of Statin Exposures with NDC and Dose

List of Statin Exposures

NDC	Name	Dose
**16714087401**	ATORVASTATIN CALCIUM	10 MG
**16714087402**	ATORVASTATIN CALCIUM	10 MG
**16714087403**	ATORVASTATIN CALCIUM	10 MG
**16729004417**	ATORVASTATIN CALCIUM	10 MG
**33261095900**	ATORVASTATIN CALCIUM	10 MG
**33261095930**	ATORVASTATIN CALCIUM	10 MG
**33261095960**	ATORVASTATIN CALCIUM	10 MG
**33261095990**	ATORVASTATIN CALCIUM	10 MG
**33358021001**	LIPITOR	10 MG
**33358021030**	LIPITOR	10 MG
**33358021060**	LIPITOR	10 MG
**33358021090**	LIPITOR	10 MG
**35356086018**	ATORVASTATIN CALCIUM	10 MG
**35356086030**	ATORVASTATIN CALCIUM	10 MG
**35356086090**	ATORVASTATIN CALCIUM	10 MG
**42254030730**	ATORVASTATIN CALCIUM	10 MG
**42254039130**	ATORVASTATIN CALCIUM	10 MG
**42291014310**	ATORVASTATIN CALCIUM	10 MG
**42291014390**	ATORVASTATIN CALCIUM	10 MG
**43063037330**	ATORVASTATIN CALCIUM	10 MG
**43063047430**	ATORVASTATIN CALCIUM	10 MG
**49999039230**	LIPITOR	10 MG
**49999039290**	LIPITOR	10 MG
**50090125400**	ATORVASTATIN CALCIUM	10 MG
**50090125401**	ATORVASTATIN CALCIUM	10 MG
**50090125500**	ATORVASTATIN CALCIUM	10 MG
**50090125501**	ATORVASTATIN CALCIUM	10 MG
**50268009311**	ATORVASTATIN CALCIUM AVPAK	10 MG
**50268009315**	ATORVASTATIN CALCIUM AVPAK	10 MG
**51079020801**	ATORVASTATIN CALCIUM	10 MG
**51079020820**	ATORVASTATIN CALCIUM	10 MG
**51079040901**	ATORVASTATIN CALCIUM	10 MG
**51079040920**	ATORVASTATIN CALCIUM	10 MG
**51407007810**	ATORVASTATIN CALCIUM	10 MG
**51407007890**	ATORVASTATIN CALCIUM	10 MG
**51655022624**	LIPITOR	10 MG
**51655061030**	ATORVASTATIN CALCIUM	10 MG
**51655061352**	ATORVASTATIN CALCIUM	10 MG
**51655061452**	ATORVASTATIN CALCIUM	10 MG

© 2023 Davis JW et al. *JAMA Network Open.*

eTable 1. List of Statin Exposures with NDC and Dose

List of Statin Exposures

NDC	Name	Dose
52959075990	LIPITOR	10 MG
54569446600	LIPITOR	10 MG
54569446601	LIPITOR	10 MG
54569446602	LIPITOR	10 MG
54569628200	ATORVASTATIN CALCIUM	10 MG
54569628201	ATORVASTATIN CALCIUM	10 MG
54868393400	LIPITOR	10 MG
54868393401	LIPITOR	10 MG
54868393402	LIPITOR	10 MG
54868393403	LIPITOR	10 MG
54868393404	LIPITOR	10 MG
54868631900	ATORVASTATIN CALCIUM	10 MG
55111012105	ATORVASTATIN CALCIUM	10 MG
55111012190	ATORVASTATIN CALCIUM	10 MG
55175532503	LIPITOR	10 MG
55175532509	LIPITOR	10 MG
55289087030	LIPITOR	10 MG
55700047790	ATORVASTATIN CALCIUM	10 MG
55700065930	ATORVASTATIN CALCIUM	10 MG
55887062430	LIPITOR	10 MG
55887062460	LIPITOR	10 MG
55887062482	LIPITOR	10 MG
55887062490	LIPITOR	10 MG
57866861501	LIPITOR	10 MG
58864060830	LIPITOR	10 MG
59762015501	ATORVASTATIN CALCIUM	10 MG
59762015502	ATORVASTATIN CALCIUM	10 MG
60429032301	ATORVASTATIN CALCIUM	10 MG
60429032310	ATORVASTATIN CALCIUM	10 MG
60429032377	ATORVASTATIN CALCIUM	10 MG
60429032390	ATORVASTATIN CALCIUM	10 MG
60505257808	ATORVASTATIN CALCIUM	10 MG
60505257809	ATORVASTATIN CALCIUM	10 MG
60760035330	ATORVASTATIN CALCIUM	10 MG
60760035390	ATORVASTATIN CALCIUM	10 MG
60760090330	ATORVASTATIN CALCIUM	10 MG
60760090390	ATORVASTATIN CALCIUM	10 MG
61919054030	ATORVASTATIN CALCIUM	10 MG
61919095630	ATORVASTATIN CALCIUM	10 MG

© 2023 Davis JW et al. *JAMA Network Open.*

eTable 1. List of Statin Exposures with NDC and Dose

List of Statin Exposures

NDC	Name	Dose
**61919095690**	ATORVASTATIN CALCIUM	10 MG
**62175089043**	ATORVASTATIN CALCIUM	10 MG
**62175089046**	ATORVASTATIN CALCIUM	10 MG
**63304082705**	ATORVASTATIN CALCIUM	10 MG
**63304082790**	ATORVASTATIN CALCIUM	10 MG
**63629144601**	LIPITOR	10 MG
**63629144602**	LIPITOR	10 MG
**66105011309**	LIPITOR	10 MG
**66116027630**	LIPITOR	10 MG
**67801030103**	LIPITOR	10 MG
**67877051110**	ATORVASTATIN CALCIUM	10 MG
**67877051190**	ATORVASTATIN CALCIUM	10 MG
**68071039930**	LIPITOR	10 MG
**68071091430**	ATORVASTATIN CALCIUM	10 MG
**68084009701**	ATORVASTATIN CALCIUM	10 MG
**68084009711**	ATORVASTATIN CALCIUM	10 MG
**68084056401**	ATORVASTATIN CALCIUM	10 MG
**68115083630**	LIPITOR	10 MG
**68115083690**	LIPITOR	10 MG
**68258600003**	LIPITOR	10 MG
**68258600009**	LIPITOR	10 MG
**68382024910**	ATORVASTATIN CALCIUM	10 MG
**68382024916**	ATORVASTATIN CALCIUM	10 MG
**68645040270**	ATORVASTATIN CALCIUM	10 MG
**68645045854**	ATORVASTATIN CALCIUM	10 MG
**68645045870**	ATORVASTATIN CALCIUM	10 MG
**68645048054**	ATORVASTATIN CALCIUM	10 MG
**68645048070**	ATORVASTATIN CALCIUM	10 MG
**69097089705**	ATORVASTATIN CALCIUM	10 MG
**69097089715**	ATORVASTATIN CALCIUM	10 MG
**69097094405**	ATORVASTATIN CALCIUM	10 MG
**69097094415**	ATORVASTATIN CALCIUM	10 MG
**70377002711**	ATORVASTATIN CALCIUM	10 MG
**70377002713**	ATORVASTATIN CALCIUM	10 MG
**70882010630**	ATORVASTATIN CALCIUM	10 MG
**70882011930**	ATORVASTATIN CALCIUM	10 MG
**70934007030**	ATORVASTATIN CALCIUM	10 MG
**71205024630**	ATORVASTATIN CALCIUM	10 MG
**71205024690**	ATORVASTATIN CALCIUM	10 MG

© 2023 Davis JW et al. *JAMA Network Open.*

eTable 1. List of Statin Exposures with NDC and Dose

List of Statin Exposures

NDC	Name	Dose
**71335016901**	ATORVASTATIN CALCIUM	10 MG
**71335016902**	ATORVASTATIN CALCIUM	10 MG
**71335016903**	ATORVASTATIN CALCIUM	10 MG
**71335016904**	ATORVASTATIN CALCIUM	10 MG
**71399051001**	ATORVASTATIN CALCIUM	10 MG
**72205002205**	ATORVASTATIN CALCIUM	10 MG
**72205002290**	ATORVASTATIN CALCIUM	10 MG
**76519107503**	ATORVASTATIN CALCIUM	10 MG
**00006073061**	MEVACOR	10 MG
**00093092606**	LOVASTATIN	10 MG
**00093092610**	LOVASTATIN	10 MG
**00093092619**	LOVASTATIN	10 MG
**00093092693**	LOVASTATIN	10 MG
**00185007001**	LOVASTATIN	10 MG
**00185007005**	LOVASTATIN	10 MG
**00185007010**	LOVASTATIN	10 MG
**00185007060**	LOVASTATIN	10 MG
**00228263306**	LOVASTATIN	10 MG
**00228263350**	LOVASTATIN	10 MG
**00378651091**	LOVASTATIN	10 MG
**00781132305**	LOVASTATIN	10 MG
**00781132360**	LOVASTATIN	10 MG
**00904558152**	LOVASTATIN	10 MG
**10544023590**	LOVASTATIN	10 MG
**21695053430**	LOVASTATIN	10 MG
**23490583802**	LOVASTATIN	10 MG
**23490583806**	LOVASTATIN	10 MG
**23490583809**	LOVASTATIN	10 MG
**33261054702**	LOVASTATIN	10 MG
**33261054730**	LOVASTATIN	10 MG
**33261054760**	LOVASTATIN	10 MG
**33261054790**	LOVASTATIN	10 MG
**33358022330**	LOVASTATIN	10 MG
**42254010630**	LOVASTATIN	10 MG
**42254010690**	LOVASTATIN	10 MG
**42291037590**	LOVASTATIN	10 MG
**43063049330**	LOVASTATIN	10 MG
**43063073190**	LOVASTATIN	10 MG
**45963063301**	LOVASTATIN	10 MG

© 2023 Davis JW et al. *JAMA Network Open.*

eTable 1. List of Statin Exposures with NDC and Dose

List of Statin Exposures

NDC	Name	Dose
**45963063304**	LOVASTATIN	10 MG
**49884075401**	LOVASTATIN	10 MG
**49884075402**	LOVASTATIN	10 MG
**49884075410**	LOVASTATIN	10 MG
**49999029330**	LOVASTATIN	10 MG
**49999029360**	LOVASTATIN	10 MG
**49999029390**	LOVASTATIN	10 MG
**50090256300**	LOVASTATIN	10 MG
**50090256301**	LOVASTATIN	10 MG
**50090326800**	LOVASTATIN	10 MG
**50090326801**	LOVASTATIN	10 MG
**50090339601**	LOVASTATIN	10 MG
**50268051011**	LOVASTATIN AVPAK	10 MG
**50268051015**	LOVASTATIN AVPAK	10 MG
**51079097401**	LOVASTATIN	10 MG
**51079097420**	LOVASTATIN	10 MG
**51655001326**	LOVASTATIN	10 MG
**52959097400**	LOVASTATIN	10 MG
**52959097430**	LOVASTATIN	10 MG
**53217030402**	LOVASTATIN	10 MG
**53217030430**	LOVASTATIN	10 MG
**53217030490**	LOVASTATIN	10 MG
**53489060701**	LOVASTATIN	10 MG
**53489060706**	LOVASTATIN	10 MG
**54458084616**	LOVASTATIN	10 MG
**54458091610**	LOVASTATIN	10 MG
**54458093810**	LOVASTATIN	10 MG
**54458093816**	LOVASTATIN	10 MG
**54458098410**	LOVASTATIN	10 MG
**54569458400**	MEVACOR	10 MG
**54569534500**	LOVASTATIN	10 MG
**54569534501**	LOVASTATIN	10 MG
**54868196800**	MEVACOR	10 MG
**54868459300**	LOVASTATIN	10 MG
**54868459301**	LOVASTATIN	10 MG
**54868459302**	LOVASTATIN	10 MG
**55887035030**	LOVASTATIN	10 MG
**57866640001**	LOVASTATIN	10 MG
**58016097900**	LOVASTATIN	10 MG

© 2023 Davis JW et al. *JAMA Network Open.*

eTable 1. List of Statin Exposures with NDC and Dose

List of Statin Exposures

NDC	Name	Dose
**58016097902**	LOVASTATIN	10 MG
**58016097920**	LOVASTATIN	10 MG
**58016097930**	LOVASTATIN	10 MG
**58016097960**	LOVASTATIN	10 MG
**58016097990**	LOVASTATIN	10 MG
**58864078130**	LOVASTATIN	10 MG
**60429024810**	LOVASTATIN	10 MG
**60429024860**	LOVASTATIN	10 MG
**60429040010**	LOVASTATIN	10 MG
**60429040060**	LOVASTATIN	10 MG
**60429040090**	LOVASTATIN	10 MG
**60505017700**	LOVASTATIN	10 MG
**60760037130**	LOVASTATIN	10 MG
**61442014101**	LOVASTATIN	10 MG
**61442014110**	LOVASTATIN	10 MG
**61442014160**	LOVASTATIN	10 MG
**61919031190**	LOVASTATIN	10 MG
**62022062730**	ALTOPREV	10 MG
**62037079101**	LOVASTATIN	10 MG
**62037079160**	LOVASTATIN	10 MG
**63187081430**	LOVASTATIN	10 MG
**63187081490**	LOVASTATIN	10 MG
**63629358301**	LOVASTATIN	10 MG
**63629358302**	LOVASTATIN	10 MG
**63629358303**	LOVASTATIN	10 MG
**63739028010**	LOVASTATIN	10 MG
**63739028015**	LOVASTATIN	10 MG
**66336060205**	LOVASTATIN	10 MG
**66336060230**	LOVASTATIN	10 MG
**66336060290**	LOVASTATIN	10 MG
**68001021300**	LOVASTATIN	10 MG
**68001021306**	LOVASTATIN	10 MG
**68001021308**	LOVASTATIN	10 MG
**68001031400**	LOVASTATIN	10 MG
**68001031408**	LOVASTATIN	10 MG
**68084013101**	LOVASTATIN	10 MG
**68084055801**	LOVASTATIN	10 MG
**68084055811**	LOVASTATIN	10 MG
**68115021830**	LOVASTATIN	10 MG

© 2023 Davis JW et al. *JAMA Network Open.*

eTable 1. List of Statin Exposures with NDC and Dose

List of Statin Exposures

NDC	Name	Dose
**68180046701**	LOVASTATIN	10 MG
**68180046703**	LOVASTATIN	10 MG
**68180046707**	LOVASTATIN	10 MG
**68645057690**	LOVASTATIN	10 MG
**00003015450**	PRAVACHOL	10 MG
**00003015451**	PRAVACHOL	10 MG
**00003515405**	PRAVACHOL	10 MG
**00003515406**	PRAVACHOL	10 MG
**00093077110**	PRAVASTATIN SODIUM	10 MG
**00093077198**	PRAVASTATIN SODIUM	10 MG
**00378055277**	PRAVASTATIN SODIUM	10 MG
**00378821010**	PRAVASTATIN SODIUM	10 MG
**00378821077**	PRAVASTATIN SODIUM	10 MG
**00591001310**	PRAVASTATIN SODIUM	10 MG
**00591001319**	PRAVASTATIN SODIUM	10 MG
**00781523110**	PRAVASTATIN SODIUM	10 MG
**00781523192**	PRAVASTATIN SODIUM	10 MG
**00904589161**	PRAVASTATIN SODIUM	10 MG
**00904611361**	PRAVASTATIN SODIUM	10 MG
**10544044030**	PRAVASTATIN SODIUM	10 MG
**12280003890**	PRAVACHOL	10 MG
**16252052690**	PRAVASTATIN SODIUM	10 MG
**16729000815**	PRAVASTATIN SODIUM	10 MG
**16729000816**	PRAVASTATIN SODIUM	10 MG
**21695017830**	PRAVASTATIN SODIUM	10 MG
**23490935003**	PRAVASTATIN SODIUM	10 MG
**23490935006**	PRAVASTATIN SODIUM	10 MG
**23490935009**	PRAVASTATIN SODIUM	10 MG
**35356092130**	PRAVASTATIN SODIUM	10 MG
**42254042430**	PRAVASTATIN SODIUM	10 MG
**42291066510**	PRAVASTATIN SODIUM	10 MG
**42291066590**	PRAVASTATIN SODIUM	10 MG
**42549070830**	PRAVASTATIN SODIUM	10 MG
**49884017609**	PRAVASTATIN SODIUM	10 MG
**49884017610**	PRAVASTATIN SODIUM	10 MG
**50090159400**	PRAVASTATIN SODIUM	10 MG
**50090159401**	PRAVASTATIN SODIUM	10 MG
**50090325800**	PRAVASTATIN SODIUM	10 MG
**50090325801**	PRAVASTATIN SODIUM	10 MG

© 2023 Davis JW et al. *JAMA Network Open.*

eTable 1. List of Statin Exposures with NDC and Dose

List of Statin Exposures

NDC	Name	Dose
**50111076117**	PRAVASTATIN SODIUM	10 MG
**50268067211**	PRAVASTATIN SODIUM AVPAK	10 MG
**50268067215**	PRAVASTATIN SODIUM AVPAK	10 MG
**53217020330**	PRAVASTATIN SODIUM	10 MG
**53217020390**	PRAVASTATIN SODIUM	10 MG
**54458092710**	PRAVASTATIN SODIUM	10 MG
**54458092712**	PRAVASTATIN SODIUM	10 MG
**54458092716**	PRAVASTATIN SODIUM	10 MG
**54458098709**	PRAVASTATIN SODIUM	10 MG
**54569384000**	PRAVACHOL	10 MG
**54569434600**	PRAVACHOL	10 MG
**54569434601**	PRAVACHOL	10 MG
**54569642800**	PRAVASTATIN SODIUM	10 MG
**54569642801**	PRAVASTATIN SODIUM	10 MG
**54569859800**	PRAVACHOL	10 MG
**54868228701**	PRAVACHOL	10 MG
**54868228702**	PRAVACHOL	10 MG
**54868557600**	PRAVASTATIN SODIUM	10 MG
**54868557601**	PRAVASTATIN SODIUM	10 MG
**55111022905**	PRAVASTATIN SODIUM	10 MG
**55111022990**	PRAVASTATIN SODIUM	10 MG
**55289010430**	PRAVACHOL	10 MG
**57237016405**	PRAVASTATIN SODIUM	10 MG
**57237016490**	PRAVASTATIN SODIUM	10 MG
**58864065330**	PRAVACHOL	10 MG
**60429036705**	PRAVASTATIN SODIUM	10 MG
**60429036745**	PRAVASTATIN SODIUM	10 MG
**60429036790**	PRAVASTATIN SODIUM	10 MG
**60505016805**	PRAVASTATIN SODIUM	10 MG
**60505016809**	PRAVASTATIN SODIUM	10 MG
**60687016901**	PRAVASTATIN SODIUM	10 MG
**60687016911**	PRAVASTATIN SODIUM	10 MG
**63304059590**	PRAVASTATIN SODIUM	10 MG
**63629458801**	PRAVASTATIN SODIUM	10 MG
**66105012001**	PRAVACHOL	10 MG
**66105012003**	PRAVACHOL	10 MG
**66105012006**	PRAVACHOL	10 MG
**66105012009**	PRAVACHOL	10 MG
**66105012015**	PRAVACHOL	10 MG

© 2023 Davis JW et al. *JAMA Network Open.*

eTable 1. List of Statin Exposures with NDC and Dose

List of Statin Exposures

NDC	Name	Dose
**66105515405**	PRAVACHOL	10 MG
**68084018601**	PRAVASTATIN SODIUM	10 MG
**68084050001**	PRAVASTATIN SODIUM	10 MG
**68084050011**	PRAVASTATIN SODIUM	10 MG
**68180048502**	PRAVASTATIN SODIUM	10 MG
**68180048509**	PRAVASTATIN SODIUM	10 MG
**68258604903**	PRAVASTATIN SODIUM	10 MG
**68382007005**	PRAVASTATIN SODIUM	10 MG
**68382007016**	PRAVASTATIN SODIUM	10 MG
**68462019505**	PRAVASTATIN SODIUM	10 MG
**68462019590**	PRAVASTATIN SODIUM	10 MG
**68788741303**	PRAVASTATIN SODIUM	10 MG
**68788741306**	PRAVASTATIN SODIUM	10 MG
**68788741309**	PRAVASTATIN SODIUM	10 MG
**71335005601**	PRAVASTATIN SODIUM	10 MG
**00093757198**	ROSUVASTATIN CALCIUM	10 MG
**00310075139**	CRESTOR	10 MG
**00310075190**	CRESTOR	10 MG
**00378220377**	ROSUVASTATIN CALCIUM	10 MG
**00781540192**	ROSUVASTATIN CALCIUM	10 MG
**00904660361**	ROSUVASTATIN CALCIUM	10 MG
**00904677961**	ROSUVASTATIN CALCIUM	10 MG
**12280016415**	CRESTOR	10 MG
**12280016490**	CRESTOR	10 MG
**13668018030**	ROSUVASTATIN CALCIUM	10 MG
**13668018090**	ROSUVASTATIN CALCIUM	10 MG
**16252061630**	ROSUVASTATIN CALCIUM	10 MG
**16252061650**	ROSUVASTATIN CALCIUM	10 MG
**16252061690**	ROSUVASTATIN CALCIUM	10 MG
**16590041130**	CRESTOR	10 MG
**16729028515**	ROSUVASTATIN CALCIUM	10 MG
**16729028517**	ROSUVASTATIN CALCIUM	10 MG
**21695028790**	CRESTOR	10 MG
**27808015601**	ROSUVASTATIN CALCIUM	10 MG
**31722088390**	ROSUVASTATIN CALCIUM	10 MG
**42291074390**	ROSUVASTATIN CALCIUM	10 MG
**42292003001**	ROSUVASTATIN CALCIUM	10 MG
**42292003020**	ROSUVASTATIN CALCIUM	10 MG
**47335058381**	ROSUVASTATIN CALCIUM	10 MG

© 2023 Davis JW et al. *JAMA Network Open.*

eTable 1. List of Statin Exposures with NDC and Dose

List of Statin Exposures

NDC	Name	Dose
**47335098583**	EZALLOR SPRINKLE	10 MG
**47463009630**	CRESTOR	10 MG
**49884026109**	ROSUVASTATIN CALCIUM	10 MG
**49999087330**	CRESTOR	10 MG
**49999087390**	CRESTOR	10 MG
**50090245101**	ROSUVASTATIN CALCIUM	10 MG
**50090272300**	ROSUVASTATIN CALCIUM	10 MG
**50090272301**	ROSUVASTATIN CALCIUM	10 MG
**50090317700**	ROSUVASTATIN CALCIUM	10 MG
**50090317701**	ROSUVASTATIN CALCIUM	10 MG
**50268070911**	ROSUVASTATIN CALCIUM AVPAK	10 MG
**50268070915**	ROSUVASTATIN CALCIUM AVPAK	10 MG
**51407015490**	ROSUVASTATIN CALCIUM	10 MG
**53217029530**	ROSUVASTATIN CALCIUM	10 MG
**53217029590**	ROSUVASTATIN CALCIUM	10 MG
**54569560000**	CRESTOR	10 MG
**54569560001**	CRESTOR	10 MG
**54569667400**	ROSUVASTATIN CALCIUM	10 MG
**54569667401**	ROSUVASTATIN CALCIUM	10 MG
**54868496300**	CRESTOR	10 MG
**54868496301**	CRESTOR	10 MG
**54868496302**	CRESTOR	10 MG
**54868496303**	CRESTOR	10 MG
**55048009630**	CRESTOR	10 MG
**55289093530**	CRESTOR	10 MG
**55700051618**	ROSUVASTATIN CALCIUM	10 MG
**55700057430**	ROSUVASTATIN CALCIUM	10 MG
**57237016990**	ROSUVASTATIN CALCIUM	10 MG
**57237016999**	ROSUVASTATIN CALCIUM	10 MG
**58016003700**	CRESTOR	10 MG
**58016003730**	CRESTOR	10 MG
**58016003760**	CRESTOR	10 MG
**58016003790**	CRESTOR	10 MG
**60429084390**	ROSUVASTATIN CALCIUM	10 MG
**60505450309**	ROSUVASTATIN CALCIUM	10 MG
**60687024501**	ROSUVASTATIN CALCIUM	10 MG
**60687024511**	ROSUVASTATIN CALCIUM	10 MG
**63187086430**	ROSUVASTATIN CALCIUM	10 MG
**63187086490**	ROSUVASTATIN CALCIUM	10 MG

© 2023 Davis JW et al. *JAMA Network Open.*

eTable 1. List of Statin Exposures with NDC and Dose

List of Statin Exposures

NDC	Name	Dose
**63629338101**	CRESTOR	10 MG
**63629338102**	CRESTOR	10 MG
**63629338103**	CRESTOR	10 MG
**63629338104**	CRESTOR	10 MG
**65862029490**	ROSUVASTATIN CALCIUM	10 MG
**66105098803**	CRESTOR	10 MG
**67877044005**	ROSUVASTATIN CALCIUM	10 MG
**67877044090**	ROSUVASTATIN CALCIUM	10 MG
**68071043330**	CRESTOR	10 MG
**68258601603**	CRESTOR	10 MG
**68462026290**	ROSUVASTATIN CALCIUM	10 MG
**70377000712**	ROSUVASTATIN CALCIUM	10 MG
**70377000713**	ROSUVASTATIN CALCIUM	10 MG
**71205000830**	ROSUVASTATIN CALCIUM	10 MG
**71205005230**	ROSUVASTATIN CALCIUM	10 MG
**71205005290**	ROSUVASTATIN CALCIUM	10 MG
**71335060601**	ROSUVASTATIN CALCIUM	10 MG
**71335060602**	ROSUVASTATIN CALCIUM	10 MG
**72205000390**	ROSUVASTATIN CALCIUM	10 MG
**72205000399**	ROSUVASTATIN CALCIUM	10 MG
**76519116209**	ROSUVASTATIN CALCIUM	10 MG
**00006073528**	ZOCOR	10 MG
**00006073531**	ZOCOR	10 MG
**00006073554**	ZOCOR	10 MG
**00006073561**	ZOCOR	10 MG
**00006073582**	ZOCOR	10 MG
**00006073587**	ZOCOR	10 MG
**00093715310**	SIMVASTATIN	10 MG
**00093715319**	SIMVASTATIN	10 MG
**00093715331**	SIMVASTATIN	10 MG
**00093715356**	SIMVASTATIN	10 MG
**00093715393**	SIMVASTATIN	10 MG
**00093715398**	SIMVASTATIN	10 MG
**00406206603**	SIMVASTATIN	10 MG
**00406206605**	SIMVASTATIN	10 MG
**00406206610**	SIMVASTATIN	10 MG
**00406206660**	SIMVASTATIN	10 MG
**00406206690**	SIMVASTATIN	10 MG
**00781507131**	SIMVASTATIN	10 MG

© 2023 Davis JW et al. *JAMA Network Open.*

eTable 1. List of Statin Exposures with NDC and Dose

List of Statin Exposures

NDC	Name	Dose
00781507192	SIMVASTATIN	10 MG
00904580061	SIMVASTATIN	10 MG
13411016201	ZOCOR	10 MG
13411016203	ZOCOR	10 MG
13411016206	ZOCOR	10 MG
13411016209	ZOCOR	10 MG
13411016215	ZOCOR	10 MG
16252050630	SIMVASTATIN	10 MG
16252050650	SIMVASTATIN	10 MG
16252050690	SIMVASTATIN	10 MG
16714068201	SIMVASTATIN	10 MG
16714068202	SIMVASTATIN	10 MG
16714068203	SIMVASTATIN	10 MG
16729000410	SIMVASTATIN	10 MG
16729000415	SIMVASTATIN	10 MG
16729000417	SIMVASTATIN	10 MG
21695073930	SIMVASTATIN	10 MG
21695073990	SIMVASTATIN	10 MG
23490935303	SIMVASTATIN	10 MG
23490935306	SIMVASTATIN	10 MG
23490935309	SIMVASTATIN	10 MG
24658021110	SIMVASTATIN	10 MG
24658021130	SIMVASTATIN	10 MG
24658021145	SIMVASTATIN	10 MG
24658021190	SIMVASTATIN	10 MG
24658030110	SIMVASTATIN	10 MG
24658030115	SIMVASTATIN	10 MG
24658030130	SIMVASTATIN	10 MG
24658030145	SIMVASTATIN	10 MG
24658030190	SIMVASTATIN	10 MG
31722051110	SIMVASTATIN	10 MG
31722051190	SIMVASTATIN	10 MG
33261054600	SIMVASTATIN	10 MG
33261054602	SIMVASTATIN	10 MG
33261054630	SIMVASTATIN	10 MG
33261054660	SIMVASTATIN	10 MG
33261054690	SIMVASTATIN	10 MG
35356060430	SIMVASTATIN	10 MG
42254012930	SIMVASTATIN	10 MG

© 2023 Davis JW et al. *JAMA Network Open.*

eTable 1. List of Statin Exposures with NDC and Dose

List of Statin Exposures

NDC	Name	Dose
**42254012990**	SIMVASTATIN	10 MG
**42549071590**	SIMVASTATIN	10 MG
**42571001005**	SIMVASTATIN	10 MG
**42571001090**	SIMVASTATIN	10 MG
**43063016230**	SIMVASTATIN	10 MG
**43063072730**	SIMVASTATIN	10 MG
**43063072790**	SIMVASTATIN	10 MG
**45802009301**	SIMVASTATIN	10 MG
**45802009365**	SIMVASTATIN	10 MG
**45802009375**	SIMVASTATIN	10 MG
**50090127700**	SIMVASTATIN	10 MG
**50090127701**	SIMVASTATIN	10 MG
**50268071311**	SIMVASTATIN AVPAK	10 MG
**50268071315**	SIMVASTATIN AVPAK	10 MG
**50742013710**	SIMVASTATIN	10 MG
**51079045401**	SIMVASTATIN	10 MG
**51079045420**	SIMVASTATIN	10 MG
**51079068601**	SIMVASTATIN	10 MG
**51079068620**	SIMVASTATIN	10 MG
**52343002299**	SIMVASTATIN	10 MG
**52959098830**	SIMVASTATIN	10 MG
**54458090010**	SIMVASTATIN	10 MG
**54458093410**	SIMVASTATIN	10 MG
**54458093416**	SIMVASTATIN	10 MG
**54569418000**	ZOCOR	10 MG
**54569418001**	ZOCOR	10 MG
**54569630200**	SIMVASTATIN	10 MG
**54569630201**	SIMVASTATIN	10 MG
**54868263900**	ZOCOR	10 MG
**54868263901**	ZOCOR	10 MG
**54868562700**	SIMVASTATIN	10 MG
**54868562701**	SIMVASTATIN	10 MG
**55045365508**	SIMVASTATIN	10 MG
**55111019805**	SIMVASTATIN	10 MG
**55111019830**	SIMVASTATIN	10 MG
**55111019890**	SIMVASTATIN	10 MG
**55111073510**	SIMVASTATIN	10 MG
**55111073530**	SIMVASTATIN	10 MG
**55111073590**	SIMVASTATIN	10 MG

© 2023 Davis JW et al. *JAMA Network Open.*

eTable 1. List of Statin Exposures with NDC and Dose

List of Statin Exposures

NDC	Name	Dose
**55289033814**	SIMVASTATIN	10 MG
**55289033830**	SIMVASTATIN	10 MG
**55289033890**	SIMVASTATIN	10 MG
**55700022330**	SIMVASTATIN	10 MG
**55700051590**	SIMVASTATIN	10 MG
**55887086130**	SIMVASTATIN	10 MG
**55887086160**	SIMVASTATIN	10 MG
**55887086190**	SIMVASTATIN	10 MG
**57866798601**	ZOCOR	10 MG
**58016000800**	SIMVASTATIN	10 MG
**58016000830**	SIMVASTATIN	10 MG
**58016000860**	SIMVASTATIN	10 MG
**58016000890**	SIMVASTATIN	10 MG
**58016036400**	ZOCOR	10 MG
**58016036430**	ZOCOR	10 MG
**58016036460**	ZOCOR	10 MG
**58016036490**	ZOCOR	10 MG
**60760037930**	SIMVASTATIN	10 MG
**60760037990**	SIMVASTATIN	10 MG
**63304079010**	SIMVASTATIN	10 MG
**63304079030**	SIMVASTATIN	10 MG
**63304079090**	SIMVASTATIN	10 MG
**63739042010**	SIMVASTATIN	10 MG
**63739043610**	SIMVASTATIN	10 MG
**63739057110**	SIMVASTATIN	10 MG
**65862005126**	SIMVASTATIN	10 MG
**65862005130**	SIMVASTATIN	10 MG
**65862005190**	SIMVASTATIN	10 MG
**65862005199**	SIMVASTATIN	10 MG
**66267126001**	SIMVASTATIN	10 MG
**68071071630**	SIMVASTATIN	10 MG
**68071171109**	SIMVASTATIN	10 MG
**68084016201**	SIMVASTATIN	10 MG
**68084051101**	SIMVASTATIN	10 MG
**68084051111**	SIMVASTATIN	10 MG
**68115072030**	ZOCOR	10 MG
**68180047801**	SIMVASTATIN	10 MG
**68180047802**	SIMVASTATIN	10 MG
**68180047803**	SIMVASTATIN	10 MG

© 2023 Davis JW et al. *JAMA Network Open.*

eTable 1. List of Statin Exposures with NDC and Dose

List of Statin Exposures

NDC	Name	Dose
**68258600903**	SIMVASTATIN	10 MG
**68258600909**	SIMVASTATIN	10 MG
**68382006605**	SIMVASTATIN	10 MG
**68382006606**	SIMVASTATIN	10 MG
**68382006610**	SIMVASTATIN	10 MG
**68382006614**	SIMVASTATIN	10 MG
**68382006616**	SIMVASTATIN	10 MG
**68382006624**	SIMVASTATIN	10 MG
**70377000212**	SIMVASTATIN	10 MG
**70377000214**	SIMVASTATIN	10 MG
**70377000215**	SIMVASTATIN	10 MG
**00069216030**	CADUET	10 MG-10 MG
**00378451705**	AMLODIPINE BESYLATE-ATORVASTATIN CA	10 MG-10 MG
**00378451793**	AMLODIPINE BESYLATE-ATORVASTATIN CA	10 MG-10 MG
**00378616805**	AMLODIPINE BESYLATE-ATORVASTATIN CA	10 MG-10 MG
**00378616877**	AMLODIPINE BESYLATE-ATORVASTATIN CA	10 MG-10 MG
**00378616893**	AMLODIPINE BESYLATE-ATORVASTATIN CA	10 MG-10 MG
**12280039730**	CADUET	10 MG-10 MG
**43598032130**	AMLODIPINE BESYLATE-ATORVASTATIN CA	10 MG-10 MG
**43598032190**	AMLODIPINE BESYLATE-ATORVASTATIN CA	10 MG-10 MG
**54569588100**	CADUET	10 MG-10 MG
**54868556700**	CADUET	10 MG-10 MG
**59762673001**	AMLODIPINE BESYLATE-ATORVASTATIN CA	10 MG-10 MG
**59762673005**	AMLODIPINE BESYLATE-ATORVASTATIN CA	10 MG-10 MG
**59762673007**	AMLODIPINE BESYLATE-ATORVASTATIN CA	10 MG-10 MG
**63304059030**	AMLODIPINE BESYLATE-ATORVASTATIN CA	10 MG-10 MG
**66582032030**	LIPTRUZET	10 MG-10 MG
**66582032054**	LIPTRUZET	10 MG-10 MG
**00115138503**	EZETIMIBE-SIMVASTATIN	10 MG-10 MG
**00115138508**	EZETIMIBE-SIMVASTATIN	10 MG-10 MG
**00115138510**	EZETIMIBE-SIMVASTATIN	10 MG-10 MG
**12280038630**	VYTORIN	10 MG-10 MG
**43598058310**	EZETIMIBE-SIMVASTATIN	10 MG-10 MG

© 2023 Davis JW et al. *JAMA Network Open.*

eTable 1. List of Statin Exposures with NDC and Dose

List of Statin Exposures

NDC	Name	Dose
**43598058330**	EZETIMIBE-SIMVASTATIN	10 MG-10 MG
**43598058390**	EZETIMIBE-SIMVASTATIN	10 MG-10 MG
**43598074210**	EZETIMIBE-SIMVASTATIN	10 MG-10 MG
**43598074230**	EZETIMIBE-SIMVASTATIN	10 MG-10 MG
**43598074290**	EZETIMIBE-SIMVASTATIN	10 MG-10 MG
**45963056508**	EZETIMIBE-SIMVASTATIN	10 MG-10 MG
**45963056530**	EZETIMIBE-SIMVASTATIN	10 MG-10 MG
**51407019010**	EZETIMIBE-SIMVASTATIN	10 MG-10 MG
**51407019030**	EZETIMIBE-SIMVASTATIN	10 MG-10 MG
**51407019090**	EZETIMIBE-SIMVASTATIN	10 MG-10 MG
**54569576800**	VYTORIN	10 MG-10 MG
**54868525000**	VYTORIN	10 MG-10 MG
**60429087910**	EZETIMIBE-SIMVASTATIN	10 MG-10 MG
**60429087930**	EZETIMIBE-SIMVASTATIN	10 MG-10 MG
**60429087990**	EZETIMIBE-SIMVASTATIN	10 MG-10 MG
**62559070030**	EZETIMIBE-SIMVASTATIN	10 MG-10 MG
**62559070090**	EZETIMIBE-SIMVASTATIN	10 MG-10 MG
**66582031128**	VYTORIN	10 MG-10 MG
**66582031131**	VYTORIN	10 MG-10 MG
**66582031154**	VYTORIN	10 MG-10 MG
**66582031182**	VYTORIN	10 MG-10 MG
**67877050730**	EZETIMIBE-SIMVASTATIN	10 MG-10 MG
**67877050790**	EZETIMIBE-SIMVASTATIN	10 MG-10 MG
**69238115503**	EZETIMIBE-SIMVASTATIN	10 MG-10 MG
**69238115509**	EZETIMIBE-SIMVASTATIN	10 MG-10 MG
**00006075331**	JUVISYNC	10 MG-100 MG
**00006075354**	JUVISYNC	10 MG-100 MG
**00006075382**	JUVISYNC	10 MG-100 MG
**00069218030**	CADUET	10 MG-20 MG
**00378451805**	AMLODIPINE BESYLATE-ATORVASTATIN CA	10 MG-20 MG
**00378451893**	AMLODIPINE BESYLATE-ATORVASTATIN CA	10 MG-20 MG
**00378616905**	AMLODIPINE BESYLATE-ATORVASTATIN CA	10 MG-20 MG
**00378616977**	AMLODIPINE BESYLATE-ATORVASTATIN CA	10 MG-20 MG
**00378616993**	AMLODIPINE BESYLATE-ATORVASTATIN CA	10 MG-20 MG
**12280039830**	CADUET	10 MG-20 MG
**43598031830**	AMLODIPINE BESYLATE-ATORVASTATIN CA	10 MG-20 MG

© 2023 Davis JW et al. *JAMA Network Open.*

eTable 1. List of Statin Exposures with NDC and Dose

List of Statin Exposures

NDC	Name	Dose
**43598031890**	AMLODIPINE BESYLATE-ATORVASTATIN CA	10 MG-20 MG
**54569595100**	CADUET	10 MG-20 MG
**54868520900**	CADUET	10 MG-20 MG
**54868520901**	CADUET	10 MG-20 MG
**59762673101**	AMLODIPINE BESYLATE-ATORVASTATIN CA	10 MG-20 MG
**59762673105**	AMLODIPINE BESYLATE-ATORVASTATIN CA	10 MG-20 MG
**59762673107**	AMLODIPINE BESYLATE-ATORVASTATIN CA	10 MG-20 MG
**63304059130**	AMLODIPINE BESYLATE-ATORVASTATIN CA	10 MG-20 MG
**00115138603**	EZETIMIBE-SIMVASTATIN	10 MG-20 MG
**00115138608**	EZETIMIBE-SIMVASTATIN	10 MG-20 MG
**00115138610**	EZETIMIBE-SIMVASTATIN	10 MG-20 MG
**12280038530**	VYTORIN	10 MG-20 MG
**12280038590**	VYTORIN	10 MG-20 MG
**21695032530**	VYTORIN	10 MG-20 MG
**43598058410**	EZETIMIBE-SIMVASTATIN	10 MG-20 MG
**43598058430**	EZETIMIBE-SIMVASTATIN	10 MG-20 MG
**43598058490**	EZETIMIBE-SIMVASTATIN	10 MG-20 MG
**43598074410**	EZETIMIBE-SIMVASTATIN	10 MG-20 MG
**43598074430**	EZETIMIBE-SIMVASTATIN	10 MG-20 MG
**43598074490**	EZETIMIBE-SIMVASTATIN	10 MG-20 MG
**45963056608**	EZETIMIBE-SIMVASTATIN	10 MG-20 MG
**45963056630**	EZETIMIBE-SIMVASTATIN	10 MG-20 MG
**49999095730**	VYTORIN	10 MG-20 MG
**51407019110**	EZETIMIBE-SIMVASTATIN	10 MG-20 MG
**51407019130**	EZETIMIBE-SIMVASTATIN	10 MG-20 MG
**51407019190**	EZETIMIBE-SIMVASTATIN	10 MG-20 MG
**54569576600**	VYTORIN	10 MG-20 MG
**54868518700**	VYTORIN	10 MG-20 MG
**54868518701**	VYTORIN	10 MG-20 MG
**54868518702**	VYTORIN	10 MG-20 MG
**55048082130**	VYTORIN	10 MG-20 MG
**55289098021**	VYTORIN	10 MG-20 MG
**55887088230**	VYTORIN	10 MG-20 MG
**60429088010**	EZETIMIBE-SIMVASTATIN	10 MG-20 MG
**60429088030**	EZETIMIBE-SIMVASTATIN	10 MG-20 MG
**60429088090**	EZETIMIBE-SIMVASTATIN	10 MG-20 MG

© 2023 Davis JW et al. *JAMA Network Open.*

eTable 1. List of Statin Exposures with NDC and Dose

List of Statin Exposures

NDC	Name	Dose
**62559070130**	EZETIMIBE-SIMVASTATIN	10 MG-20 MG
**62559070190**	EZETIMIBE-SIMVASTATIN	10 MG-20 MG
**66582031228**	VYTORIN	10 MG-20 MG
**66582031231**	VYTORIN	10 MG-20 MG
**66582031254**	VYTORIN	10 MG-20 MG
**66582031282**	VYTORIN	10 MG-20 MG
**66582031287**	VYTORIN	10 MG-20 MG
**67877050830**	EZETIMIBE-SIMVASTATIN	10 MG-20 MG
**67877050890**	EZETIMIBE-SIMVASTATIN	10 MG-20 MG
**68258697003**	VYTORIN	10 MG-20 MG
**69238115603**	EZETIMIBE-SIMVASTATIN	10 MG-20 MG
**69238115609**	EZETIMIBE-SIMVASTATIN	10 MG-20 MG
**00069225030**	CADUET	10 MG-40 MG
**00378451905**	AMLODIPINE BESYLATE-ATORVASTATIN CA	10 MG-40 MG
**00378451993**	AMLODIPINE BESYLATE-ATORVASTATIN CA	10 MG-40 MG
**00378617005**	AMLODIPINE BESYLATE-ATORVASTATIN CA	10 MG-40 MG
**00378617077**	AMLODIPINE BESYLATE-ATORVASTATIN CA	10 MG-40 MG
**00378617093**	AMLODIPINE BESYLATE-ATORVASTATIN CA	10 MG-40 MG
**43598031530**	AMLODIPINE BESYLATE-ATORVASTATIN CA	10 MG-40 MG
**43598031590**	AMLODIPINE BESYLATE-ATORVASTATIN CA	10 MG-40 MG
**54569609900**	CADUET	10 MG-40 MG
**54868520000**	CADUET	10 MG-40 MG
**54868520001**	CADUET	10 MG-40 MG
**59762673201**	AMLODIPINE BESYLATE-ATORVASTATIN CA	10 MG-40 MG
**59762673205**	AMLODIPINE BESYLATE-ATORVASTATIN CA	10 MG-40 MG
**59762673207**	AMLODIPINE BESYLATE-ATORVASTATIN CA	10 MG-40 MG
**63304050030**	AMLODIPINE BESYLATE-ATORVASTATIN CA	10 MG-40 MG
**00115138702**	EZETIMIBE-SIMVASTATIN	10 MG-40 MG
**00115138708**	EZETIMIBE-SIMVASTATIN	10 MG-40 MG
**00115138710**	EZETIMIBE-SIMVASTATIN	10 MG-40 MG
**12280018130**	VYTORIN	10 MG-40 MG
**12280018190**	VYTORIN	10 MG-40 MG
**21695033930**	VYTORIN	10 MG-40 MG

© 2023 Davis JW et al. *JAMA Network Open.*

eTable 1. List of Statin Exposures with NDC and Dose

List of Statin Exposures

NDC	Name	Dose
**43598058510**	EZETIMIBE-SIMVASTATIN	10 MG-40 MG
**43598058530**	EZETIMIBE-SIMVASTATIN	10 MG-40 MG
**43598058590**	EZETIMIBE-SIMVASTATIN	10 MG-40 MG
**43598074305**	EZETIMIBE-SIMVASTATIN	10 MG-40 MG
**43598074330**	EZETIMIBE-SIMVASTATIN	10 MG-40 MG
**43598074390**	EZETIMIBE-SIMVASTATIN	10 MG-40 MG
**45963056708**	EZETIMIBE-SIMVASTATIN	10 MG-40 MG
**45963056730**	EZETIMIBE-SIMVASTATIN	10 MG-40 MG
**49999095830**	VYTORIN	10 MG-40 MG
**51407019205**	EZETIMIBE-SIMVASTATIN	10 MG-40 MG
**51407019230**	EZETIMIBE-SIMVASTATIN	10 MG-40 MG
**51407019290**	EZETIMIBE-SIMVASTATIN	10 MG-40 MG
**54569564800**	VYTORIN	10 MG-40 MG
**54868518900**	VYTORIN	10 MG-40 MG
**54868518901**	VYTORIN	10 MG-40 MG
**55048082230**	VYTORIN	10 MG-40 MG
**55289028030**	VYTORIN	10 MG-40 MG
**55887033330**	VYTORIN	10 MG-40 MG
**60429088105**	EZETIMIBE-SIMVASTATIN	10 MG-40 MG
**60429088110**	EZETIMIBE-SIMVASTATIN	10 MG-40 MG
**60429088130**	EZETIMIBE-SIMVASTATIN	10 MG-40 MG
**60429088190**	EZETIMIBE-SIMVASTATIN	10 MG-40 MG
**62559070230**	EZETIMIBE-SIMVASTATIN	10 MG-40 MG
**62559070290**	EZETIMIBE-SIMVASTATIN	10 MG-40 MG
**66582031331**	VYTORIN	10 MG-40 MG
**66582031352**	VYTORIN	10 MG-40 MG
**66582031354**	VYTORIN	10 MG-40 MG
**66582031374**	VYTORIN	10 MG-40 MG
**66582031386**	VYTORIN	10 MG-40 MG
**67877050930**	EZETIMIBE-SIMVASTATIN	10 MG-40 MG
**67877050990**	EZETIMIBE-SIMVASTATIN	10 MG-40 MG
**68258698403**	VYTORIN	10 MG-40 MG
**69238115703**	EZETIMIBE-SIMVASTATIN	10 MG-40 MG
**69238115709**	EZETIMIBE-SIMVASTATIN	10 MG-40 MG
**00006053331**	JUVISYNC	10 MG-50 MG
**00006053354**	JUVISYNC	10 MG-50 MG
**00069227030**	CADUET	10 MG-80 MG
**00378452093**	AMLODIPINE BESYLATE-ATORVASTATIN CA	10 MG-80 MG

© 2023 Davis JW et al. *JAMA Network Open.*

eTable 1. List of Statin Exposures with NDC and Dose

List of Statin Exposures

NDC	Name	Dose
**00378617177**	AMLODIPINE BESYLATE-ATORVASTATIN CA	10 MG-80 MG
**00378617193**	AMLODIPINE BESYLATE-ATORVASTATIN CA	10 MG-80 MG
**43598031330**	AMLODIPINE BESYLATE-ATORVASTATIN CA	10 MG-80 MG
**54868552300**	CADUET	10 MG-80 MG
**54868552301**	CADUET	10 MG-80 MG
**54868633500**	AMLODIPINE BESYLATE-ATORVASTATIN CA	10 MG-80 MG
**59762673301**	AMLODIPINE BESYLATE-ATORVASTATIN CA	10 MG-80 MG
**63304060330**	AMLODIPINE BESYLATE-ATORVASTATIN CA	10 MG-80 MG
**00115138802**	EZETIMIBE-SIMVASTATIN	10 MG-80 MG
**00115138808**	EZETIMIBE-SIMVASTATIN	10 MG-80 MG
**00115138810**	EZETIMIBE-SIMVASTATIN	10 MG-80 MG
**21695082730**	VYTORIN	10 MG-80 MG
**43598058610**	EZETIMIBE-SIMVASTATIN	10 MG-80 MG
**43598058630**	EZETIMIBE-SIMVASTATIN	10 MG-80 MG
**43598058690**	EZETIMIBE-SIMVASTATIN	10 MG-80 MG
**43598074530**	EZETIMIBE-SIMVASTATIN	10 MG-80 MG
**43598074590**	EZETIMIBE-SIMVASTATIN	10 MG-80 MG
**45963056808**	EZETIMIBE-SIMVASTATIN	10 MG-80 MG
**45963056830**	EZETIMIBE-SIMVASTATIN	10 MG-80 MG
**51407019305**	EZETIMIBE-SIMVASTATIN	10 MG-80 MG
**51407019330**	EZETIMIBE-SIMVASTATIN	10 MG-80 MG
**51407019390**	EZETIMIBE-SIMVASTATIN	10 MG-80 MG
**54868525900**	VYTORIN	10 MG-80 MG
**54868525901**	VYTORIN	10 MG-80 MG
**55048082330**	VYTORIN	10 MG-80 MG
**55289052030**	VYTORIN	10 MG-80 MG
**60429088205**	EZETIMIBE-SIMVASTATIN	10 MG-80 MG
**60429088230**	EZETIMIBE-SIMVASTATIN	10 MG-80 MG
**60429088290**	EZETIMIBE-SIMVASTATIN	10 MG-80 MG
**62559070330**	EZETIMIBE-SIMVASTATIN	10 MG-80 MG
**62559070390**	EZETIMIBE-SIMVASTATIN	10 MG-80 MG
**66582031531**	VYTORIN	10 MG-80 MG
**66582031552**	VYTORIN	10 MG-80 MG
**66582031554**	VYTORIN	10 MG-80 MG
**66582031566**	VYTORIN	10 MG-80 MG
**66582031574**	VYTORIN	10 MG-80 MG

© 2023 Davis JW et al. *JAMA Network Open.*

eTable 1. List of Statin Exposures with NDC and Dose

List of Statin Exposures

NDC	Name	Dose
**67877051030**	EZETIMIBE-SIMVASTATIN	10 MG-80 MG
**67877051090**	EZETIMIBE-SIMVASTATIN	10 MG-80 MG
**69238115803**	EZETIMIBE-SIMVASTATIN	10 MG-80 MG
**69238115809**	EZETIMIBE-SIMVASTATIN	10 MG-80 MG
**00074331690**	SIMCOR	1000 MG-20 MG
**00074345590**	SIMCOR	1000 MG-20 MG
**54868590400**	SIMCOR	1000 MG-20 MG
**54868590401**	SIMCOR	1000 MG-20 MG
**00074345790**	SIMCOR	1000 MG-40 MG
**54868616900**	SIMCOR	1000 MG-40 MG
**00002477190**	LIVALO	2 MG
**25208020109**	ZYPITAMAG	2 MG
**66869020407**	LIVALO	2 MG
**66869020490**	LIVALO	2 MG
**00069296030**	CADUET	2.5 MG-10 MG
**00378451093**	AMLODIPINE BESYLATE-ATORVASTATIN CA	2.5 MG-10 MG
**00378616177**	AMLODIPINE BESYLATE-ATORVASTATIN CA	2.5 MG-10 MG
**00378616193**	AMLODIPINE BESYLATE-ATORVASTATIN CA	2.5 MG-10 MG
**43598032330**	AMLODIPINE BESYLATE-ATORVASTATIN CA	2.5 MG-10 MG
**59762671001**	AMLODIPINE BESYLATE-ATORVASTATIN CA	2.5 MG-10 MG
**63304050130**	AMLODIPINE BESYLATE-ATORVASTATIN CA	2.5 MG-10 MG
**00069297030**	CADUET	2.5 MG-20 MG
**00378451193**	AMLODIPINE BESYLATE-ATORVASTATIN CA	2.5 MG-20 MG
**00378616277**	AMLODIPINE BESYLATE-ATORVASTATIN CA	2.5 MG-20 MG
**00378616293**	AMLODIPINE BESYLATE-ATORVASTATIN CA	2.5 MG-20 MG
**43598032030**	AMLODIPINE BESYLATE-ATORVASTATIN CA	2.5 MG-20 MG
**59762671101**	AMLODIPINE BESYLATE-ATORVASTATIN CA	2.5 MG-20 MG
**63304050230**	AMLODIPINE BESYLATE-ATORVASTATIN CA	2.5 MG-20 MG
**00069298030**	CADUET	2.5 MG-40 MG
**00378451293**	AMLODIPINE BESYLATE-ATORVASTATIN CA	2.5 MG-40 MG
**00378616377**	AMLODIPINE BESYLATE-ATORVASTATIN CA	2.5 MG-40 MG

© 2023 Davis JW et al. *JAMA Network Open.*

eTable 1. List of Statin Exposures with NDC and Dose

List of Statin Exposures

NDC	Name	Dose
**00378616393**	AMLODIPINE BESYLATE-ATORVASTATIN CA	2.5 MG-40 MG
**43598031730**	AMLODIPINE BESYLATE-ATORVASTATIN CA	2.5 MG-40 MG
**54868569900**	CADUET	2.5 MG-40 MG
**59762671201**	AMLODIPINE BESYLATE-ATORVASTATIN CA	2.5 MG-40 MG
**63304050330**	AMLODIPINE BESYLATE-ATORVASTATIN CA	2.5 MG-40 MG
**00071015623**	LIPITOR	20 MG
**00071015640**	LIPITOR	20 MG
**00071015694**	LIPITOR	20 MG
**00093505998**	ATORVASTATIN CALCIUM	20 MG
**00378201705**	ATORVASTATIN CALCIUM	20 MG
**00378201777**	ATORVASTATIN CALCIUM	20 MG
**00378395105**	ATORVASTATIN CALCIUM	20 MG
**00378395107**	ATORVASTATIN CALCIUM	20 MG
**00378395109**	ATORVASTATIN CALCIUM	20 MG
**00378395177**	ATORVASTATIN CALCIUM	20 MG
**00591377510**	ATORVASTATIN CALCIUM	20 MG
**00591377519**	ATORVASTATIN CALCIUM	20 MG
**00781538292**	ATORVASTATIN CALCIUM	20 MG
**00904629161**	ATORVASTATIN CALCIUM	20 MG
**10135065005**	ATORVASTATIN CALCIUM	20 MG
**13411011401**	LIPITOR	20 MG
**13411011403**	LIPITOR	20 MG
**13411011406**	LIPITOR	20 MG
**13411011409**	LIPITOR	20 MG
**13411011415**	LIPITOR	20 MG
**16714087501**	ATORVASTATIN CALCIUM	20 MG
**16714087502**	ATORVASTATIN CALCIUM	20 MG
**16714087503**	ATORVASTATIN CALCIUM	20 MG
**16729004517**	ATORVASTATIN CALCIUM	20 MG
**33261097200**	ATORVASTATIN CALCIUM	20 MG
**33261097230**	ATORVASTATIN CALCIUM	20 MG
**33261097260**	ATORVASTATIN CALCIUM	20 MG
**33261097290**	ATORVASTATIN CALCIUM	20 MG
**33261097299**	ATORVASTATIN CALCIUM	20 MG
**35356089418**	ATORVASTATIN CALCIUM	20 MG
**35356089430**	ATORVASTATIN CALCIUM	20 MG
**35356089490**	ATORVASTATIN CALCIUM	20 MG

© 2023 Davis JW et al. *JAMA Network Open.*

eTable 1. List of Statin Exposures with NDC and Dose

List of Statin Exposures

NDC	Name	Dose
**42254026130**	ATORVASTATIN CALCIUM	20 MG
**42254026145**	ATORVASTATIN CALCIUM	20 MG
**42254026190**	ATORVASTATIN CALCIUM	20 MG
**42254038230**	ATORVASTATIN CALCIUM	20 MG
**42254038290**	ATORVASTATIN CALCIUM	20 MG
**42291014410**	ATORVASTATIN CALCIUM	20 MG
**42291014490**	ATORVASTATIN CALCIUM	20 MG
**43063049630**	ATORVASTATIN CALCIUM	20 MG
**43063049660**	ATORVASTATIN CALCIUM	20 MG
**49999046730**	LIPITOR	20 MG
**49999046790**	LIPITOR	20 MG
**50090125700**	ATORVASTATIN CALCIUM	20 MG
**50090125701**	ATORVASTATIN CALCIUM	20 MG
**50090125800**	ATORVASTATIN CALCIUM	20 MG
**50090125801**	ATORVASTATIN CALCIUM	20 MG
**50268009411**	ATORVASTATIN CALCIUM AVPAK	20 MG
**50268009415**	ATORVASTATIN CALCIUM AVPAK	20 MG
**50436998803**	ATORVASTATIN CALCIUM	20 MG
**51079020901**	ATORVASTATIN CALCIUM	20 MG
**51079020920**	ATORVASTATIN CALCIUM	20 MG
**51079041001**	ATORVASTATIN CALCIUM	20 MG
**51079041020**	ATORVASTATIN CALCIUM	20 MG
**51407007905**	ATORVASTATIN CALCIUM	20 MG
**51407007990**	ATORVASTATIN CALCIUM	20 MG
**51655092030**	ATORVASTATIN CALCIUM	20 MG
**52959076090**	LIPITOR	20 MG
**53217020930**	ATORVASTATIN CALCIUM	20 MG
**53217020960**	ATORVASTATIN CALCIUM	20 MG
**53217020990**	ATORVASTATIN CALCIUM	20 MG
**53217020999**	ATORVASTATIN CALCIUM	20 MG
**54569446700**	LIPITOR	20 MG
**54569446701**	LIPITOR	20 MG
**54569628300**	ATORVASTATIN CALCIUM	20 MG
**54569628301**	ATORVASTATIN CALCIUM	20 MG
**54868394600**	LIPITOR	20 MG
**54868394601**	LIPITOR	20 MG
**54868394602**	LIPITOR	20 MG
**54868394603**	LIPITOR	20 MG
**54868394604**	LIPITOR	20 MG

© 2023 Davis JW et al. *JAMA Network Open.*

eTable 1. List of Statin Exposures with NDC and Dose

List of Statin Exposures

NDC	Name	Dose
**54868632000**	ATORVASTATIN CALCIUM	20 MG
**55111012205**	ATORVASTATIN CALCIUM	20 MG
**55111012290**	ATORVASTATIN CALCIUM	20 MG
**55289080030**	LIPITOR	20 MG
**55700054830**	ATORVASTATIN CALCIUM	20 MG
**55700054890**	ATORVASTATIN CALCIUM	20 MG
**55887073030**	LIPITOR	20 MG
**55887073060**	LIPITOR	20 MG
**55887073090**	LIPITOR	20 MG
**58864068530**	LIPITOR	20 MG
**59762015601**	ATORVASTATIN CALCIUM	20 MG
**59762015602**	ATORVASTATIN CALCIUM	20 MG
**60429032401**	ATORVASTATIN CALCIUM	20 MG
**60429032410**	ATORVASTATIN CALCIUM	20 MG
**60429032477**	ATORVASTATIN CALCIUM	20 MG
**60429032490**	ATORVASTATIN CALCIUM	20 MG
**60505257908**	ATORVASTATIN CALCIUM	20 MG
**60505257909**	ATORVASTATIN CALCIUM	20 MG
**60760035430**	ATORVASTATIN CALCIUM	20 MG
**60760035490**	ATORVASTATIN CALCIUM	20 MG
**60760090430**	ATORVASTATIN CALCIUM	20 MG
**61919025830**	ATORVASTATIN CALCIUM	20 MG
**61919025890**	ATORVASTATIN CALCIUM	20 MG
**61919063230**	ATORVASTATIN CALCIUM	20 MG
**61919091630**	ATORVASTATIN CALCIUM	20 MG
**62175089143**	ATORVASTATIN CALCIUM	20 MG
**62175089146**	ATORVASTATIN CALCIUM	20 MG
**63304082805**	ATORVASTATIN CALCIUM	20 MG
**63304082890**	ATORVASTATIN CALCIUM	20 MG
**63629144701**	LIPITOR	20 MG
**63629484901**	ATORVASTATIN CALCIUM	20 MG
**63629484902**	ATORVASTATIN CALCIUM	20 MG
**63629484903**	ATORVASTATIN CALCIUM	20 MG
**66105011409**	LIPITOR	20 MG
**67801040230**	LIPITOR	20 MG
**67877051210**	ATORVASTATIN CALCIUM	20 MG
**67877051290**	ATORVASTATIN CALCIUM	20 MG
**68071015430**	LIPITOR	20 MG
**68071091530**	ATORVASTATIN CALCIUM	20 MG

© 2023 Davis JW et al. *JAMA Network Open.*

eTable 1. List of Statin Exposures with NDC and Dose

List of Statin Exposures

NDC	Name	Dose
**68084009801**	ATORVASTATIN CALCIUM	20 MG
**68084009811**	ATORVASTATIN CALCIUM	20 MG
**68084056501**	ATORVASTATIN CALCIUM	20 MG
**68115049430**	LIPITOR	20 MG
**68115049460**	LIPITOR	20 MG
**68115080090**	LIPITOR	20 MG
**68258600103**	LIPITOR	20 MG
**68258600109**	LIPITOR	20 MG
**68382025010**	ATORVASTATIN CALCIUM	20 MG
**68382025016**	ATORVASTATIN CALCIUM	20 MG
**68645040370**	ATORVASTATIN CALCIUM	20 MG
**68645045954**	ATORVASTATIN CALCIUM	20 MG
**68645045970**	ATORVASTATIN CALCIUM	20 MG
**68645048154**	ATORVASTATIN CALCIUM	20 MG
**68645048170**	ATORVASTATIN CALCIUM	20 MG
**68645048254**	ATORVASTATIN CALCIUM	20 MG
**69097089805**	ATORVASTATIN CALCIUM	20 MG
**69097089812**	ATORVASTATIN CALCIUM	20 MG
**69097094505**	ATORVASTATIN CALCIUM	20 MG
**69097094512**	ATORVASTATIN CALCIUM	20 MG
**70377002811**	ATORVASTATIN CALCIUM	20 MG
**70377002813**	ATORVASTATIN CALCIUM	20 MG
**70882010230**	ATORVASTATIN CALCIUM	20 MG
**70882012030**	ATORVASTATIN CALCIUM	20 MG
**70934006930**	ATORVASTATIN CALCIUM	20 MG
**71205024730**	ATORVASTATIN CALCIUM	20 MG
**71205024790**	ATORVASTATIN CALCIUM	20 MG
**71335000401**	ATORVASTATIN CALCIUM	20 MG
**71335000402**	ATORVASTATIN CALCIUM	20 MG
**71335000403**	ATORVASTATIN CALCIUM	20 MG
**71335000404**	ATORVASTATIN CALCIUM	20 MG
**71335000405**	ATORVASTATIN CALCIUM	20 MG
**71399052005**	ATORVASTATIN CALCIUM	20 MG
**72205002305**	ATORVASTATIN CALCIUM	20 MG
**72205002390**	ATORVASTATIN CALCIUM	20 MG
**76519107809**	ATORVASTATIN CALCIUM	20 MG
**00078017605**	LESCOL	20 MG
**00078017615**	LESCOL	20 MG
**00093744201**	FLUVASTATIN	20 MG

© 2023 Davis JW et al. *JAMA Network Open.*

eTable 1. List of Statin Exposures with NDC and Dose

List of Statin Exposures

NDC	Name	Dose
**00093744256**	FLUVASTATIN	20 MG
**00378802077**	FLUVASTATIN	20 MG
**00378802093**	FLUVASTATIN	20 MG
**13411011101**	LESCOL	20 MG
**13411011102**	LESCOL	20 MG
**13411011103**	LESCOL	20 MG
**13411011106**	LESCOL	20 MG
**13411011110**	LESCOL	20 MG
**54569382100**	LESCOL	20 MG
**54569382101**	LESCOL	20 MG
**54868332900**	LESCOL	20 MG
**55175300203**	LESCOL	20 MG
**55289074060**	LESCOL	20 MG
**66105014701**	LESCOL	20 MG
**66105014703**	LESCOL	20 MG
**66105014706**	LESCOL	20 MG
**66105014709**	LESCOL	20 MG
**66105014710**	LESCOL	20 MG
**00006073128**	MEVACOR	20 MG
**00006073137**	MEVACOR	20 MG
**00006073161**	MEVACOR	20 MG
**00006073178**	MEVACOR	20 MG
**00006073182**	MEVACOR	20 MG
**00006073187**	MEVACOR	20 MG
**00006073194**	MEVACOR	20 MG
**00006073198**	MEVACOR	20 MG
**00093057606**	LOVASTATIN	20 MG
**00093057610**	LOVASTATIN	20 MG
**00093057619**	LOVASTATIN	20 MG
**00093057693**	LOVASTATIN	20 MG
**00185007201**	LOVASTATIN	20 MG
**00185007210**	LOVASTATIN	20 MG
**00185007260**	LOVASTATIN	20 MG
**00228263406**	LOVASTATIN	20 MG
**00228263450**	LOVASTATIN	20 MG
**00378652005**	LOVASTATIN	20 MG
**00378652091**	LOVASTATIN	20 MG
**00781121010**	LOVASTATIN	20 MG
**00781121060**	LOVASTATIN	20 MG

© 2023 Davis JW et al. *JAMA Network Open.*

eTable 1. List of Statin Exposures with NDC and Dose

List of Statin Exposures

NDC	Name	Dose
**00904558252**	LOVASTATIN	20 MG
**10544024130**	LOVASTATIN	20 MG
**10544024630**	LOVASTATIN	20 MG
**12280010860**	LOVASTATIN	20 MG
**16590054730**	LOVASTATIN	20 MG
**16590054760**	LOVASTATIN	20 MG
**16590054772**	LOVASTATIN	20 MG
**16590054790**	LOVASTATIN	20 MG
**21695053530**	LOVASTATIN	20 MG
**21695053590**	LOVASTATIN	20 MG
**23490583900**	LOVASTATIN	20 MG
**23490583901**	LOVASTATIN	20 MG
**33261054802**	LOVASTATIN	20 MG
**33261054830**	LOVASTATIN	20 MG
**33261054860**	LOVASTATIN	20 MG
**33261054890**	LOVASTATIN	20 MG
**33358022500**	LOVASTATIN	20 MG
**33358022530**	LOVASTATIN	20 MG
**33358022560**	LOVASTATIN	20 MG
**35356088530**	LOVASTATIN	20 MG
**35356088560**	LOVASTATIN	20 MG
**35356088590**	LOVASTATIN	20 MG
**42254002830**	LOVASTATIN	20 MG
**42254002890**	LOVASTATIN	20 MG
**42291037690**	LOVASTATIN	20 MG
**42554002830**	LOVASTATIN	20 MG
**43063069290**	LOVASTATIN	20 MG
**43063069293**	LOVASTATIN	20 MG
**43063098330**	LOVASTATIN	20 MG
**45963063401**	LOVASTATIN	20 MG
**45963063404**	LOVASTATIN	20 MG
**49884075501**	LOVASTATIN	20 MG
**49884075502**	LOVASTATIN	20 MG
**49884075510**	LOVASTATIN	20 MG
**49999047030**	LOVASTATIN	20 MG
**49999047060**	LOVASTATIN	20 MG
**49999047090**	LOVASTATIN	20 MG
**50090075900**	LOVASTATIN	20 MG
**50090075902**	LOVASTATIN	20 MG

© 2023 Davis JW et al. *JAMA Network Open.*

eTable 1. List of Statin Exposures with NDC and Dose

List of Statin Exposures

NDC	Name	Dose
**50268051111**	LOVASTATIN AVPAK	20 MG
**50268051115**	LOVASTATIN AVPAK	20 MG
**51079097501**	LOVASTATIN	20 MG
**51079097520**	LOVASTATIN	20 MG
**51079097530**	LOVASTATIN	20 MG
**51079097556**	LOVASTATIN	20 MG
**52959072030**	LOVASTATIN	20 MG
**52959072060**	LOVASTATIN	20 MG
**52959072090**	LOVASTATIN	20 MG
**53489060801**	LOVASTATIN	20 MG
**53489060806**	LOVASTATIN	20 MG
**53489060810**	LOVASTATIN	20 MG
**54458084516**	LOVASTATIN	20 MG
**54458087110**	LOVASTATIN	20 MG
**54458091510**	LOVASTATIN	20 MG
**54458093710**	LOVASTATIN	20 MG
**54458093716**	LOVASTATIN	20 MG
**54458098310**	LOVASTATIN	20 MG
**54569061300**	MEVACOR	20 MG
**54569061301**	MEVACOR	20 MG
**54569061302**	MEVACOR	20 MG
**54569061303**	MEVACOR	20 MG
**54569061304**	MEVACOR	20 MG
**54569534600**	LOVASTATIN	20 MG
**54569534602**	LOVASTATIN	20 MG
**54569801100**	MEVACOR	20 MG
**54569886600**	LOVASTATIN	20 MG
**54868068601**	MEVACOR	20 MG
**54868068602**	MEVACOR	20 MG
**54868068603**	MEVACOR	20 MG
**54868068604**	MEVACOR	20 MG
**54868458500**	LOVASTATIN	20 MG
**54868458501**	LOVASTATIN	20 MG
**54868458502**	LOVASTATIN	20 MG
**54868458503**	LOVASTATIN	20 MG
**55045301401**	LOVASTATIN	20 MG
**55045301402**	LOVASTATIN	20 MG
**55045301406**	LOVASTATIN	20 MG
**55045301408**	LOVASTATIN	20 MG

© 2023 Davis JW et al. *JAMA Network Open.*

eTable 1. List of Statin Exposures with NDC and Dose

List of Statin Exposures

NDC	Name	Dose
**55045301409**	LOVASTATIN	20 MG
**55048039530**	LOVASTATIN	20 MG
**55175504606**	MEVACOR	20 MG
**55289040030**	MEVACOR	20 MG
**55289088130**	LOVASTATIN	20 MG
**55289088190**	LOVASTATIN	20 MG
**55700024430**	LOVASTATIN	20 MG
**55700024460**	LOVASTATIN	20 MG
**55700024490**	LOVASTATIN	20 MG
**55887097430**	LOVASTATIN	20 MG
**57866660101**	LOVASTATIN	20 MG
**58016090000**	LOVASTATIN	20 MG
**58016090002**	LOVASTATIN	20 MG
**58016090030**	LOVASTATIN	20 MG
**58016090060**	LOVASTATIN	20 MG
**58016090090**	LOVASTATIN	20 MG
**58864078030**	LOVASTATIN	20 MG
**58864078060**	LOVASTATIN	20 MG
**59630062830**	ALTOPREV	20 MG
**60429024910**	LOVASTATIN	20 MG
**60429024960**	LOVASTATIN	20 MG
**60429040110**	LOVASTATIN	20 MG
**60429040160**	LOVASTATIN	20 MG
**60429040190**	LOVASTATIN	20 MG
**60505017800**	LOVASTATIN	20 MG
**60760037030**	LOVASTATIN	20 MG
**61442014201**	LOVASTATIN	20 MG
**61442014205**	LOVASTATIN	20 MG
**61442014210**	LOVASTATIN	20 MG
**61442014260**	LOVASTATIN	20 MG
**61919054730**	LOVASTATIN	20 MG
**61919054790**	LOVASTATIN	20 MG
**61919067671**	LOVASTATIN	20 MG
**62022062830**	ALTOPREV	20 MG
**62022077030**	ALTOCOR	20 MG
**62037079201**	LOVASTATIN	20 MG
**62037079260**	LOVASTATIN	20 MG
**63629146401**	LOVASTATIN	20 MG
**63629146402**	LOVASTATIN	20 MG

© 2023 Davis JW et al. *JAMA Network Open.*

eTable 1. List of Statin Exposures with NDC and Dose

List of Statin Exposures

NDC	Name	Dose
**63629146403**	LOVASTATIN	20 MG
**63739028103**	LOVASTATIN	20 MG
**63739028110**	LOVASTATIN	20 MG
**63739028115**	LOVASTATIN	20 MG
**63874036301**	LOVASTATIN	20 MG
**63874036310**	LOVASTATIN	20 MG
**63874036320**	LOVASTATIN	20 MG
**63874036330**	LOVASTATIN	20 MG
**63874036360**	LOVASTATIN	20 MG
**63874036390**	LOVASTATIN	20 MG
**66116027730**	LOVASTATIN	20 MG
**66336031005**	LOVASTATIN	20 MG
**66336031030**	LOVASTATIN	20 MG
**66336031060**	LOVASTATIN	20 MG
**66336031090**	LOVASTATIN	20 MG
**67046045030**	LOVASTATIN	20 MG
**68001022400**	LOVASTATIN	20 MG
**68001022406**	LOVASTATIN	20 MG
**68001022408**	LOVASTATIN	20 MG
**68001031500**	LOVASTATIN	20 MG
**68001031508**	LOVASTATIN	20 MG
**68084013201**	LOVASTATIN	20 MG
**68084055901**	LOVASTATIN	20 MG
**68084055911**	LOVASTATIN	20 MG
**68115021930**	LOVASTATIN	20 MG
**68115021960**	LOVASTATIN	20 MG
**68180046801**	LOVASTATIN	20 MG
**68180046803**	LOVASTATIN	20 MG
**68180046805**	LOVASTATIN	20 MG
**68180046807**	LOVASTATIN	20 MG
**68645056690**	LOVASTATIN	20 MG
**70515062830**	ALTOPREV	20 MG
**00003017850**	PRAVACHOL	20 MG
**00003017851**	PRAVACHOL	20 MG
**00003517805**	PRAVACHOL	20 MG
**00003517806**	PRAVACHOL	20 MG
**00003517875**	PRAVACHOL	20 MG
**00093720110**	PRAVASTATIN SODIUM	20 MG
**00093720198**	PRAVASTATIN SODIUM	20 MG

© 2023 Davis JW et al. *JAMA Network Open.*

eTable 1. List of Statin Exposures with NDC and Dose

List of Statin Exposures

NDC	Name	Dose
**00378055477**	PRAVASTATIN SODIUM	20 MG
**00378822010**	PRAVASTATIN SODIUM	20 MG
**00378822077**	PRAVASTATIN SODIUM	20 MG
**00591001410**	PRAVASTATIN SODIUM	20 MG
**00591001419**	PRAVASTATIN SODIUM	20 MG
**00781523210**	PRAVASTATIN SODIUM	20 MG
**00781523292**	PRAVASTATIN SODIUM	20 MG
**00904589261**	PRAVASTATIN SODIUM	20 MG
**00904611461**	PRAVASTATIN SODIUM	20 MG
**10544050530**	PRAVASTATIN SODIUM	20 MG
**13411011801**	PRAVACHOL	20 MG
**13411011802**	PRAVACHOL	20 MG
**13411011803**	PRAVACHOL	20 MG
**13411011806**	PRAVACHOL	20 MG
**13411011809**	PRAVACHOL	20 MG
**16252052750**	PRAVASTATIN SODIUM	20 MG
**16252052790**	PRAVASTATIN SODIUM	20 MG
**16729000915**	PRAVASTATIN SODIUM	20 MG
**16729000916**	PRAVASTATIN SODIUM	20 MG
**16729000917**	PRAVASTATIN SODIUM	20 MG
**21695017930**	PRAVASTATIN SODIUM	20 MG
**21695017990**	PRAVASTATIN SODIUM	20 MG
**23490935103**	PRAVASTATIN SODIUM	20 MG
**23490935106**	PRAVASTATIN SODIUM	20 MG
**23490935109**	PRAVASTATIN SODIUM	20 MG
**33261086700**	PRAVASTATIN SODIUM	20 MG
**33261086730**	PRAVASTATIN SODIUM	20 MG
**33261086760**	PRAVASTATIN SODIUM	20 MG
**33261086790**	PRAVASTATIN SODIUM	20 MG
**35356091930**	PRAVASTATIN SODIUM	20 MG
**35356091990**	PRAVASTATIN SODIUM	20 MG
**42254020230**	PRAVASTATIN SODIUM	20 MG
**42254020290**	PRAVASTATIN SODIUM	20 MG
**42254042530**	PRAVASTATIN SODIUM	20 MG
**42254042590**	PRAVASTATIN SODIUM	20 MG
**42291066710**	PRAVASTATIN SODIUM	20 MG
**42291066790**	PRAVASTATIN SODIUM	20 MG
**43063014330**	PRAVASTATIN SODIUM	20 MG
**43063044330**	PRAVASTATIN SODIUM	20 MG

© 2023 Davis JW et al. *JAMA Network Open.*

eTable 1. List of Statin Exposures with NDC and Dose

List of Statin Exposures

NDC	Name	Dose
**43063080730**	PRAVASTATIN SODIUM	20 MG
**49884017909**	PRAVASTATIN SODIUM	20 MG
**49884017910**	PRAVASTATIN SODIUM	20 MG
**50090202500**	PRAVASTATIN SODIUM	20 MG
**50090202501**	PRAVASTATIN SODIUM	20 MG
**50090254500**	PRAVASTATIN SODIUM	20 MG
**50090254501**	PRAVASTATIN SODIUM	20 MG
**50111076203**	PRAVASTATIN SODIUM	20 MG
**50111076217**	PRAVASTATIN SODIUM	20 MG
**50268067311**	PRAVASTATIN SODIUM AVPAK	20 MG
**50268067315**	PRAVASTATIN SODIUM AVPAK	20 MG
**51079045801**	PRAVASTATIN SODIUM	20 MG
**51079045820**	PRAVASTATIN SODIUM	20 MG
**51655007152**	PRAVASTATIN SODIUM	20 MG
**52959099030**	PRAVASTATIN SODIUM	20 MG
**52959099090**	PRAVASTATIN SODIUM	20 MG
**54458086910**	PRAVASTATIN SODIUM	20 MG
**54458090802**	PRAVASTATIN SODIUM	20 MG
**54458092610**	PRAVASTATIN SODIUM	20 MG
**54458092616**	PRAVASTATIN SODIUM	20 MG
**54458098610**	PRAVASTATIN SODIUM	20 MG
**54569371500**	PRAVACHOL	20 MG
**54569371501**	PRAVACHOL	20 MG
**54569371502**	PRAVACHOL	20 MG
**54569371503**	PRAVACHOL	20 MG
**54569407100**	PRAVACHOL	20 MG
**54569579300**	PRAVASTATIN SODIUM	20 MG
**54569579301**	PRAVASTATIN SODIUM	20 MG
**54569851000**	PRAVACHOL	20 MG
**54569851001**	PRAVACHOL	20 MG
**54868228800**	PRAVACHOL	20 MG
**54868228801**	PRAVACHOL	20 MG
**54868228802**	PRAVACHOL	20 MG
**54868557700**	PRAVASTATIN SODIUM	20 MG
**54868557701**	PRAVASTATIN SODIUM	20 MG
**55048059730**	PRAVASTATIN SODIUM	20 MG
**55111023005**	PRAVASTATIN SODIUM	20 MG
**55111023090**	PRAVASTATIN SODIUM	20 MG
**55175539003**	PRAVACHOL	20 MG

© 2023 Davis JW et al. *JAMA Network Open.*

eTable 1. List of Statin Exposures with NDC and Dose

List of Statin Exposures

NDC	Name	Dose
**55289087130**	PRAVACHOL	20 MG
**55887020330**	PRAVASTATIN	20 MG
**55887020390**	PRAVASTATIN	20 MG
**57237016505**	PRAVASTATIN SODIUM	20 MG
**57237016590**	PRAVASTATIN SODIUM	20 MG
**58016001300**	PRAVASTATIN	20 MG
**58016001330**	PRAVASTATIN	20 MG
**58016001360**	PRAVASTATIN	20 MG
**58016001390**	PRAVASTATIN	20 MG
**58016042500**	PRAVACHOL	20 MG
**58016042530**	PRAVACHOL	20 MG
**58016042560**	PRAVACHOL	20 MG
**58016042590**	PRAVACHOL	20 MG
**60429036805**	PRAVASTATIN SODIUM	20 MG
**60429036845**	PRAVASTATIN SODIUM	20 MG
**60429036890**	PRAVASTATIN SODIUM	20 MG
**60505016907**	PRAVASTATIN SODIUM	20 MG
**60505016909**	PRAVASTATIN SODIUM	20 MG
**60687017801**	PRAVASTATIN SODIUM	20 MG
**60687017811**	PRAVASTATIN SODIUM	20 MG
**60760007730**	PRAVASTATIN SODIUM	20 MG
**60760007790**	PRAVASTATIN SODIUM	20 MG
**60760042190**	PRAVASTATIN SODIUM	20 MG
**61919073190**	PRAVASTATIN SODIUM	20 MG
**63304059690**	PRAVASTATIN SODIUM	20 MG
**63629356301**	PRAVASTATIN SODIUM	20 MG
**63739064910**	PRAVASTATIN SODIUM	20 MG
**63739064941**	PRAVASTATIN SODIUM	20 MG
**66105012101**	PRAVACHOL	20 MG
**66105012103**	PRAVACHOL	20 MG
**66105012106**	PRAVACHOL	20 MG
**66105012109**	PRAVACHOL	20 MG
**66105012115**	PRAVACHOL	20 MG
**66116023830**	PRAVACHOL	20 MG
**66336068530**	PRAVASTATIN SODIUM	20 MG
**66336068590**	PRAVASTATIN SODIUM	20 MG
**68084018701**	PRAVASTATIN SODIUM	20 MG
**68084050101**	PRAVASTATIN SODIUM	20 MG
**68084050111**	PRAVASTATIN SODIUM	20 MG

© 2023 Davis JW et al. *JAMA Network Open.*

eTable 1. List of Statin Exposures with NDC and Dose

List of Statin Exposures

NDC	Name	Dose
**68180048602**	PRAVASTATIN SODIUM	20 MG
**68180048609**	PRAVASTATIN SODIUM	20 MG
**68382007105**	PRAVASTATIN SODIUM	20 MG
**68382007116**	PRAVASTATIN SODIUM	20 MG
**68462019605**	PRAVASTATIN SODIUM	20 MG
**68462019690**	PRAVASTATIN SODIUM	20 MG
**71205014930**	PRAVASTATIN SODIUM	20 MG
**00093757298**	ROSUVASTATIN CALCIUM	20 MG
**00310075239**	CRESTOR	20 MG
**00310075290**	CRESTOR	20 MG
**00378220477**	ROSUVASTATIN CALCIUM	20 MG
**00781540292**	ROSUVASTATIN CALCIUM	20 MG
**00904660461**	ROSUVASTATIN CALCIUM	20 MG
**00904678061**	ROSUVASTATIN CALCIUM	20 MG
**12280035130**	CRESTOR	20 MG
**12280035190**	CRESTOR	20 MG
**13668018130**	ROSUVASTATIN CALCIUM	20 MG
**13668018190**	ROSUVASTATIN CALCIUM	20 MG
**16252061730**	ROSUVASTATIN CALCIUM	20 MG
**16252061750**	ROSUVASTATIN CALCIUM	20 MG
**16252061790**	ROSUVASTATIN CALCIUM	20 MG
**16729028615**	ROSUVASTATIN CALCIUM	20 MG
**16729028617**	ROSUVASTATIN CALCIUM	20 MG
**21695028890**	CRESTOR	20 MG
**27808015701**	ROSUVASTATIN CALCIUM	20 MG
**31722088490**	ROSUVASTATIN CALCIUM	20 MG
**42291074490**	ROSUVASTATIN CALCIUM	20 MG
**42292003101**	ROSUVASTATIN CALCIUM	20 MG
**42292003120**	ROSUVASTATIN CALCIUM	20 MG
**47335058481**	ROSUVASTATIN CALCIUM	20 MG
**47335098683**	EZALLOR SPRINKLE	20 MG
**49884026209**	ROSUVASTATIN CALCIUM	20 MG
**49999099230**	CRESTOR	20 MG
**49999099290**	CRESTOR	20 MG
**50090272400**	ROSUVASTATIN CALCIUM	20 MG
**50090272401**	ROSUVASTATIN CALCIUM	20 MG
**50268071011**	ROSUVASTATIN CALCIUM AVPAK	20 MG
**50268071015**	ROSUVASTATIN CALCIUM AVPAK	20 MG
**51407015590**	ROSUVASTATIN CALCIUM	20 MG

© 2023 Davis JW et al. *JAMA Network Open.*

eTable 1. List of Statin Exposures with NDC and Dose

List of Statin Exposures

NDC	Name	Dose
**53217011330**	CRESTOR	20 MG
**53217011390**	CRESTOR	20 MG
**53217029630**	ROSUVASTATIN CALCIUM	20 MG
**53217029690**	ROSUVASTATIN CALCIUM	20 MG
**54569567200**	CRESTOR	20 MG
**54569567202**	CRESTOR	20 MG
**54569667500**	ROSUVASTATIN CALCIUM	20 MG
**54569667501**	ROSUVASTATIN CALCIUM	20 MG
**54868508500**	CRESTOR	20 MG
**54868508501**	CRESTOR	20 MG
**54868508502**	CRESTOR	20 MG
**54868508503**	CRESTOR	20 MG
**54868508504**	CRESTOR	20 MG
**55048009730**	CRESTOR	20 MG
**55289093230**	CRESTOR	20 MG
**55700057530**	ROSUVASTATIN CALCIUM	20 MG
**57237017090**	ROSUVASTATIN CALCIUM	20 MG
**57237017099**	ROSUVASTATIN CALCIUM	20 MG
**58016005200**	CRESTOR	20 MG
**58016005230**	CRESTOR	20 MG
**58016005260**	CRESTOR	20 MG
**58016005290**	CRESTOR	20 MG
**60429084490**	ROSUVASTATIN CALCIUM	20 MG
**60505450409**	ROSUVASTATIN CALCIUM	20 MG
**60687025601**	ROSUVASTATIN CALCIUM	20 MG
**60687025611**	ROSUVASTATIN CALCIUM	20 MG
**63187086530**	ROSUVASTATIN CALCIUM	20 MG
**63187086590**	ROSUVASTATIN CALCIUM	20 MG
**65862029590**	ROSUVASTATIN CALCIUM	20 MG
**66336067430**	CRESTOR	20 MG
**67877044105**	ROSUVASTATIN CALCIUM	20 MG
**67877044190**	ROSUVASTATIN CALCIUM	20 MG
**68071026330**	CRESTOR	20 MG
**68462026390**	ROSUVASTATIN CALCIUM	20 MG
**70377000812**	ROSUVASTATIN CALCIUM	20 MG
**70377000813**	ROSUVASTATIN CALCIUM	20 MG
**71205004490**	ROSUVASTATIN CALCIUM	20 MG
**71205007730**	ROSUVASTATIN CALCIUM	20 MG
**71205009990**	ROSUVASTATIN CALCIUM	20 MG

© 2023 Davis JW et al. *JAMA Network Open.*

eTable 1. List of Statin Exposures with NDC and Dose

List of Statin Exposures

NDC	Name	Dose
**71205027930**	ROSUVASTATIN CALCIUM	20 MG
**71335030201**	ROSUVASTATIN CALCIUM	20 MG
**71335030202**	ROSUVASTATIN CALCIUM	20 MG
**71335030203**	ROSUVASTATIN CALCIUM	20 MG
**72205000490**	ROSUVASTATIN CALCIUM	20 MG
**72205000499**	ROSUVASTATIN CALCIUM	20 MG
**76519114903**	ROSUVASTATIN CALCIUM	20 MG
**00006074028**	ZOCOR	20 MG
**00006074031**	ZOCOR	20 MG
**00006074054**	ZOCOR	20 MG
**00006074061**	ZOCOR	20 MG
**00006074082**	ZOCOR	20 MG
**00006074087**	ZOCOR	20 MG
**00093715410**	SIMVASTATIN	20 MG
**00093715419**	SIMVASTATIN	20 MG
**00093715431**	SIMVASTATIN	20 MG
**00093715456**	SIMVASTATIN	20 MG
**00093715493**	SIMVASTATIN	20 MG
**00093715498**	SIMVASTATIN	20 MG
**00406206703**	SIMVASTATIN	20 MG
**00406206705**	SIMVASTATIN	20 MG
**00406206710**	SIMVASTATIN	20 MG
**00406206760**	SIMVASTATIN	20 MG
**00406206790**	SIMVASTATIN	20 MG
**00781507231**	SIMVASTATIN	20 MG
**00781507292**	SIMVASTATIN	20 MG
**00904580161**	SIMVASTATIN	20 MG
**10544048630**	SIMVASTATIN	20 MG
**13411013201**	ZOCOR	20 MG
**13411013203**	ZOCOR	20 MG
**13411013206**	ZOCOR	20 MG
**13411013209**	ZOCOR	20 MG
**13411013215**	ZOCOR	20 MG
**16252050730**	SIMVASTATIN	20 MG
**16252050750**	SIMVASTATIN	20 MG
**16252050790**	SIMVASTATIN	20 MG
**16590044630**	SIMVASTATIN	20 MG
**16714068301**	SIMVASTATIN	20 MG
**16714068302**	SIMVASTATIN	20 MG

© 2023 Davis JW et al. *JAMA Network Open.*

eTable 1. List of Statin Exposures with NDC and Dose

List of Statin Exposures

NDC	Name	Dose
**16714068303**	SIMVASTATIN	20 MG
**16729000510**	SIMVASTATIN	20 MG
**16729000515**	SIMVASTATIN	20 MG
**16729000517**	SIMVASTATIN	20 MG
**21695074030**	SIMVASTATIN	20 MG
**21695074090**	SIMVASTATIN	20 MG
**23490935403**	SIMVASTATIN	20 MG
**23490935406**	SIMVASTATIN	20 MG
**23490935409**	SIMVASTATIN	20 MG
**24658021210**	SIMVASTATIN	20 MG
**24658021230**	SIMVASTATIN	20 MG
**24658021245**	SIMVASTATIN	20 MG
**24658021290**	SIMVASTATIN	20 MG
**24658030210**	SIMVASTATIN	20 MG
**24658030215**	SIMVASTATIN	20 MG
**24658030230**	SIMVASTATIN	20 MG
**24658030245**	SIMVASTATIN	20 MG
**24658030290**	SIMVASTATIN	20 MG
**31722051210**	SIMVASTATIN	20 MG
**31722051290**	SIMVASTATIN	20 MG
**33261054102**	SIMVASTATIN	20 MG
**33261054130**	SIMVASTATIN	20 MG
**33261054160**	SIMVASTATIN	20 MG
**33261054190**	SIMVASTATIN	20 MG
**35356066730**	SIMVASTATIN	20 MG
**35356078118**	SIMVASTATIN	20 MG
**35356078130**	SIMVASTATIN	20 MG
**35356078190**	SIMVASTATIN	20 MG
**42254006030**	SIMVASTATIN	20 MG
**42254006090**	SIMVASTATIN	20 MG
**42571002010**	SIMVASTATIN	20 MG
**42571002090**	SIMVASTATIN	20 MG
**43063000801**	SIMVASTATIN	20 MG
**43063000830**	SIMVASTATIN	20 MG
**43063000890**	SIMVASTATIN	20 MG
**45802038401**	SIMVASTATIN	20 MG
**45802038465**	SIMVASTATIN	20 MG
**45802038475**	SIMVASTATIN	20 MG
**45802038493**	SIMVASTATIN	20 MG

© 2023 Davis JW et al. *JAMA Network Open.*

eTable 1. List of Statin Exposures with NDC and Dose

List of Statin Exposures

NDC	Name	Dose
**45865042130**	SIMVASTATIN	20 MG
**45865042151**	SIMVASTATIN	20 MG
**45865042160**	SIMVASTATIN	20 MG
**45865042190**	SIMVASTATIN	20 MG
**49999030630**	ZOCOR	20 MG
**49999088930**	SIMVASTATIN	20 MG
**49999088960**	SIMVASTATIN	20 MG
**49999088990**	SIMVASTATIN	20 MG
**50090099901**	SIMVASTATIN	20 MG
**50090099902**	SIMVASTATIN	20 MG
**50090099903**	SIMVASTATIN	20 MG
**50268071411**	SIMVASTATIN AVPAK	20 MG
**50268071415**	SIMVASTATIN AVPAK	20 MG
**50436012202**	SIMVASTATIN	20 MG
**50742013810**	SIMVASTATIN	20 MG
**51079039301**	SIMVASTATIN	20 MG
**51079039320**	SIMVASTATIN	20 MG
**51079045501**	SIMVASTATIN	20 MG
**51079045520**	SIMVASTATIN	20 MG
**52343002390**	SIMVASTATIN	20 MG
**52343002399**	SIMVASTATIN	20 MG
**52959098930**	SIMVASTATIN	20 MG
**52959098990**	SIMVASTATIN	20 MG
**54458089910**	SIMVASTATIN	20 MG
**54458092804**	SIMVASTATIN	20 MG
**54458093310**	SIMVASTATIN	20 MG
**54458093316**	SIMVASTATIN	20 MG
**54569440300**	ZOCOR	20 MG
**54569583300**	SIMVASTATIN	20 MG
**54569583301**	SIMVASTATIN	20 MG
**54569583302**	SIMVASTATIN	20 MG
**54569583303**	SIMVASTATIN	20 MG
**54868310400**	ZOCOR	20 MG
**54868310401**	ZOCOR	20 MG
**54868562800**	SIMVASTATIN	20 MG
**54868562801**	SIMVASTATIN	20 MG
**54868562802**	SIMVASTATIN	20 MG
**55048077530**	SIMVASTATIN	20 MG
**55048077590**	SIMVASTATIN	20 MG

© 2023 Davis JW et al. *JAMA Network Open.*

eTable 1. List of Statin Exposures with NDC and Dose

List of Statin Exposures

NDC	Name	Dose
**55111019905**	SIMVASTATIN	20 MG
**55111019910**	SIMVASTATIN	20 MG
**55111019930**	SIMVASTATIN	20 MG
**55111019990**	SIMVASTATIN	20 MG
**55111074010**	SIMVASTATIN	20 MG
**55111074030**	SIMVASTATIN	20 MG
**55111074090**	SIMVASTATIN	20 MG
**55289029314**	SIMVASTATIN	20 MG
**55289029330**	SIMVASTATIN	20 MG
**55289029390**	SIMVASTATIN	20 MG
**55700002130**	SIMVASTATIN	20 MG
**55700002190**	SIMVASTATIN	20 MG
**55700017830**	SIMVASTATIN	20 MG
**55700054990**	SIMVASTATIN	20 MG
**55887032730**	SIMVASTATIN	20 MG
**55887032760**	SIMVASTATIN	20 MG
**55887032790**	SIMVASTATIN	20 MG
**57866393601**	SIMVASTATIN	20 MG
**57866798201**	ZOCOR	20 MG
**58016000700**	SIMVASTATIN	20 MG
**58016000730**	SIMVASTATIN	20 MG
**58016000760**	SIMVASTATIN	20 MG
**58016000790**	SIMVASTATIN	20 MG
**58016038500**	ZOCOR	20 MG
**58016038530**	ZOCOR	20 MG
**58016038560**	ZOCOR	20 MG
**58016038590**	ZOCOR	20 MG
**58864076030**	ZOCOR	20 MG
**60760000530**	SIMVASTATIN	20 MG
**60760000590**	SIMVASTATIN	20 MG
**61919044630**	SIMVASTATIN	20 MG
**61919044660**	SIMVASTATIN	20 MG
**61919044690**	SIMVASTATIN	20 MG
**63304079110**	SIMVASTATIN	20 MG
**63304079130**	SIMVASTATIN	20 MG
**63304079190**	SIMVASTATIN	20 MG
**63629339301**	SIMVASTATIN	20 MG
**63629339302**	SIMVASTATIN	20 MG
**63629339303**	SIMVASTATIN	20 MG

© 2023 Davis JW et al. *JAMA Network Open.*

eTable 1. List of Statin Exposures with NDC and Dose

List of Statin Exposures

NDC	Name	Dose
**63629339304**	SIMVASTATIN	20 MG
**63739042110**	SIMVASTATIN	20 MG
**63739043704**	SIMVASTATIN	20 MG
**63739043710**	SIMVASTATIN	20 MG
**63739057210**	SIMVASTATIN	20 MG
**65862005226**	SIMVASTATIN	20 MG
**65862005230**	SIMVASTATIN	20 MG
**65862005290**	SIMVASTATIN	20 MG
**65862005299**	SIMVASTATIN	20 MG
**66105050503**	ZOCOR	20 MG
**66267126101**	SIMVASTATIN	20 MG
**66336095430**	SIMVASTATIN	20 MG
**66336095490**	SIMVASTATIN	20 MG
**68071069930**	SIMVASTATIN	20 MG
**68084016301**	SIMVASTATIN	20 MG
**68084051201**	SIMVASTATIN	20 MG
**68084051211**	SIMVASTATIN	20 MG
**68115067230**	ZOCOR	20 MG
**68180047901**	SIMVASTATIN	20 MG
**68180047902**	SIMVASTATIN	20 MG
**68180047903**	SIMVASTATIN	20 MG
**68382006705**	SIMVASTATIN	20 MG
**68382006706**	SIMVASTATIN	20 MG
**68382006710**	SIMVASTATIN	20 MG
**68382006714**	SIMVASTATIN	20 MG
**68382006716**	SIMVASTATIN	20 MG
**68382006724**	SIMVASTATIN	20 MG
**68645026154**	SIMVASTATIN	20 MG
**68645047054**	SIMVASTATIN	20 MG
**70377000312**	SIMVASTATIN	20 MG
**70377000314**	SIMVASTATIN	20 MG
**70377000315**	SIMVASTATIN	20 MG
**29273040104**	FLOLIPID	20 MG/5 ML
**66582032130**	LIPTRUZET	20 MG-10 MG
**66582032154**	LIPTRUZET	20 MG-10 MG
**00006075731**	JUVISYNC	20 MG-100 MG
**00006075754**	JUVISYNC	20 MG-100 MG
**00006075782**	JUVISYNC	20 MG-100 MG
**00074300790**	ADVICOR	20 MG-1000 MG

© 2023 Davis JW et al. *JAMA Network Open.*

eTable 1. List of Statin Exposures with NDC and Dose

List of Statin Exposures

NDC	Name	Dose
**54868508700**	ADVICOR	20 MG-1000 MG
**60598000890**	ADVICOR	20 MG-1000 MG
**00006053531**	JUVISYNC	20 MG-50 MG
**00006053554**	JUVISYNC	20 MG-50 MG
**00074300590**	ADVICOR	20 MG-500 MG
**54868480700**	ADVICOR	20 MG-500 MG
**54868480701**	ADVICOR	20 MG-500 MG
**60598000690**	ADVICOR	20 MG-500 MG
**00074307290**	ADVICOR	20 MG-750 MG
**54868480702**	ADVICOR	20 MG-750 MG
**54868499900**	ADVICOR	20 MG-750 MG
**54868499901**	ADVICOR	20 MG-750 MG
**60598000790**	ADVICOR	20 MG-750 MG
**00003516911**	PRAVIGARD PAC	325 MG; 20 MG
**00003517411**	PRAVIGARD PAC	325 MG; 40 MG
**00003518411**	PRAVIGARD PAC	325 MG; 80 MG
**00002477290**	LIVALO	4 MG
**25208020209**	ZYPITAMAG	4 MG
**66869040407**	LIVALO	4 MG
**66869040490**	LIVALO	4 MG
**00071015723**	LIPITOR	40 MG
**00071015740**	LIPITOR	40 MG
**00071015773**	LIPITOR	40 MG
**00071015788**	LIPITOR	40 MG
**00093505898**	ATORVASTATIN CALCIUM	40 MG
**00378212105**	ATORVASTATIN CALCIUM	40 MG
**00378212177**	ATORVASTATIN CALCIUM	40 MG
**00378395205**	ATORVASTATIN CALCIUM	40 MG
**00378395207**	ATORVASTATIN CALCIUM	40 MG
**00378395209**	ATORVASTATIN CALCIUM	40 MG
**00378395277**	ATORVASTATIN CALCIUM	40 MG
**00591377605**	ATORVASTATIN CALCIUM	40 MG
**00591377619**	ATORVASTATIN CALCIUM	40 MG
**00781538492**	ATORVASTATIN CALCIUM	40 MG
**00904629261**	ATORVASTATIN CALCIUM	40 MG
**10135065110**	ATORVASTATIN CALCIUM	40 MG
**13411011501**	LIPITOR	40 MG
**13411011503**	LIPITOR	40 MG
**13411011506**	LIPITOR	40 MG

© 2023 Davis JW et al. *JAMA Network Open.*

eTable 1. List of Statin Exposures with NDC and Dose

List of Statin Exposures

NDC	Name	Dose
**13411011509**	LIPITOR	40 MG
**13411011515**	LIPITOR	40 MG
**16714087601**	ATORVASTATIN CALCIUM	40 MG
**16714087602**	ATORVASTATIN CALCIUM	40 MG
**16714087603**	ATORVASTATIN CALCIUM	40 MG
**16729004617**	ATORVASTATIN CALCIUM	40 MG
**21695025590**	LIPITOR	40 MG
**33261097300**	ATORVASTATIN CALCIUM	40 MG
**33261097330**	ATORVASTATIN CALCIUM	40 MG
**33261097360**	ATORVASTATIN CALCIUM	40 MG
**33261097390**	ATORVASTATIN CALCIUM	40 MG
**35356092930**	ATORVASTATIN CALCIUM	40 MG
**42254001930**	ATORVASTATIN CALCIUM	40 MG
**42254001945**	ATORVASTATIN CALCIUM	40 MG
**42254001990**	ATORVASTATIN CALCIUM	40 MG
**42254037930**	ATORVASTATIN CALCIUM	40 MG
**42254037990**	ATORVASTATIN CALCIUM	40 MG
**42291014550**	ATORVASTATIN CALCIUM	40 MG
**42291014590**	ATORVASTATIN CALCIUM	40 MG
**43063045330**	ATORVASTATIN CALCIUM	40 MG
**49999046830**	LIPITOR	40 MG
**49999046890**	LIPITOR	40 MG
**50090126000**	ATORVASTATIN CALCIUM	40 MG
**50090126001**	ATORVASTATIN CALCIUM	40 MG
**50090126100**	ATORVASTATIN CALCIUM	40 MG
**50090126101**	ATORVASTATIN CALCIUM	40 MG
**50090343800**	ATORVASTATIN CALCIUM	40 MG
**50090343801**	ATORVASTATIN CALCIUM	40 MG
**50268009511**	ATORVASTATIN CALCIUM AVPAK	40 MG
**50268009515**	ATORVASTATIN CALCIUM AVPAK	40 MG
**50436998901**	ATORVASTATIN CALCIUM	40 MG
**51079021001**	ATORVASTATIN CALCIUM	40 MG
**51079021020**	ATORVASTATIN CALCIUM	40 MG
**51079041101**	ATORVASTATIN CALCIUM	40 MG
**51079041120**	ATORVASTATIN CALCIUM	40 MG
**51407008010**	ATORVASTATIN CALCIUM	40 MG
**51407008090**	ATORVASTATIN CALCIUM	40 MG
**51655064030**	ATORVASTATIN CALCIUM	40 MG
**51655065152**	ATORVASTATIN CALCIUM	40 MG

© 2023 Davis JW et al. *JAMA Network Open.*

eTable 1. List of Statin Exposures with NDC and Dose

List of Statin Exposures

NDC	Name	Dose
**52959004630**	LIPITOR	40 MG
**54458088110**	ATORVASTATIN CALCIUM	40 MG
**54458088116**	ATORVASTATIN CALCIUM	40 MG
**54569458700**	LIPITOR	40 MG
**54569458701**	LIPITOR	40 MG
**54569628400**	ATORVASTATIN CALCIUM	40 MG
**54569628401**	ATORVASTATIN CALCIUM	40 MG
**54868422900**	LIPITOR	40 MG
**54868422901**	LIPITOR	40 MG
**54868422902**	LIPITOR	40 MG
**54868422903**	LIPITOR	40 MG
**54868632100**	ATORVASTATIN CALCIUM	40 MG
**55111012305**	ATORVASTATIN CALCIUM	40 MG
**55111012390**	ATORVASTATIN CALCIUM	40 MG
**55289086130**	LIPITOR	40 MG
**55887092990**	LIPITOR	40 MG
**58864062315**	LIPITOR	40 MG
**58864062330**	LIPITOR	40 MG
**59762015701**	ATORVASTATIN CALCIUM	40 MG
**59762015702**	ATORVASTATIN CALCIUM	40 MG
**60429032501**	ATORVASTATIN CALCIUM	40 MG
**60429032505**	ATORVASTATIN CALCIUM	40 MG
**60429032577**	ATORVASTATIN CALCIUM	40 MG
**60429032590**	ATORVASTATIN CALCIUM	40 MG
**60505258008**	ATORVASTATIN CALCIUM	40 MG
**60505258009**	ATORVASTATIN CALCIUM	40 MG
**60760035530**	ATORVASTATIN CALCIUM	40 MG
**60760035590**	ATORVASTATIN CALCIUM	40 MG
**60760070930**	ATORVASTATIN CALCIUM	40 MG
**60760090530**	ATORVASTATIN CALCIUM	40 MG
**60760090590**	ATORVASTATIN CALCIUM	40 MG
**61919030330**	ATORVASTATIN CALCIUM	40 MG
**61919030390**	ATORVASTATIN CALCIUM	40 MG
**62175089241**	ATORVASTATIN CALCIUM	40 MG
**62175089246**	ATORVASTATIN CALCIUM	40 MG
**63304082905**	ATORVASTATIN CALCIUM	40 MG
**63304082990**	ATORVASTATIN CALCIUM	40 MG
**63629486501**	ATORVASTATIN CALCIUM	40 MG
**66105011509**	LIPITOR	40 MG

© 2023 Davis JW et al. *JAMA Network Open.*

eTable 1. List of Statin Exposures with NDC and Dose

List of Statin Exposures

NDC	Name	Dose
**67801031403**	LIPITOR	40 MG
**67877051310**	ATORVASTATIN CALCIUM	40 MG
**67877051390**	ATORVASTATIN CALCIUM	40 MG
**68071031030**	LIPITOR	40 MG
**68071091630**	ATORVASTATIN CALCIUM	40 MG
**68084009901**	ATORVASTATIN CALCIUM	40 MG
**68084009911**	ATORVASTATIN CALCIUM	40 MG
**68084058901**	ATORVASTATIN CALCIUM	40 MG
**68115066815**	LIPITOR	40 MG
**68115066830**	LIPITOR	40 MG
**68115066890**	LIPITOR	40 MG
**68258600203**	LIPITOR	40 MG
**68258600209**	LIPITOR	40 MG
**68382025110**	ATORVASTATIN CALCIUM	40 MG
**68382025116**	ATORVASTATIN CALCIUM	40 MG
**68645041754**	ATORVASTATIN CALCIUM	40 MG
**68645046054**	ATORVASTATIN CALCIUM	40 MG
**68645048354**	ATORVASTATIN CALCIUM	40 MG
**68645056854**	ATORVASTATIN CALCIUM	40 MG
**69097089905**	ATORVASTATIN CALCIUM	40 MG
**69097089915**	ATORVASTATIN CALCIUM	40 MG
**69097094605**	ATORVASTATIN CALCIUM	40 MG
**69097094615**	ATORVASTATIN CALCIUM	40 MG
**70377002911**	ATORVASTATIN CALCIUM	40 MG
**70377002913**	ATORVASTATIN CALCIUM	40 MG
**70882010930**	ATORVASTATIN CALCIUM	40 MG
**70934009130**	ATORVASTATIN CALCIUM	40 MG
**71205026490**	ATORVASTATIN CALCIUM	40 MG
**71335010201**	ATORVASTATIN CALCIUM	40 MG
**71335010202**	ATORVASTATIN CALCIUM	40 MG
**71335010203**	ATORVASTATIN CALCIUM	40 MG
**71335010204**	ATORVASTATIN CALCIUM	40 MG
**71399054001**	ATORVASTATIN CALCIUM	40 MG
**72205002405**	ATORVASTATIN CALCIUM	40 MG
**72205002490**	ATORVASTATIN CALCIUM	40 MG
**76519106303**	ATORVASTATIN CALCIUM	40 MG
**00078023405**	LESCOL	40 MG
**00078023415**	LESCOL	40 MG
**00093744301**	FLUVASTATIN	40 MG

© 2023 Davis JW et al. *JAMA Network Open.*

eTable 1. List of Statin Exposures with NDC and Dose

List of Statin Exposures

NDC	Name	Dose
**00093744356**	FLUVASTATIN	40 MG
**00378802177**	FLUVASTATIN	40 MG
**00378802193**	FLUVASTATIN	40 MG
**54569476100**	LESCOL	40 MG
**54569476101**	LESCOL	40 MG
**54868422400**	LESCOL	40 MG
**54868422401**	LESCOL	40 MG
**55289047630**	LESCOL	40 MG
**00006073261**	MEVACOR	40 MG
**00006073282**	MEVACOR	40 MG
**00006073287**	MEVACOR	40 MG
**00006073294**	MEVACOR	40 MG
**00093092806**	LOVASTATIN	40 MG
**00093092810**	LOVASTATIN	40 MG
**00093092819**	LOVASTATIN	40 MG
**00093092893**	LOVASTATIN	40 MG
**00185007401**	LOVASTATIN	40 MG
**00185007410**	LOVASTATIN	40 MG
**00185007460**	LOVASTATIN	40 MG
**00228263506**	LOVASTATIN	40 MG
**00228263550**	LOVASTATIN	40 MG
**00378654005**	LOVASTATIN	40 MG
**00378654091**	LOVASTATIN	40 MG
**00781121310**	LOVASTATIN	40 MG
**00781121360**	LOVASTATIN	40 MG
**00904558352**	LOVASTATIN	40 MG
**10544024230**	LOVASTATIN	40 MG
**16590094130**	LOVASTATIN	40 MG
**21695053630**	LOVASTATIN	40 MG
**21695053690**	LOVASTATIN	40 MG
**23490584001**	LOVASTATIN	40 MG
**23490584002**	LOVASTATIN	40 MG
**33261054900**	LOVASTATIN	40 MG
**33261054902**	LOVASTATIN	40 MG
**33261054930**	LOVASTATIN	40 MG
**33261054960**	LOVASTATIN	40 MG
**33261054990**	LOVASTATIN	40 MG
**33358022630**	LOVASTATIN	40 MG
**42254002530**	LOVASTATIN	40 MG

© 2023 Davis JW et al. *JAMA Network Open.*

eTable 1. List of Statin Exposures with NDC and Dose

List of Statin Exposures

NDC	Name	Dose
**42254002590**	LOVASTATIN	40 MG
**42291037710**	LOVASTATIN	40 MG
**42291037790**	LOVASTATIN	40 MG
**43063054814**	LOVASTATIN	40 MG
**43063054830**	LOVASTATIN	40 MG
**43063054890**	LOVASTATIN	40 MG
**43063093930**	LOVASTATIN	40 MG
**45963063501**	LOVASTATIN	40 MG
**45963063504**	LOVASTATIN	40 MG
**49884075601**	LOVASTATIN	40 MG
**49884075602**	LOVASTATIN	40 MG
**49884075610**	LOVASTATIN	40 MG
**49999047100**	LOVASTATIN	40 MG
**49999047130**	LOVASTATIN	40 MG
**49999047160**	LOVASTATIN	40 MG
**49999047190**	LOVASTATIN	40 MG
**50090076200**	LOVASTATIN	40 MG
**50090076202**	LOVASTATIN	40 MG
**50090321600**	LOVASTATIN	40 MG
**50090321602**	LOVASTATIN	40 MG
**50268051211**	LOVASTATIN AVPAK	40 MG
**50268051215**	LOVASTATIN AVPAK	40 MG
**51079097601**	LOVASTATIN	40 MG
**51079097620**	LOVASTATIN	40 MG
**51079097630**	LOVASTATIN	40 MG
**51079097656**	LOVASTATIN	40 MG
**51655028124**	LOVASTATIN	40 MG
**53489060901**	LOVASTATIN	40 MG
**53489060906**	LOVASTATIN	40 MG
**53489060910**	LOVASTATIN	40 MG
**54458084416**	LOVASTATIN	40 MG
**54458087010**	LOVASTATIN	40 MG
**54458091410**	LOVASTATIN	40 MG
**54458093610**	LOVASTATIN	40 MG
**54458093616**	LOVASTATIN	40 MG
**54458098210**	LOVASTATIN	40 MG
**54569325600**	MEVACOR	40 MG
**54569325601**	MEVACOR	40 MG
**54569534700**	LOVASTATIN	40 MG

© 2023 Davis JW et al. *JAMA Network Open.*

eTable 1. List of Statin Exposures with NDC and Dose

List of Statin Exposures

NDC	Name	Dose
**54569534702**	LOVASTATIN	40 MG
**54868108700**	MEVACOR	40 MG
**54868108701**	MEVACOR	40 MG
**54868477400**	LOVASTATIN	40 MG
**54868477401**	LOVASTATIN	40 MG
**54868477402**	LOVASTATIN	40 MG
**54868477403**	LOVASTATIN	40 MG
**54868551300**	ALTOPREV	40 MG
**55045301501**	LOVASTATIN	40 MG
**55045301508**	LOVASTATIN	40 MG
**55048039430**	LOVASTATIN	40 MG
**55048039490**	LOVASTATIN	40 MG
**55289054830**	MEVACOR	40 MG
**55289069214**	LOVASTATIN	40 MG
**55289069230**	LOVASTATIN	40 MG
**55289069290**	LOVASTATIN	40 MG
**55887036930**	LOVASTATIN	40 MG
**55887036960**	LOVASTATIN	40 MG
**55887036990**	LOVASTATIN	40 MG
**57866650001**	LOVASTATIN	40 MG
**58016092200**	LOVASTATIN	40 MG
**58016092202**	LOVASTATIN	40 MG
**58016092230**	LOVASTATIN	40 MG
**58016092260**	LOVASTATIN	40 MG
**58016092290**	LOVASTATIN	40 MG
**59630062930**	ALTOPREV	40 MG
**60429025010**	LOVASTATIN	40 MG
**60429025060**	LOVASTATIN	40 MG
**60429025090**	LOVASTATIN	40 MG
**60429040210**	LOVASTATIN	40 MG
**60429040260**	LOVASTATIN	40 MG
**60429040290**	LOVASTATIN	40 MG
**60505017900**	LOVASTATIN	40 MG
**60760037230**	LOVASTATIN	40 MG
**61442014301**	LOVASTATIN	40 MG
**61442014305**	LOVASTATIN	40 MG
**61442014310**	LOVASTATIN	40 MG
**61442014360**	LOVASTATIN	40 MG
**61919094190**	LOVASTATIN	40 MG

© 2023 Davis JW et al. *JAMA Network Open.*

eTable 1. List of Statin Exposures with NDC and Dose

List of Statin Exposures

NDC	Name	Dose
**62022062930**	ALTOPREV	40 MG
**62022078030**	ALTOCOR	40 MG
**62037079301**	LOVASTATIN	40 MG
**62037079360**	LOVASTATIN	40 MG
**63629178401**	LOVASTATIN	40 MG
**63629178402**	LOVASTATIN	40 MG
**63739028203**	LOVASTATIN	40 MG
**63739028210**	LOVASTATIN	40 MG
**66267056130**	LOVASTATIN	40 MG
**66267056160**	LOVASTATIN	40 MG
**66267056190**	LOVASTATIN	40 MG
**66336041205**	LOVASTATIN	40 MG
**66336041230**	LOVASTATIN	40 MG
**66336041290**	LOVASTATIN	40 MG
**67046045130**	LOVASTATIN	40 MG
**68001021400**	LOVASTATIN	40 MG
**68001021406**	LOVASTATIN	40 MG
**68001021408**	LOVASTATIN	40 MG
**68001031600**	LOVASTATIN	40 MG
**68001031608**	LOVASTATIN	40 MG
**68084013301**	LOVASTATIN	40 MG
**68084056001**	LOVASTATIN	40 MG
**68115065800**	LOVASTATIN	40 MG
**68180046901**	LOVASTATIN	40 MG
**68180046903**	LOVASTATIN	40 MG
**68180046905**	LOVASTATIN	40 MG
**68180046907**	LOVASTATIN	40 MG
**68645056790**	LOVASTATIN	40 MG
**70515062930**	ALTOPREV	40 MG
**71205019930**	LOVASTATIN	40 MG
**71205019990**	LOVASTATIN	40 MG
**71335004501**	LOVASTATIN	40 MG
**00003019450**	PRAVACHOL	40 MG
**00003519410**	PRAVACHOL	40 MG
**00003519433**	PRAVACHOL	40 MG
**00093720210**	PRAVASTATIN SODIUM	40 MG
**00093720298**	PRAVASTATIN SODIUM	40 MG
**00378055777**	PRAVASTATIN SODIUM	40 MG
**00378824010**	PRAVASTATIN SODIUM	40 MG

© 2023 Davis JW et al. *JAMA Network Open.*

eTable 1. List of Statin Exposures with NDC and Dose

List of Statin Exposures

NDC	Name	Dose
**00378824077**	PRAVASTATIN SODIUM	40 MG
**00591001610**	PRAVASTATIN SODIUM	40 MG
**00591001619**	PRAVASTATIN SODIUM	40 MG
**00781523410**	PRAVASTATIN SODIUM	40 MG
**00781523492**	PRAVASTATIN SODIUM	40 MG
**00904589361**	PRAVASTATIN SODIUM	40 MG
**00904611561**	PRAVASTATIN SODIUM	40 MG
**10544050730**	PRAVASTATIN SODIUM	40 MG
**12280033515**	PRAVASTATIN	40 MG
**12280033530**	PRAVASTATIN	40 MG
**12280033590**	PRAVASTATIN	40 MG
**13411011901**	PRAVACHOL	40 MG
**13411011902**	PRAVACHOL	40 MG
**13411011903**	PRAVACHOL	40 MG
**13411011906**	PRAVACHOL	40 MG
**13411011909**	PRAVACHOL	40 MG
**16252052850**	PRAVASTATIN SODIUM	40 MG
**16252052890**	PRAVASTATIN SODIUM	40 MG
**16590054630**	PRAVASTATIN SODIUM	40 MG
**16590054660**	PRAVASTATIN SODIUM	40 MG
**16590054690**	PRAVASTATIN SODIUM	40 MG
**16729001015**	PRAVASTATIN SODIUM	40 MG
**16729001016**	PRAVASTATIN SODIUM	40 MG
**16729001017**	PRAVASTATIN SODIUM	40 MG
**21695018030**	PRAVASTATIN SODIUM	40 MG
**21695018090**	PRAVASTATIN SODIUM	40 MG
**23490935203**	PRAVASTATIN SODIUM	40 MG
**23490935206**	PRAVASTATIN SODIUM	40 MG
**23490935209**	PRAVASTATIN SODIUM	40 MG
**33261086800**	PRAVASTATIN SODIUM	40 MG
**33261086830**	PRAVASTATIN SODIUM	40 MG
**33261086860**	PRAVASTATIN SODIUM	40 MG
**33261086890**	PRAVASTATIN SODIUM	40 MG
**35356012530**	PRAVASTATIN SODIUM	40 MG
**42254013130**	PRAVASTATIN SODIUM	40 MG
**42254013190**	PRAVASTATIN SODIUM	40 MG
**42254043430**	PRAVASTATIN SODIUM	40 MG
**42291066810**	PRAVASTATIN SODIUM	40 MG
**42291066890**	PRAVASTATIN SODIUM	40 MG

© 2023 Davis JW et al. *JAMA Network Open.*

eTable 1. List of Statin Exposures with NDC and Dose

List of Statin Exposures

NDC	Name	Dose
**43063019530**	PRAVASTATIN SODIUM	40 MG
**43063044430**	PRAVASTATIN SODIUM	40 MG
**43063080830**	PRAVASTATIN SODIUM	40 MG
**49884018009**	PRAVASTATIN SODIUM	40 MG
**49884018010**	PRAVASTATIN SODIUM	40 MG
**50090202400**	PRAVASTATIN SODIUM	40 MG
**50090202401**	PRAVASTATIN SODIUM	40 MG
**50111076403**	PRAVASTATIN SODIUM	40 MG
**50111076417**	PRAVASTATIN SODIUM	40 MG
**51079078201**	PRAVASTATIN SODIUM	40 MG
**51079078220**	PRAVASTATIN SODIUM	40 MG
**51655007252**	PRAVASTATIN SODIUM	40 MG
**51655007352**	PRAVASTATIN SODIUM	40 MG
**54458086710**	PRAVASTATIN SODIUM	40 MG
**54458092510**	PRAVASTATIN SODIUM	40 MG
**54458092516**	PRAVASTATIN SODIUM	40 MG
**54458098510**	PRAVASTATIN SODIUM	40 MG
**54569461000**	PRAVACHOL	40 MG
**54569579400**	PRAVASTATIN SODIUM	40 MG
**54569579401**	PRAVASTATIN SODIUM	40 MG
**54868327000**	PRAVACHOL	40 MG
**54868327001**	PRAVACHOL	40 MG
**54868327002**	PRAVACHOL	40 MG
**54868557800**	PRAVASTATIN SODIUM	40 MG
**54868557801**	PRAVASTATIN SODIUM	40 MG
**54868557802**	PRAVASTATIN SODIUM	40 MG
**55048059630**	PRAVASTATIN SODIUM	40 MG
**55111023105**	PRAVASTATIN SODIUM	40 MG
**55111023190**	PRAVASTATIN SODIUM	40 MG
**55289087330**	PRAVACHOL	40 MG
**55887019290**	PRAVASTATIN	40 MG
**57237016605**	PRAVASTATIN SODIUM	40 MG
**57237016690**	PRAVASTATIN SODIUM	40 MG
**57866393201**	PRAVASTATIN	40 MG
**58016001200**	PRAVASTATIN	40 MG
**58016001230**	PRAVASTATIN	40 MG
**58016001260**	PRAVASTATIN	40 MG
**58016001290**	PRAVASTATIN	40 MG
**58864074315**	PRAVACHOL	40 MG

© 2023 Davis JW et al. *JAMA Network Open.*

eTable 1. List of Statin Exposures with NDC and Dose

List of Statin Exposures

NDC	Name	Dose
**58864074330**	PRAVACHOL	40 MG
**60429036905**	PRAVASTATIN SODIUM	40 MG
**60429036945**	PRAVASTATIN SODIUM	40 MG
**60429036990**	PRAVASTATIN SODIUM	40 MG
**60505017007**	PRAVASTATIN SODIUM	40 MG
**60505017008**	PRAVASTATIN SODIUM	40 MG
**60505017009**	PRAVASTATIN SODIUM	40 MG
**60687019001**	PRAVASTATIN SODIUM	40 MG
**60687019011**	PRAVASTATIN SODIUM	40 MG
**60760007830**	PRAVASTATIN SODIUM	40 MG
**60760007890**	PRAVASTATIN SODIUM	40 MG
**60760042290**	PRAVASTATIN SODIUM	40 MG
**60760052890**	PRAVASTATIN SODIUM	40 MG
**61919054690**	PRAVASTATIN SODIUM	40 MG
**61919070830**	PRAVASTATIN SODIUM	40 MG
**63304059790**	PRAVASTATIN SODIUM	40 MG
**63629160601**	PRAVASTATIN SODIUM	40 MG
**63629160602**	PRAVASTATIN SODIUM	40 MG
**66105012201**	PRAVACHOL	40 MG
**66105012203**	PRAVACHOL	40 MG
**66105012206**	PRAVACHOL	40 MG
**66105012209**	PRAVACHOL	40 MG
**66105012215**	PRAVACHOL	40 MG
**66336081330**	PRAVASTATIN SODIUM	40 MG
**66336081390**	PRAVASTATIN SODIUM	40 MG
**68084018801**	PRAVASTATIN SODIUM	40 MG
**68084050201**	PRAVASTATIN SODIUM	40 MG
**68084050211**	PRAVASTATIN SODIUM	40 MG
**68115066490**	PRAVACHOL	40 MG
**68180048702**	PRAVASTATIN SODIUM	40 MG
**68180048709**	PRAVASTATIN SODIUM	40 MG
**68382007205**	PRAVASTATIN SODIUM	40 MG
**68382007216**	PRAVASTATIN SODIUM	40 MG
**68462019705**	PRAVASTATIN SODIUM	40 MG
**68462019790**	PRAVASTATIN SODIUM	40 MG
**68788725301**	PRAVASTATIN SODIUM	40 MG
**68788725302**	PRAVASTATIN SODIUM	40 MG
**68788725303**	PRAVASTATIN SODIUM	40 MG
**68788725306**	PRAVASTATIN SODIUM	40 MG

© 2023 Davis JW et al. *JAMA Network Open.*

eTable 1. List of Statin Exposures with NDC and Dose

List of Statin Exposures

NDC	Name	Dose
**68788725308**	PRAVASTATIN SODIUM	40 MG
**68788725309**	PRAVASTATIN SODIUM	40 MG
**70934016130**	PRAVASTATIN SODIUM	40 MG
**71205012130**	PRAVASTATIN SODIUM	40 MG
**76519120009**	PRAVASTATIN SODIUM	40 MG
**00093757356**	ROSUVASTATIN CALCIUM	40 MG
**00310075430**	CRESTOR	40 MG
**00378223293**	ROSUVASTATIN CALCIUM	40 MG
**00781540331**	ROSUVASTATIN CALCIUM	40 MG
**00904660561**	ROSUVASTATIN CALCIUM	40 MG
**00904678161**	ROSUVASTATIN CALCIUM	40 MG
**13668018230**	ROSUVASTATIN CALCIUM	40 MG
**16252061830**	ROSUVASTATIN CALCIUM	40 MG
**16252061850**	ROSUVASTATIN CALCIUM	40 MG
**16252061890**	ROSUVASTATIN CALCIUM	40 MG
**16729028710**	ROSUVASTATIN CALCIUM	40 MG
**16729028715**	ROSUVASTATIN CALCIUM	40 MG
**16729028717**	ROSUVASTATIN CALCIUM	40 MG
**21695065930**	CRESTOR	40 MG
**27808015801**	ROSUVASTATIN CALCIUM	40 MG
**31722088530**	ROSUVASTATIN CALCIUM	40 MG
**35356041330**	CRESTOR	40 MG
**42291074590**	ROSUVASTATIN CALCIUM	40 MG
**42292003201**	ROSUVASTATIN CALCIUM	40 MG
**42292003220**	ROSUVASTATIN CALCIUM	40 MG
**47335058583**	ROSUVASTATIN CALCIUM	40 MG
**47335098783**	EZALLOR SPRINKLE	40 MG
**49884026311**	ROSUVASTATIN CALCIUM	40 MG
**50090272500**	ROSUVASTATIN CALCIUM	40 MG
**50090272501**	ROSUVASTATIN CALCIUM	40 MG
**50090343700**	ROSUVASTATIN CALCIUM	40 MG
**50090343701**	ROSUVASTATIN CALCIUM	40 MG
**50268071111**	ROSUVASTATIN CALCIUM AVPAK	40 MG
**50268071115**	ROSUVASTATIN CALCIUM AVPAK	40 MG
**51407015630**	ROSUVASTATIN CALCIUM	40 MG
**54569605401**	CRESTOR	40 MG
**54569667600**	ROSUVASTATIN CALCIUM	40 MG
**54569667601**	ROSUVASTATIN CALCIUM	40 MG
**54868189000**	CRESTOR	40 MG

© 2023 Davis JW et al. *JAMA Network Open.*

eTable 1. List of Statin Exposures with NDC and Dose

List of Statin Exposures

NDC	Name	Dose
**54868189001**	CRESTOR	40 MG
**55700053490**	ROSUVASTATIN CALCIUM	40 MG
**57237017105**	ROSUVASTATIN CALCIUM	40 MG
**57237017130**	ROSUVASTATIN CALCIUM	40 MG
**57237017190**	ROSUVASTATIN CALCIUM	40 MG
**58016007100**	CRESTOR	40 MG
**58016007130**	CRESTOR	40 MG
**58016007160**	CRESTOR	40 MG
**58016007190**	CRESTOR	40 MG
**60429084530**	ROSUVASTATIN CALCIUM	40 MG
**60505450503**	ROSUVASTATIN CALCIUM	40 MG
**63187086930**	ROSUVASTATIN CALCIUM	40 MG
**63187087230**	ROSUVASTATIN CALCIUM	40 MG
**65862029630**	ROSUVASTATIN CALCIUM	40 MG
**67877044230**	ROSUVASTATIN CALCIUM	40 MG
**67877044290**	ROSUVASTATIN CALCIUM	40 MG
**68258698303**	CRESTOR	40 MG
**68462026430**	ROSUVASTATIN CALCIUM	40 MG
**70377000911**	ROSUVASTATIN CALCIUM	40 MG
**70377000912**	ROSUVASTATIN CALCIUM	40 MG
**70377000913**	ROSUVASTATIN CALCIUM	40 MG
**71205007830**	ROSUVASTATIN CALCIUM	40 MG
**71205017630**	ROSUVASTATIN CALCIUM	40 MG
**71205017690**	ROSUVASTATIN CALCIUM	40 MG
**71335039001**	ROSUVASTATIN CALCIUM	40 MG
**72205000530**	ROSUVASTATIN CALCIUM	40 MG
**72205000590**	ROSUVASTATIN CALCIUM	40 MG
**72205000599**	ROSUVASTATIN CALCIUM	40 MG
**76519115303**	ROSUVASTATIN CALCIUM	40 MG
**00006074928**	ZOCOR	40 MG
**00006074931**	ZOCOR	40 MG
**00006074954**	ZOCOR	40 MG
**00006074961**	ZOCOR	40 MG
**00006074982**	ZOCOR	40 MG
**00093715510**	SIMVASTATIN	40 MG
**00093715519**	SIMVASTATIN	40 MG
**00093715531**	SIMVASTATIN	40 MG
**00093715556**	SIMVASTATIN	40 MG
**00093715593**	SIMVASTATIN	40 MG

© 2023 Davis JW et al. *JAMA Network Open.*

eTable 1. List of Statin Exposures with NDC and Dose

List of Statin Exposures

NDC	Name	Dose
**00093715598**	SIMVASTATIN	40 MG
**00406206803**	SIMVASTATIN	40 MG
**00406206805**	SIMVASTATIN	40 MG
**00406206810**	SIMVASTATIN	40 MG
**00406206860**	SIMVASTATIN	40 MG
**00406206890**	SIMVASTATIN	40 MG
**00781507331**	SIMVASTATIN	40 MG
**00781507392**	SIMVASTATIN	40 MG
**00904580261**	SIMVASTATIN	40 MG
**10544048790**	SIMVASTATIN	40 MG
**13411013301**	ZOCOR	40 MG
**13411013303**	ZOCOR	40 MG
**13411013306**	ZOCOR	40 MG
**13411013309**	ZOCOR	40 MG
**13411013315**	ZOCOR	40 MG
**16252050830**	SIMVASTATIN	40 MG
**16252050850**	SIMVASTATIN	40 MG
**16252050890**	SIMVASTATIN	40 MG
**16590043130**	SIMVASTATIN	40 MG
**16590043190**	SIMVASTATIN	40 MG
**16714068401**	SIMVASTATIN	40 MG
**16714068402**	SIMVASTATIN	40 MG
**16714068403**	SIMVASTATIN	40 MG
**16729000610**	SIMVASTATIN	40 MG
**16729000615**	SIMVASTATIN	40 MG
**16729000617**	SIMVASTATIN	40 MG
**21695074130**	SIMVASTATIN	40 MG
**21695074190**	SIMVASTATIN	40 MG
**23490935503**	SIMVASTATIN	40 MG
**23490935506**	SIMVASTATIN	40 MG
**23490935509**	SIMVASTATIN	40 MG
**24658021310**	SIMVASTATIN	40 MG
**24658021330**	SIMVASTATIN	40 MG
**24658021345**	SIMVASTATIN	40 MG
**24658021390**	SIMVASTATIN	40 MG
**24658030310**	SIMVASTATIN	40 MG
**24658030315**	SIMVASTATIN	40 MG
**24658030330**	SIMVASTATIN	40 MG
**24658030345**	SIMVASTATIN	40 MG

© 2023 Davis JW et al. *JAMA Network Open.*

eTable 1. List of Statin Exposures with NDC and Dose

List of Statin Exposures

NDC	Name	Dose
**24658030390**	SIMVASTATIN	40 MG
**31722051310**	SIMVASTATIN	40 MG
**31722051390**	SIMVASTATIN	40 MG
**33261054202**	SIMVASTATIN	40 MG
**33261054230**	SIMVASTATIN	40 MG
**33261054260**	SIMVASTATIN	40 MG
**33261054290**	SIMVASTATIN	40 MG
**35356077530**	SIMVASTATIN	40 MG
**35356077590**	SIMVASTATIN	40 MG
**42254003230**	SIMVASTATIN	40 MG
**42254003245**	SIMVASTATIN	40 MG
**42254003290**	SIMVASTATIN	40 MG
**42254022530**	SIMVASTATIN	40 MG
**42571004010**	SIMVASTATIN	40 MG
**42571004090**	SIMVASTATIN	40 MG
**43063059330**	SIMVASTATIN	40 MG
**43063059390**	SIMVASTATIN	40 MG
**43063072630**	SIMVASTATIN	40 MG
**43063072690**	SIMVASTATIN	40 MG
**45802087901**	SIMVASTATIN	40 MG
**45802087965**	SIMVASTATIN	40 MG
**45802087975**	SIMVASTATIN	40 MG
**45802087993**	SIMVASTATIN	40 MG
**45865046030**	SIMVASTATIN	40 MG
**45865046051**	SIMVASTATIN	40 MG
**45865046060**	SIMVASTATIN	40 MG
**45865046090**	SIMVASTATIN	40 MG
**49999048830**	ZOCOR	40 MG
**49999090315**	SIMVASTATIN	40 MG
**49999090330**	SIMVASTATIN	40 MG
**49999090390**	SIMVASTATIN	40 MG
**50090100001**	SIMVASTATIN	40 MG
**50090100002**	SIMVASTATIN	40 MG
**50090100003**	SIMVASTATIN	40 MG
**50090100004**	SIMVASTATIN	40 MG
**50268071511**	SIMVASTATIN AVPAK	40 MG
**50268071515**	SIMVASTATIN AVPAK	40 MG
**50742013910**	SIMVASTATIN	40 MG
**51079039801**	SIMVASTATIN	40 MG

© 2023 Davis JW et al. *JAMA Network Open.*

eTable 1. List of Statin Exposures with NDC and Dose

List of Statin Exposures

NDC	Name	Dose
**51079039820**	SIMVASTATIN	40 MG
**51079045601**	SIMVASTATIN	40 MG
**51079045620**	SIMVASTATIN	40 MG
**52959011230**	ZOCOR	40 MG
**52959094430**	SIMVASTATIN	40 MG
**54458089410**	SIMVASTATIN	40 MG
**54458093210**	SIMVASTATIN	40 MG
**54458093216**	SIMVASTATIN	40 MG
**54569440400**	ZOCOR	40 MG
**54569583400**	SIMVASTATIN	40 MG
**54569583401**	SIMVASTATIN	40 MG
**54569583402**	SIMVASTATIN	40 MG
**54569583403**	SIMVASTATIN	40 MG
**54569583404**	SIMVASTATIN	40 MG
**54868415700**	ZOCOR	40 MG
**54868415701**	ZOCOR	40 MG
**54868415702**	ZOCOR	40 MG
**54868562900**	SIMVASTATIN	40 MG
**54868562901**	SIMVASTATIN	40 MG
**54868562902**	SIMVASTATIN	40 MG
**54868562903**	SIMVASTATIN	40 MG
**54868562904**	SIMVASTATIN	40 MG
**55045310008**	ZOCOR	40 MG
**55048077430**	SIMVASTATIN	40 MG
**55048077490**	SIMVASTATIN	40 MG
**55111020005**	SIMVASTATIN	40 MG
**55111020010**	SIMVASTATIN	40 MG
**55111020030**	SIMVASTATIN	40 MG
**55111020090**	SIMVASTATIN	40 MG
**55111074910**	SIMVASTATIN	40 MG
**55111074930**	SIMVASTATIN	40 MG
**55111074990**	SIMVASTATIN	40 MG
**55289039530**	SIMVASTATIN	40 MG
**55289039590**	SIMVASTATIN	40 MG
**55289087430**	ZOCOR	40 MG
**55700026530**	SIMVASTATIN	40 MG
**55700026590**	SIMVASTATIN	40 MG
**55700042618**	SIMVASTATIN	40 MG
**55700042630**	SIMVASTATIN	40 MG

© 2023 Davis JW et al. *JAMA Network Open.*

eTable 1. List of Statin Exposures with NDC and Dose

List of Statin Exposures

NDC	Name	Dose
**55700042690**	SIMVASTATIN	40 MG
**55700055090**	SIMVASTATIN	40 MG
**55887085810**	SIMVASTATIN	40 MG
**55887085830**	SIMVASTATIN	40 MG
**55887085860**	SIMVASTATIN	40 MG
**55887085890**	SIMVASTATIN	40 MG
**57866394901**	SIMVASTATIN	40 MG
**57866798301**	ZOCOR	40 MG
**58016000600**	SIMVASTATIN	40 MG
**58016000630**	SIMVASTATIN	40 MG
**58016000660**	SIMVASTATIN	40 MG
**58016000690**	SIMVASTATIN	40 MG
**58016036500**	ZOCOR	40 MG
**58016036530**	ZOCOR	40 MG
**58016036560**	ZOCOR	40 MG
**58016036590**	ZOCOR	40 MG
**58864068230**	ZOCOR	40 MG
**60687021001**	SIMVASTATIN	40 MG
**60687021011**	SIMVASTATIN	40 MG
**60760000630**	SIMVASTATIN	40 MG
**60760000690**	SIMVASTATIN	40 MG
**61919043130**	SIMVASTATIN	40 MG
**61919043190**	SIMVASTATIN	40 MG
**63187044990**	SIMVASTATIN	40 MG
**63304079210**	SIMVASTATIN	40 MG
**63304079230**	SIMVASTATIN	40 MG
**63304079290**	SIMVASTATIN	40 MG
**63739042210**	SIMVASTATIN	40 MG
**63739043810**	SIMVASTATIN	40 MG
**63739057310**	SIMVASTATIN	40 MG
**65862005322**	SIMVASTATIN	40 MG
**65862005330**	SIMVASTATIN	40 MG
**65862005390**	SIMVASTATIN	40 MG
**65862005399**	SIMVASTATIN	40 MG
**66105050601**	ZOCOR	40 MG
**66105050603**	ZOCOR	40 MG
**66105050606**	ZOCOR	40 MG
**66105050609**	ZOCOR	40 MG
**66105050610**	ZOCOR	40 MG

© 2023 Davis JW et al. *JAMA Network Open.*

eTable 1. List of Statin Exposures with NDC and Dose

List of Statin Exposures

NDC	Name	Dose
**66336095330**	SIMVASTATIN	40 MG
**66336095390**	SIMVASTATIN	40 MG
**68084016401**	SIMVASTATIN	40 MG
**68084051301**	SIMVASTATIN	40 MG
**68115077730**	ZOCOR	40 MG
**68115077790**	ZOCOR	40 MG
**68180046403**	SIMVASTATIN	40 MG
**68180046406**	SIMVASTATIN	40 MG
**68180046409**	SIMVASTATIN	40 MG
**68180048001**	SIMVASTATIN	40 MG
**68180048002**	SIMVASTATIN	40 MG
**68180048003**	SIMVASTATIN	40 MG
**68382006805**	SIMVASTATIN	40 MG
**68382006806**	SIMVASTATIN	40 MG
**68382006810**	SIMVASTATIN	40 MG
**68382006814**	SIMVASTATIN	40 MG
**68382006816**	SIMVASTATIN	40 MG
**68382006840**	SIMVASTATIN	40 MG
**68645026254**	SIMVASTATIN	40 MG
**68645047154**	SIMVASTATIN	40 MG
**68645052754**	SIMVASTATIN	40 MG
**70377000412**	SIMVASTATIN	40 MG
**70377000414**	SIMVASTATIN	40 MG
**70377000415**	SIMVASTATIN	40 MG
**71205019230**	SIMVASTATIN	40 MG
**29273040204**	FLOLIPID	40 MG/5 ML
**66582032230**	LIPTRUZET	40 MG-10 MG
**66582032254**	LIPTRUZET	40 MG-10 MG
**00006077331**	JUVISYNC	40 MG-100 MG
**00006077354**	JUVISYNC	40 MG-100 MG
**00006077382**	JUVISYNC	40 MG-100 MG
**00074301090**	ADVICOR	40 MG-1000 MG
**54868565300**	ADVICOR	40 MG-1000 MG
**54868565301**	ADVICOR	40 MG-1000 MG
**60598000990**	ADVICOR	40 MG-1000 MG
**00006053731**	JUVISYNC	40 MG-50 MG
**00006053754**	JUVISYNC	40 MG-50 MG
**00093757098**	ROSUVASTATIN CALCIUM	5 MG
**00310075590**	CRESTOR	5 MG

© 2023 Davis JW et al. *JAMA Network Open.*

eTable 1. List of Statin Exposures with NDC and Dose

List of Statin Exposures

NDC	Name	Dose
**00378220177**	ROSUVASTATIN CALCIUM	5 MG
**00781540092**	ROSUVASTATIN CALCIUM	5 MG
**00904660261**	ROSUVASTATIN CALCIUM	5 MG
**00904677861**	ROSUVASTATIN CALCIUM	5 MG
**13668017930**	ROSUVASTATIN CALCIUM	5 MG
**13668017990**	ROSUVASTATIN CALCIUM	5 MG
**16252061530**	ROSUVASTATIN CALCIUM	5 MG
**16252061550**	ROSUVASTATIN CALCIUM	5 MG
**16252061590**	ROSUVASTATIN CALCIUM	5 MG
**16729028415**	ROSUVASTATIN CALCIUM	5 MG
**16729028417**	ROSUVASTATIN CALCIUM	5 MG
**21695075990**	CRESTOR	5 MG
**27808015501**	ROSUVASTATIN CALCIUM	5 MG
**31722088290**	ROSUVASTATIN CALCIUM	5 MG
**35356051930**	CRESTOR	5 MG
**42291074290**	ROSUVASTATIN CALCIUM	5 MG
**42292002901**	ROSUVASTATIN CALCIUM	5 MG
**42292002920**	ROSUVASTATIN CALCIUM	5 MG
**47335058281**	ROSUVASTATIN CALCIUM	5 MG
**47335098483**	EZALLOR SPRINKLE	5 MG
**47463009530**	CRESTOR	5 MG
**49884026009**	ROSUVASTATIN CALCIUM	5 MG
**50090272200**	ROSUVASTATIN CALCIUM	5 MG
**50090272201**	ROSUVASTATIN CALCIUM	5 MG
**50090317600**	ROSUVASTATIN CALCIUM	5 MG
**50090317601**	ROSUVASTATIN CALCIUM	5 MG
**51407015390**	ROSUVASTATIN CALCIUM	5 MG
**53217029730**	ROSUVASTATIN CALCIUM	5 MG
**53217029790**	ROSUVASTATIN CALCIUM	5 MG
**54569574600**	CRESTOR	5 MG
**54569667300**	ROSUVASTATIN CALCIUM	5 MG
**54569667301**	ROSUVASTATIN CALCIUM	5 MG
**54868534100**	CRESTOR	5 MG
**54868534101**	CRESTOR	5 MG
**55048009530**	CRESTOR	5 MG
**57237016805**	ROSUVASTATIN CALCIUM	5 MG
**57237016890**	ROSUVASTATIN CALCIUM	5 MG
**60429084290**	ROSUVASTATIN CALCIUM	5 MG
**60505450209**	ROSUVASTATIN CALCIUM	5 MG

© 2023 Davis JW et al. *JAMA Network Open.*

eTable 1. List of Statin Exposures with NDC and Dose

List of Statin Exposures

NDC	Name	Dose
**60687023401**	ROSUVASTATIN CALCIUM	5 MG
**60687023411**	ROSUVASTATIN CALCIUM	5 MG
**63187086090**	ROSUVASTATIN CALCIUM	5 MG
**63629715801**	ROSUVASTATIN CALCIUM	5 MG
**63629715802**	ROSUVASTATIN CALCIUM	5 MG
**65862029390**	ROSUVASTATIN CALCIUM	5 MG
**67877043990**	ROSUVASTATIN CALCIUM	5 MG
**68071078430**	CRESTOR	5 MG
**68258601703**	CRESTOR	5 MG
**68462026190**	ROSUVASTATIN CALCIUM	5 MG
**68788731002**	ROSUVASTATIN CALCIUM	5 MG
**68788731003**	ROSUVASTATIN CALCIUM	5 MG
**68788731006**	ROSUVASTATIN CALCIUM	5 MG
**68788731009**	ROSUVASTATIN CALCIUM	5 MG
**70377000612**	ROSUVASTATIN CALCIUM	5 MG
**70377000613**	ROSUVASTATIN CALCIUM	5 MG
**71335074901**	ROSUVASTATIN CALCIUM	5 MG
**72205000290**	ROSUVASTATIN CALCIUM	5 MG
**72205000299**	ROSUVASTATIN CALCIUM	5 MG
**00006072628**	ZOCOR	5 MG
**00006072631**	ZOCOR	5 MG
**00006072654**	ZOCOR	5 MG
**00006072661**	ZOCOR	5 MG
**00006072682**	ZOCOR	5 MG
**00093715219**	SIMVASTATIN	5 MG
**00093715256**	SIMVASTATIN	5 MG
**00093715293**	SIMVASTATIN	5 MG
**00093715298**	SIMVASTATIN	5 MG
**00406206503**	SIMVASTATIN	5 MG
**00406206505**	SIMVASTATIN	5 MG
**00406206510**	SIMVASTATIN	5 MG
**00406206560**	SIMVASTATIN	5 MG
**00406206590**	SIMVASTATIN	5 MG
**00781507031**	SIMVASTATIN	5 MG
**00781507092**	SIMVASTATIN	5 MG
**13411016101**	ZOCOR	5 MG
**13411016103**	ZOCOR	5 MG
**13411016106**	ZOCOR	5 MG
**13411016109**	ZOCOR	5 MG

© 2023 Davis JW et al. *JAMA Network Open.*

eTable 1. List of Statin Exposures with NDC and Dose

List of Statin Exposures

NDC	Name	Dose
**13411016115**	ZOCOR	5 MG
**16252050530**	SIMVASTATIN	5 MG
**16252050550**	SIMVASTATIN	5 MG
**16252050590**	SIMVASTATIN	5 MG
**16714068101**	SIMVASTATIN	5 MG
**16714068102**	SIMVASTATIN	5 MG
**16729015610**	SIMVASTATIN	5 MG
**16729015615**	SIMVASTATIN	5 MG
**16729015617**	SIMVASTATIN	5 MG
**21695073890**	SIMVASTATIN	5 MG
**23490935603**	SIMVASTATIN	5 MG
**23490935606**	SIMVASTATIN	5 MG
**23490935609**	SIMVASTATIN	5 MG
**24658021010**	SIMVASTATIN	5 MG
**24658021030**	SIMVASTATIN	5 MG
**24658021045**	SIMVASTATIN	5 MG
**24658021090**	SIMVASTATIN	5 MG
**24658030010**	SIMVASTATIN	5 MG
**24658030030**	SIMVASTATIN	5 MG
**24658030045**	SIMVASTATIN	5 MG
**24658030090**	SIMVASTATIN	5 MG
**31722051010**	SIMVASTATIN	5 MG
**31722051090**	SIMVASTATIN	5 MG
**42571000590**	SIMVASTATIN	5 MG
**45802092465**	SIMVASTATIN	5 MG
**49999090090**	SIMVASTATIN	5 MG
**50090138700**	SIMVASTATIN	5 MG
**50090254300**	SIMVASTATIN	5 MG
**50268071211**	SIMVASTATIN AVPAK	5 MG
**50268071215**	SIMVASTATIN AVPAK	5 MG
**54569645000**	SIMVASTATIN	5 MG
**54868606600**	SIMVASTATIN	5 MG
**55111019705**	SIMVASTATIN	5 MG
**55111019730**	SIMVASTATIN	5 MG
**55111019790**	SIMVASTATIN	5 MG
**55111072610**	SIMVASTATIN	5 MG
**55111072630**	SIMVASTATIN	5 MG
**55111072690**	SIMVASTATIN	5 MG
**58864073930**	ZOCOR	5 MG

© 2023 Davis JW et al. *JAMA Network Open.*

eTable 1. List of Statin Exposures with NDC and Dose

List of Statin Exposures

NDC	Name	Dose
**63304078910**	SIMVASTATIN	5 MG
**63304078930**	SIMVASTATIN	5 MG
**63304078990**	SIMVASTATIN	5 MG
**63739041910**	SIMVASTATIN	5 MG
**63739043510**	SIMVASTATIN	5 MG
**63739057010**	SIMVASTATIN	5 MG
**65862005030**	SIMVASTATIN	5 MG
**65862005090**	SIMVASTATIN	5 MG
**65862005099**	SIMVASTATIN	5 MG
**68084016101**	SIMVASTATIN	5 MG
**68084051001**	SIMVASTATIN	5 MG
**68180048206**	SIMVASTATIN	5 MG
**68180048209**	SIMVASTATIN	5 MG
**68258605003**	SIMVASTATIN	5 MG
**68258698509**	SIMVASTATIN	5 MG
**68382006505**	SIMVASTATIN	5 MG
**68382006506**	SIMVASTATIN	5 MG
**68382006510**	SIMVASTATIN	5 MG
**68382006514**	SIMVASTATIN	5 MG
**68382006516**	SIMVASTATIN	5 MG
**70377000112**	SIMVASTATIN	5 MG
**70377000114**	SIMVASTATIN	5 MG
**70377000115**	SIMVASTATIN	5 MG
**71205007090**	SIMVASTATIN	5 MG
**00069215030**	CADUET	5 MG-10 MG
**00378451305**	AMLODIPINE BESYLATE-ATORVASTATIN CA	5 MG-10 MG
**00378451393**	AMLODIPINE BESYLATE-ATORVASTATIN CA	5 MG-10 MG
**00378616405**	AMLODIPINE BESYLATE-ATORVASTATIN CA	5 MG-10 MG
**00378616477**	AMLODIPINE BESYLATE-ATORVASTATIN CA	5 MG-10 MG
**00378616493**	AMLODIPINE BESYLATE-ATORVASTATIN CA	5 MG-10 MG
**12280039930**	CADUET	5 MG-10 MG
**43598032230**	AMLODIPINE BESYLATE-ATORVASTATIN CA	5 MG-10 MG
**43598032290**	AMLODIPINE BESYLATE-ATORVASTATIN CA	5 MG-10 MG
**49999098930**	CADUET	5 MG-10 MG
**54569570400**	CADUET	5 MG-10 MG

© 2023 Davis JW et al. *JAMA Network Open.*

eTable 1. List of Statin Exposures with NDC and Dose

List of Statin Exposures

NDC	Name	Dose
**54868328700**	CADUET	5 MG-10 MG
**54868328701**	CADUET	5 MG-10 MG
**59762672001**	AMLODIPINE BESYLATE-ATORVASTATIN CA	5 MG-10 MG
**59762672005**	AMLODIPINE BESYLATE-ATORVASTATIN CA	5 MG-10 MG
**59762672007**	AMLODIPINE BESYLATE-ATORVASTATIN CA	5 MG-10 MG
**63304058730**	AMLODIPINE BESYLATE-ATORVASTATIN CA	5 MG-10 MG
**00069217030**	CADUET	5 MG-20 MG
**00378451405**	AMLODIPINE BESYLATE-ATORVASTATIN CA	5 MG-20 MG
**00378451493**	AMLODIPINE BESYLATE-ATORVASTATIN CA	5 MG-20 MG
**00378616505**	AMLODIPINE BESYLATE-ATORVASTATIN CA	5 MG-20 MG
**00378616577**	AMLODIPINE BESYLATE-ATORVASTATIN CA	5 MG-20 MG
**00378616593**	AMLODIPINE BESYLATE-ATORVASTATIN CA	5 MG-20 MG
**43598031930**	AMLODIPINE BESYLATE-ATORVASTATIN CA	5 MG-20 MG
**43598031990**	AMLODIPINE BESYLATE-ATORVASTATIN CA	5 MG-20 MG
**54868120700**	CADUET	5 MG-20 MG
**54868120701**	CADUET	5 MG-20 MG
**59762672101**	AMLODIPINE BESYLATE-ATORVASTATIN CA	5 MG-20 MG
**59762672105**	AMLODIPINE BESYLATE-ATORVASTATIN CA	5 MG-20 MG
**59762672107**	AMLODIPINE BESYLATE-ATORVASTATIN CA	5 MG-20 MG
**63304058830**	AMLODIPINE BESYLATE-ATORVASTATIN CA	5 MG-20 MG
**00069219030**	CADUET	5 MG-40 MG
**00378451505**	AMLODIPINE BESYLATE-ATORVASTATIN CA	5 MG-40 MG
**00378451593**	AMLODIPINE BESYLATE-ATORVASTATIN CA	5 MG-40 MG
**00378616605**	AMLODIPINE BESYLATE-ATORVASTATIN CA	5 MG-40 MG
**00378616677**	AMLODIPINE BESYLATE-ATORVASTATIN CA	5 MG-40 MG
**00378616693**	AMLODIPINE BESYLATE-ATORVASTATIN CA	5 MG-40 MG
**43598031630**	AMLODIPINE BESYLATE-ATORVASTATIN CA	5 MG-40 MG

© 2023 Davis JW et al. *JAMA Network Open.*

eTable 1. List of Statin Exposures with NDC and Dose

List of Statin Exposures

NDC	Name	Dose
**43598031690**	AMLODIPINE BESYLATE-ATORVASTATIN CA	5 MG-40 MG
**54868517900**	CADUET	5 MG-40 MG
**59762672201**	AMLODIPINE BESYLATE-ATORVASTATIN CA	5 MG-40 MG
**59762672205**	AMLODIPINE BESYLATE-ATORVASTATIN CA	5 MG-40 MG
**59762672207**	AMLODIPINE BESYLATE-ATORVASTATIN CA	5 MG-40 MG
**63304058930**	AMLODIPINE BESYLATE-ATORVASTATIN CA	5 MG-40 MG
**00069226030**	CADUET	5 MG-80 MG
**00378451693**	AMLODIPINE BESYLATE-ATORVASTATIN CA	5 MG-80 MG
**00378616777**	AMLODIPINE BESYLATE-ATORVASTATIN CA	5 MG-80 MG
**00378616793**	AMLODIPINE BESYLATE-ATORVASTATIN CA	5 MG-80 MG
**43598031430**	AMLODIPINE BESYLATE-ATORVASTATIN CA	5 MG-80 MG
**54868542000**	CADUET	5 MG-80 MG
**59762672301**	AMLODIPINE BESYLATE-ATORVASTATIN CA	5 MG-80 MG
**63304049930**	AMLODIPINE BESYLATE-ATORVASTATIN CA	5 MG-80 MG
**00074331290**	SIMCOR	500 MG-20 MG
**54868588600**	SIMCOR	500 MG-20 MG
**54868588601**	SIMCOR	500 MG-20 MG
**00074345903**	SIMCOR	500 MG-40 MG
**00074345990**	SIMCOR	500 MG-40 MG
**54868535800**	ALTOPREV	60 MG
**59630063030**	ALTOPREV	60 MG
**62022063030**	ALTOPREV	60 MG
**62022078130**	ALTOCOR	60 MG
**70515063030**	ALTOPREV	60 MG
**00074331590**	SIMCOR	750 MG-20 MG
**54868590700**	SIMCOR	750 MG-20 MG
**54868590701**	SIMCOR	750 MG-20 MG
**00071015823**	LIPITOR	80 MG
**00071015873**	LIPITOR	80 MG
**00071015888**	LIPITOR	80 MG
**00071015892**	LIPITOR	80 MG
**00093505798**	ATORVASTATIN CALCIUM	80 MG
**00378212205**	ATORVASTATIN CALCIUM	80 MG

© 2023 Davis JW et al. *JAMA Network Open.*

eTable 1. List of Statin Exposures with NDC and Dose

List of Statin Exposures

NDC	Name	Dose
**00378212277**	ATORVASTATIN CALCIUM	80 MG
**00378395305**	ATORVASTATIN CALCIUM	80 MG
**00378395307**	ATORVASTATIN CALCIUM	80 MG
**00378395309**	ATORVASTATIN CALCIUM	80 MG
**00378395377**	ATORVASTATIN CALCIUM	80 MG
**00591377705**	ATORVASTATIN CALCIUM	80 MG
**00591377719**	ATORVASTATIN CALCIUM	80 MG
**00781538892**	ATORVASTATIN CALCIUM	80 MG
**00904629304**	ATORVASTATIN CALCIUM	80 MG
**10135065305**	ATORVASTATIN CALCIUM	80 MG
**12280015030**	LIPITOR	80 MG
**16714087701**	ATORVASTATIN CALCIUM	80 MG
**16714087702**	ATORVASTATIN CALCIUM	80 MG
**16714087703**	ATORVASTATIN CALCIUM	80 MG
**16729004716**	ATORVASTATIN CALCIUM	80 MG
**33261099530**	ATORVASTATIN CALCIUM	80 MG
**33261099560**	ATORVASTATIN CALCIUM	80 MG
**33261099590**	ATORVASTATIN CALCIUM	80 MG
**42254026730**	ATORVASTATIN CALCIUM	80 MG
**42254026745**	ATORVASTATIN CALCIUM	80 MG
**42254026790**	ATORVASTATIN CALCIUM	80 MG
**42254039290**	ATORVASTATIN CALCIUM	80 MG
**42291014650**	ATORVASTATIN CALCIUM	80 MG
**42291014690**	ATORVASTATIN CALCIUM	80 MG
**49999088230**	LIPITOR	80 MG
**49999088290**	LIPITOR	80 MG
**50090126400**	ATORVASTATIN CALCIUM	80 MG
**50090126401**	ATORVASTATIN CALCIUM	80 MG
**50090126500**	ATORVASTATIN CALCIUM	80 MG
**50090126501**	ATORVASTATIN CALCIUM	80 MG
**50268009611**	ATORVASTATIN CALCIUM AVPAK	80 MG
**50268009612**	ATORVASTATIN CALCIUM AVPAK	80 MG
**51079021101**	ATORVASTATIN CALCIUM	80 MG
**51079021103**	ATORVASTATIN CALCIUM	80 MG
**51079041201**	ATORVASTATIN CALCIUM	80 MG
**51079041203**	ATORVASTATIN CALCIUM	80 MG
**51407008105**	ATORVASTATIN CALCIUM	80 MG
**51407008190**	ATORVASTATIN CALCIUM	80 MG
**51655088030**	ATORVASTATIN CALCIUM	80 MG

© 2023 Davis JW et al. *JAMA Network Open.*

eTable 1. List of Statin Exposures with NDC and Dose

List of Statin Exposures

NDC	Name	Dose
**53217031930**	ATORVASTATIN CALCIUM	80 MG
**53217031990**	ATORVASTATIN CALCIUM	80 MG
**54569538200**	LIPITOR	80 MG
**54569628500**	ATORVASTATIN CALCIUM	80 MG
**54569628501**	ATORVASTATIN CALCIUM	80 MG
**54868493400**	LIPITOR	80 MG
**54868493401**	LIPITOR	80 MG
**54868493402**	LIPITOR	80 MG
**54868493403**	LIPITOR	80 MG
**54868632200**	ATORVASTATIN CALCIUM	80 MG
**55111012405**	ATORVASTATIN CALCIUM	80 MG
**55111012490**	ATORVASTATIN CALCIUM	80 MG
**55700003430**	ATORVASTATIN CALCIUM	80 MG
**58016005100**	LIPITOR	80 MG
**58016005130**	LIPITOR	80 MG
**58016005160**	LIPITOR	80 MG
**58016005190**	LIPITOR	80 MG
**58864083430**	LIPITOR	80 MG
**59762015801**	ATORVASTATIN CALCIUM	80 MG
**59762015802**	ATORVASTATIN CALCIUM	80 MG
**60429032601**	ATORVASTATIN CALCIUM	80 MG
**60429032605**	ATORVASTATIN CALCIUM	80 MG
**60429032633**	ATORVASTATIN CALCIUM	80 MG
**60429032690**	ATORVASTATIN CALCIUM	80 MG
**60505267108**	ATORVASTATIN CALCIUM	80 MG
**60505267109**	ATORVASTATIN CALCIUM	80 MG
**60760035630**	ATORVASTATIN CALCIUM	80 MG
**62175089741**	ATORVASTATIN CALCIUM	80 MG
**62175089746**	ATORVASTATIN CALCIUM	80 MG
**63187065690**	ATORVASTATIN CALCIUM	80 MG
**63187090790**	ATORVASTATIN CALCIUM	80 MG
**63304083005**	ATORVASTATIN CALCIUM	80 MG
**63304083090**	ATORVASTATIN CALCIUM	80 MG
**63629336601**	LIPITOR	80 MG
**63629336602**	LIPITOR	80 MG
**63629336603**	LIPITOR	80 MG
**63629336604**	LIPITOR	80 MG
**67877051405**	ATORVASTATIN CALCIUM	80 MG
**67877051490**	ATORVASTATIN CALCIUM	80 MG

© 2023 Davis JW et al. *JAMA Network Open.*

eTable 1. List of Statin Exposures with NDC and Dose

List of Statin Exposures

NDC	Name	Dose
**68084059025**	ATORVASTATIN CALCIUM	80 MG
**68084059095**	ATORVASTATIN CALCIUM	80 MG
**68382025210**	ATORVASTATIN CALCIUM	80 MG
**68382025216**	ATORVASTATIN CALCIUM	80 MG
**68645041854**	ATORVASTATIN CALCIUM	80 MG
**68645046154**	ATORVASTATIN CALCIUM	80 MG
**68645049554**	ATORVASTATIN CALCIUM	80 MG
**69097091105**	ATORVASTATIN CALCIUM	80 MG
**69097091112**	ATORVASTATIN CALCIUM	80 MG
**69097094705**	ATORVASTATIN CALCIUM	80 MG
**69097094712**	ATORVASTATIN CALCIUM	80 MG
**70377003012**	ATORVASTATIN CALCIUM	80 MG
**70377003014**	ATORVASTATIN CALCIUM	80 MG
**71205009890**	ATORVASTATIN CALCIUM	80 MG
**71335029501**	ATORVASTATIN CALCIUM	80 MG
**71335029502**	ATORVASTATIN CALCIUM	80 MG
**71335029503**	ATORVASTATIN CALCIUM	80 MG
**71335058301**	ATORVASTATIN CALCIUM	80 MG
**71335058302**	ATORVASTATIN CALCIUM	80 MG
**71335058303**	ATORVASTATIN CALCIUM	80 MG
**72205002505**	ATORVASTATIN CALCIUM	80 MG
**72205002590**	ATORVASTATIN CALCIUM	80 MG
**76519108403**	ATORVASTATIN CALCIUM	80 MG
**00078035405**	LESCOL XL	80 MG
**00078035415**	LESCOL XL	80 MG
**00093744601**	FLUVASTATIN SODIUM	80 MG
**00093744656**	FLUVASTATIN SODIUM	80 MG
**00378512101**	FLUVASTATIN SODIUM	80 MG
**00378512193**	FLUVASTATIN SODIUM	80 MG
**00781537001**	FLUVASTATIN SODIUM	80 MG
**00781537031**	FLUVASTATIN SODIUM	80 MG
**00781801701**	FLUVASTATIN SODIUM	80 MG
**00781801731**	FLUVASTATIN SODIUM	80 MG
**54569549800**	LESCOL XL	80 MG
**54868460100**	LESCOL XL	80 MG
**00003519510**	PRAVACHOL	80 MG
**00003519533**	PRAVACHOL	80 MG
**00093727010**	PRAVASTATIN SODIUM	80 MG
**00093727098**	PRAVASTATIN SODIUM	80 MG

© 2023 Davis JW et al. *JAMA Network Open.*

eTable 1. List of Statin Exposures with NDC and Dose

List of Statin Exposures

NDC	Name	Dose
**00378055377**	PRAVASTATIN SODIUM	80 MG
**00378828005**	PRAVASTATIN SODIUM	80 MG
**00378828077**	PRAVASTATIN SODIUM	80 MG
**00591001905**	PRAVASTATIN SODIUM	80 MG
**00591001919**	PRAVASTATIN SODIUM	80 MG
**00781523592**	PRAVASTATIN SODIUM	80 MG
**16252052950**	PRAVASTATIN SODIUM	80 MG
**16252052990**	PRAVASTATIN SODIUM	80 MG
**16729001115**	PRAVASTATIN SODIUM	80 MG
**16729001116**	PRAVASTATIN SODIUM	80 MG
**33261095300**	PRAVASTATIN SODIUM	80 MG
**33261095330**	PRAVASTATIN SODIUM	80 MG
**33261095360**	PRAVASTATIN SODIUM	80 MG
**33261095390**	PRAVASTATIN SODIUM	80 MG
**42291066910**	PRAVASTATIN SODIUM	80 MG
**42291066945**	PRAVASTATIN SODIUM	80 MG
**42291066990**	PRAVASTATIN SODIUM	80 MG
**42549049090**	PRAVASTATIN SODIUM	80 MG
**50090278500**	PRAVASTATIN SODIUM	80 MG
**50090278501**	PRAVASTATIN SODIUM	80 MG
**50090320400**	PRAVASTATIN SODIUM	80 MG
**50090320401**	PRAVASTATIN SODIUM	80 MG
**54569651000**	PRAVASTATIN SODIUM	80 MG
**54569651001**	PRAVASTATIN SODIUM	80 MG
**54868463400**	PRAVACHOL	80 MG
**54868557900**	PRAVASTATIN SODIUM	80 MG
**54868557901**	PRAVASTATIN SODIUM	80 MG
**55048059830**	PRAVASTATIN SODIUM	80 MG
**55111027405**	PRAVASTATIN SODIUM	80 MG
**55111027490**	PRAVASTATIN SODIUM	80 MG
**57237016705**	PRAVASTATIN SODIUM	80 MG
**57237016790**	PRAVASTATIN SODIUM	80 MG
**60429037005**	PRAVASTATIN SODIUM	80 MG
**60429037045**	PRAVASTATIN SODIUM	80 MG
**60429037090**	PRAVASTATIN SODIUM	80 MG
**60505132305**	PRAVASTATIN SODIUM	80 MG
**60505132309**	PRAVASTATIN SODIUM	80 MG
**61919073490**	PRAVASTATIN SODIUM	80 MG
**63304059805**	PRAVASTATIN SODIUM	80 MG

© 2023 Davis JW et al. *JAMA Network Open.*

eTable 1. List of Statin Exposures with NDC and Dose

List of Statin Exposures

NDC	Name	Dose
**63304059890**	PRAVASTATIN SODIUM	80 MG
**68084074625**	PRAVASTATIN SODIUM	80 MG
**68084074695**	PRAVASTATIN SODIUM	80 MG
**68180048802**	PRAVASTATIN SODIUM	80 MG
**68180048809**	PRAVASTATIN SODIUM	80 MG
**68258601303**	PRAVASTATIN SODIUM	80 MG
**68258601309**	PRAVASTATIN SODIUM	80 MG
**68382007305**	PRAVASTATIN SODIUM	80 MG
**68382007316**	PRAVASTATIN SODIUM	80 MG
**68462019805**	PRAVASTATIN SODIUM	80 MG
**68462019890**	PRAVASTATIN SODIUM	80 MG
**68788719301**	PRAVASTATIN SODIUM	80 MG
**68788719302**	PRAVASTATIN SODIUM	80 MG
**68788719303**	PRAVASTATIN SODIUM	80 MG
**68788719306**	PRAVASTATIN SODIUM	80 MG
**68788719308**	PRAVASTATIN SODIUM	80 MG
**68788719309**	PRAVASTATIN SODIUM	80 MG
**68788731601**	PRAVASTATIN SODIUM	80 MG
**68788731602**	PRAVASTATIN SODIUM	80 MG
**68788731603**	PRAVASTATIN SODIUM	80 MG
**68788731606**	PRAVASTATIN SODIUM	80 MG
**68788731608**	PRAVASTATIN SODIUM	80 MG
**68788731609**	PRAVASTATIN SODIUM	80 MG
**00006054328**	ZOCOR	80 MG
**00006054331**	ZOCOR	80 MG
**00006054354**	ZOCOR	80 MG
**00006054361**	ZOCOR	80 MG
**00006054382**	ZOCOR	80 MG
**00093715610**	SIMVASTATIN	80 MG
**00093715619**	SIMVASTATIN	80 MG
**00093715656**	SIMVASTATIN	80 MG
**00093715693**	SIMVASTATIN	80 MG
**00093715698**	SIMVASTATIN	80 MG
**00406206903**	SIMVASTATIN	80 MG
**00406206905**	SIMVASTATIN	80 MG
**00406206910**	SIMVASTATIN	80 MG
**00406206960**	SIMVASTATIN	80 MG
**00406206990**	SIMVASTATIN	80 MG
**00781507431**	SIMVASTATIN	80 MG

© 2023 Davis JW et al. *JAMA Network Open.*

eTable 1. List of Statin Exposures with NDC and Dose

List of Statin Exposures

NDC	Name	Dose
**00781507492**	SIMVASTATIN	80 MG
**16252050930**	SIMVASTATIN	80 MG
**16252050950**	SIMVASTATIN	80 MG
**16252050990**	SIMVASTATIN	80 MG
**16590072690**	SIMVASTATIN	80 MG
**16714068501**	SIMVASTATIN	80 MG
**16714068502**	SIMVASTATIN	80 MG
**16714068503**	SIMVASTATIN	80 MG
**16729000710**	SIMVASTATIN	80 MG
**16729000715**	SIMVASTATIN	80 MG
**16729000717**	SIMVASTATIN	80 MG
**21695074230**	SIMVASTATIN	80 MG
**21695074290**	SIMVASTATIN	80 MG
**23490935703**	SIMVASTATIN	80 MG
**23490935706**	SIMVASTATIN	80 MG
**23490935709**	SIMVASTATIN	80 MG
**24658021410**	SIMVASTATIN	80 MG
**24658021430**	SIMVASTATIN	80 MG
**24658021445**	SIMVASTATIN	80 MG
**24658021490**	SIMVASTATIN	80 MG
**24658030410**	SIMVASTATIN	80 MG
**24658030415**	SIMVASTATIN	80 MG
**24658030430**	SIMVASTATIN	80 MG
**24658030445**	SIMVASTATIN	80 MG
**24658030490**	SIMVASTATIN	80 MG
**31722051490**	SIMVASTATIN	80 MG
**35356060030**	SIMVASTATIN	80 MG
**42254016890**	SIMVASTATIN	80 MG
**42571008005**	SIMVASTATIN	80 MG
**42571008090**	SIMVASTATIN	80 MG
**43063008030**	SIMVASTATIN	80 MG
**43063008090**	SIMVASTATIN	80 MG
**43063073330**	SIMVASTATIN	80 MG
**45802029265**	SIMVASTATIN	80 MG
**45802029275**	SIMVASTATIN	80 MG
**50090112100**	SIMVASTATIN	80 MG
**50090112101**	SIMVASTATIN	80 MG
**50268071611**	SIMVASTATIN AVPAK	80 MG
**50268071615**	SIMVASTATIN AVPAK	80 MG

© 2023 Davis JW et al. *JAMA Network Open.*

eTable 1. List of Statin Exposures with NDC and Dose

List of Statin Exposures

NDC	Name	Dose
**50742014010**	SIMVASTATIN	80 MG
**52343002545**	SIMVASTATIN	80 MG
**52343002590**	SIMVASTATIN	80 MG
**54569564000**	ZOCOR	80 MG
**54569611300**	SIMVASTATIN	80 MG
**54569611301**	SIMVASTATIN	80 MG
**54868418100**	ZOCOR	80 MG
**54868418101**	ZOCOR	80 MG
**54868563000**	SIMVASTATIN	80 MG
**54868563001**	SIMVASTATIN	80 MG
**55048077630**	SIMVASTATIN	80 MG
**55048077690**	SIMVASTATIN	80 MG
**55111026805**	SIMVASTATIN	80 MG
**55111026830**	SIMVASTATIN	80 MG
**55111026890**	SIMVASTATIN	80 MG
**55111075010**	SIMVASTATIN	80 MG
**55111075030**	SIMVASTATIN	80 MG
**55111075090**	SIMVASTATIN	80 MG
**55887031830**	SIMVASTATIN	80 MG
**55887031860**	SIMVASTATIN	80 MG
**55887031890**	SIMVASTATIN	80 MG
**60760001930**	SIMVASTATIN	80 MG
**63304079310**	SIMVASTATIN	80 MG
**63304079330**	SIMVASTATIN	80 MG
**63304079350**	SIMVASTATIN	80 MG
**63304079390**	SIMVASTATIN	80 MG
**65862005430**	SIMVASTATIN	80 MG
**65862005439**	SIMVASTATIN	80 MG
**65862005490**	SIMVASTATIN	80 MG
**65862005499**	SIMVASTATIN	80 MG
**66336098630**	SIMVASTATIN	80 MG
**66336098690**	SIMVASTATIN	80 MG
**68084016501**	SIMVASTATIN	80 MG
**68084051401**	SIMVASTATIN	80 MG
**68084051411**	SIMVASTATIN	80 MG
**68115075930**	ZOCOR	80 MG
**68180046503**	SIMVASTATIN	80 MG
**68180046506**	SIMVASTATIN	80 MG
**68180046509**	SIMVASTATIN	80 MG

© 2023 Davis JW et al. *JAMA Network Open.*

eTable 1. List of Statin Exposures with NDC and Dose

List of Statin Exposures

NDC	Name	Dose
**68180048101**	SIMVASTATIN	80 MG
**68180048102**	SIMVASTATIN	80 MG
**68180048103**	SIMVASTATIN	80 MG
**68382006905**	SIMVASTATIN	80 MG
**68382006906**	SIMVASTATIN	80 MG
**68382006910**	SIMVASTATIN	80 MG
**68382006914**	SIMVASTATIN	80 MG
**68382006916**	SIMVASTATIN	80 MG
**68645026354**	SIMVASTATIN	80 MG
**68645047254**	SIMVASTATIN	80 MG
**70377000512**	SIMVASTATIN	80 MG
**70377000514**	SIMVASTATIN	80 MG
**70377000515**	SIMVASTATIN	80 MG
**66582032330**	LIPTRUZET	80 MG-10 MG
**66582032354**	LIPTRUZET	80 MG-10 MG
**00003516811**	PRAVIGARD PAC	81 MG; 20 MG
**00003517311**	PRAVIGARD PAC	81 MG; 40 MG
**00003518311**	PRAVIGARD PAC	81 MG; 80 MG

© 2023 Davis JW et al. *JAMA Network Open.*

eTable 2: Demographics and Comorbidities for the Four Exposure Groups

	Unexposed		Current Statin Only		Recent HT Only		Both	
Variable	Case (n=14,865)	Control (n=156,496)	Case (n=3,364)	Control (n=29,666)	Case (n=1,777)	Control (n=14,573)	Case (n=353)	Control (n=2,855)
**VTE Index Date**								
**2007-2010**	24.11 (3584)	23.80 (37240)	20.30 (683)	22.91 (6796)	31.40 (558)	31.02 (4520)	35.69 (126)	33.42 (954)
**2011-2014**	33.55 (4987)	32.99 (51627)	31.84 (1071)	32.89 (9756)	33.26 (591)	35.28 (5141)	27.76 (98)	33.13 (946)
**2015-2019**	42.34 (6294)	43.21 (67629)	47.86 (1610)	44.21 (13114)	35.34 (628)	33.71 (4912)	36.54 (129)	33.45 (955)
**Age**								
**50-54**	33.81 (5026)	36.91 (57768)	19.62 (660)	19.61 (5818)	55.21 (981)	44.74 (6520)	34.56 (122)	28.76 (821)
**55-60**	35.55 (5284)	33.61 (52595)	35.02 (1178)	34.12 (10123)	27.80 (494)	31.12 (4535)	36.26 (128)	34.47 (984)
**61-65**	30.64 (4555)	29.48 (46133)	45.36 (1526)	46.27 (13725)	16.99 (302)	24.14 (3518)	29.18 (103)	36.78 (1050)
**Region**								
**Northeast**	8.80 (1308)	9.19 (14375)	8.56 (288)	2825 (9.52)	4.22 (75)	4.80 (699)	4.25 (15)	3.89 (111)
**Midwest**	28.50 (4236)	25.90 (40525)	26.49 (891)	26.41 (7836)	24.59 (437)	21.01 (3062)	27.48 (97)	20.95 (598)
**South**	42.86 (6371)	43.56 (68170)	46.08 (1550)	46.83 (13893)	45.98 (817)	51.35 (7483)	49.29 (174)	55.76 (1592)
**West**	19.66 (2922)	20.45 (32009)	18.79 (632)	17.10 (5073)	25.10 (446)	22.73 (3313)	18.98 (67)	19.33 (552)
**Unknown**	0.19 (28)	0.91 (1417)	0.09 (3)	0.13 (39)	0.11 (2)	0.11 (16)	0 (0)	0.07 (2)
**Comorbidities**								
**None**	15.24 (2266)	42.42 (66383)	3.33 (112)	12.56 (3727)	22.85 (406)	36.86 (5371)	7.93 (28)	17.51 (500)
**1-2**	31.30 (4653)	38.97 (60983)	20.90 (703)	43.38 (12869)	38.55 (685)	45.99 (6702)	33.14 (117)	47.46 (1355)
**3+**	53.45 (7946)	18.61 (29130)	75.77 (2549)	44.06 (13070)	38.60 (686)	17.16 (2500)	58.92 (208)	35.03 (1000)
**Cancer**								
**None**	71.11 (10571)	94.40 (147725)	77.41 (2604)	92.88 (27553)	88.75 (1577)	97.24 (14171)	88.67 (313)	97.06 (2771)
**Solid Tumor**	11.86 (1763)	4.77 (7469)	11.30 (380)	6.25 (1854)	5.68 (101)	2.50 (364)	5.10 (18)	2.70 (77)
**Metastatic**	17.03 (2531)	0.83 (1302)	11.30 (380)	0.87 (259)	5.57 (99)	0.26 (38)	6.23 (22)	0.25 (7)
**Trauma**	15.08 (2241)	2.83 (4422)	15.34 (516)	3.34 (990)	14.07 (250)	3.62 (528)	15.01 (53)	3.15 (90)
**Hosp./Surgery**	30.16 (4483)	2.11 (3307)	33.20 (1117)	3.08 (914)	24.93 (443)	2.49 (363)	30.88 (109)	3.08 (88)
**Hypercoag. Cond**	11.75 (1746)	1.19 (1861)	10.49 (353)	1.69 (502)	6.64 (118)	1.09 (159)	5.67 (20)	1.02 (29)
**Varicose Veins**	2.77 (412)	0.86 (1353)	2.47 (83)	0.77 (228)	3.04 (54)	1.22 (178)	1.98 (7)	1.09 (31)
**CAD**	11.48 (1707)	3.39 (5309)	26.19 (881)	12.98 (3852)	6.02 (107)	2.59 (378)	13.60 (48)	8.27 (236)
**Stroke**	6.59 (979)	1.77 (2764)	12.72 (428)	5.99 (1778)	3.55 (63)	1.59 (231)	7.08 (25)	3.96 (113)
**Hyperlipidemia**	36.98 (5497)	28.18 (44094)	89.89 (3024)	85.97 (25503)	32.25 (573)	27.76 (4046)	87.25 (308)	84.06 (2400)
**Smoking**	23.01 (3421)	8.67 (13564)	28.92 (973)	14.91 (4424)	13.90 (247)	6.78 (988)	19.55 (69)	10.05 (287)

© 2023 Davis JW et al. *JAMA Network Open.*
