# Supplementary material for: Statin Use and the Risk of Venous Thromboembolism in Women Taking Hormone Therapy
Source: JAMA Netw Open. 2023 Dec 15;6(12):e2348213. doi: 10.1001/jamanetworkopen.2023.48213 (PMC10724767; doi:10.1001/jamanetworkopen.2023.48213)
Supplement: Supplement 2. — Data Sharing Statement [file jamanetwopen-e2348213-s002.pdf]

Data Sharing Statement

Davis. Statin Use and the Risk of Venous Thromboembolism in Women Taking Hormone Therapy. *JAMA Netw Open*. Published December 15, 2023.
doi:10.1001/jamanetworkopen.2023.48213

Data
Data available: No

Additional Information
Explanation for why data not available: The data are proprietary and available for use by contract only. Statistical code can be provided on reasonable request.
